# Coral garden conservation and restoration: how host taxon and *ex-situ* maintenance affect the microbiome of soft and hard corals

**DOI:** 10.3389/fmicb.2025.1605105

**Published:** 2025-08-06

**Authors:** Marcellina Rola, Márcio A. G. Coelho, Christian Pruckner, Manuela Quiroga-Pérez, Willem Stock, Núria Baylina, Aschwin H. Engelen, Heike Wägele, Ester A. Serrão, Pedro R. Frade

**Affiliations:** ^1^Zoological Department III, Natural History Museum Vienna, Vienna, Austria; ^2^Center for Molecular Biodiversity Research, Leibniz Institute for the Analysis of Biodiversity Change (LIB), Bonn, Germany; ^3^Department of Biology, Evolution and Biodiversity, University of Wuppertal, Wuppertal, Germany; ^4^Centro de Ciências do Mar do Algarve (CCMAR/CIMAR LA), Universidade do Algarve, Faro, Portugal; ^5^Department of Functional and Evolutionary Ecology, University of Vienna, Vienna, Austria; ^6^Phycology Research Group Department of Biology, Ghent University, Ghent, Belgium; ^7^Oceanário de Lisboa, Lisbon, Portugal

**Keywords:** temperate coral gardens, resilience, holobiont, Endozoicomonadaceae, bacteria, gorgonians

## Abstract

Temperate coral gardens are dense coral formations, which support rich marine species diversity, enabling benthic-pelagic coupling. Over the past decades, coral gardens have been increasingly threatened by bottom fishing, oil and gas exploitation, and climate change. Microbiome research bears great potential for assisted resilience in targeted conservation and restoration approaches. Yet, fundamental parameters of the coral garden microbiome remain poorly understood. Here, we provide a first broad record of bacterial communities associated with NE Atlantic coral garden corals and their community changes as response to human maintenance in conservation research. Octocorals (10 species), scleractinians (2 species) and one black coral species, were opportunistically collected from fisheries bycatch at 60–480 m depth around Cape St. Vincent (SW Portugal). Metabarcoding of the 16S-rRNA gene using third-generation sequencing revealed a high microbial host-specificity in the wild-collected coral species analyzed, and supported the importance of bacterial families Endozoicomonadaceae (mean relative abundance ± SE; 28.3 ± 10.5%), Spirochaetaceae (8.2 ± 5.8%) and Spongiibacteraceae (4.6 ± 1.8%). Endozoicomonadaceae were particularly dominant in the octocoral order Malacalcyonacea (67.7 ± 14.5%). The low microbial alpha diversity and limited interspecies differences among the Malacalcyonacea species suggest a conserved microbiome within this group, as compared to orders Scleralcyonacea, Antipatharia, and Scleractinia. Microbial responses to *ex-situ* maintenance of two branching octocoral species, *Eunicella verrucosa* and *Paramuricea* cf. *grayi* (Order Malacalcyonacea), were investigated (1) over 45 days under standardized aquaria conditions in the research station (Ramalhete Marine Station, CCMAR) and (2) over long-term captivity in two public aquaria, Oceanário de Lisboa and Zoomarine. *Eunicella verrucosa* displayed a stronger microbial community shift to short-term captivity (45 days), in contrast to greater microbiome stability in *P.* cf. *grayi*. However, long-term captivity in public aquaria led to microbiome shifts in both species. The strong host specificity of microbial diversity and its response to maintenance indicate that conservation and restoration of coral gardens require taxon-specific strategies.

## 1 Introduction

From continental shelves to bathyal zones, corals act as ecosystem engineers worldwide (Jones et al., 1994), mediating benthic-pelagic coupling and harboring a high biodiversity because they provide shelter from predation and offer nutrient sources and nursery grounds (Freiwald et al., 2004; Roberts et al., 2006; Rossi et al., 2017). Coral gardens are ecologically significant benthic habitats, which can be found in temperate and tropical regions, dominantly composed by octocorals, scleractinians, and black corals (Freiwald et al., 2004; Dias et al., 2020; Nestorowicz et al., 2021). Coral ecosystems are increasingly threatened by the effects of human-induced climate change, such as ocean warming and acidification, as well as through direct impacts from bottom fishing, such as trawling and oil and gas exploitation (Hoegh-Guldberg et al., 2007; Bongaerts et al., 2010; Chapron et al., 2018; Betti et al., 2020; Horta et al., 2025). Coral gardens have not escaped these pressures, which have led to significant habitat degradation and biodiversity loss over the past decades (Souter et al., 2021) and to their designation as Vulnerable Marine Ecosystems (VMEs) (Long et al., 2020). Effective conservation and restoration strategies are therefore critical to mitigate further decline and increase the resilience of these vulnerable ecosystems.

Microbial communities associated with the coral host play a fundamental role in maintaining health and resilience, and ultimately in sustaining a biodiverse and functioning ecosystem (Rohwer et al., 2002; Bourne et al., 2009; Peixoto et al., 2017; Voolstra and Ziegler, 2020). Together with the coral host, bacterial, archaeal, fungal and protist communities form a dynamic and diverse holobiont, mediating important physiological processes such as nutrient cycling (e.g., sulfur and nitrogen), immune defense, and stress adaptation (Rosenberg et al., 2007; Dinsdale et al., 2008; Vega Thurber et al., 2009; Lesser et al., 2019; van Oppen and Blackall, 2019). Algal dinoflagellate photosymbionts belonging to the family Symbiodiniaceae (commonly referred to as zooxanthellae) are key members within the microbiome of tropical shallow-water corals, playing a major role in providing nutrients that predominantly support their growth, reproduction, and survival (Muscatine et al., 1984; Davies, 1991; Trench, 1993), as well as contributing to the calcification process (Goreau, 1959; Gattuso et al., 1999). However, these symbionts are absent in most cold-water and temperate corals occurring in mesophotic to aphotic zones. Due to the limited light availability, these corals rely on suspension feeding of zooplankton, particulate organic matter (POM), and dissolved organic matter (DOM) for nutrition (Freiwald et al., 2004; Roberts et al., 2006).

Among the microbial community of the coral host, some bacterial taxa occur with exceptional abundance and prevalence across coral species and have been identified to provide essential functions for host health and stability, and are thus frequently termed core microbes (Hernandez-Agreda et al., 2017). Typically, microbial species occurring with a high sample prevalence ranging from 30 to 100% of conspecific samples are considered as members of the core microbiome of a particular host (Ainsworth et al., 2015; Hernandez-Agreda et al., 2017; Sweet and Bulling, 2017; Kellogg, 2019; van Oppen and Blackall, 2019). Studies focusing on the coral microbiome have provided valuable insight into the community of essential species-specific microbes. For instance, Endozoicomonadaceae are widely recognized as dominant and functionally significant members of coral microbiomes, particularly in shallow-water and mesophotic corals (Bayer et al., 2013; Ainsworth et al., 2015; Peixoto et al., 2017; Frade et al., 2020; Keller-Costa et al., 2022). Endozoicomonadaceae are known for their metabolic versatility, including the translocation of vitamins to their coral hosts, thus contributing to the overall health and stability of the coral holobiont (McCauley et al., 2023). Other microbes, such as families Pseudomonadaceae, Vibrionaceae, Spongiibacteraceae, Rhodobacteraceae and Flavobacteriaceae, have been commonly associated with corals and identified as part of their core microbiome and are associated with amino acid and vitamin biosynthesis, nitrogen fixation, and sulfur metabolism (La Rivière et al., 2013; Ainsworth et al., 2015; Bourne et al., 2016; Frade et al., 2020; Keller-Costa et al., 2021; Quintanilla et al., 2022).

Efforts to conserve and restore temperate coral gardens are challenged by the fragmented knowledge on their microbial interactions, as compared to their tropical shallow-water counterparts. However, similarly to tropical coral reefs, microbial communities in temperate coral garden corals can be highly host-specific (La Rivière et al., 2013; Ransome et al., 2014; Van De Water et al., 2018; Keller-Costa et al., 2021), and are also highly responsive to environmental conditions (Ransome et al., 2014; Keller-Costa et al., 2021; Reigel and Hellberg, 2023). Microbial flexibility can enhance host survival through rapid adaptation to changing conditions but can also lead to the growth of opportunistic pathogens that result in disease outbreaks and mortality (Hall-Spencer et al., 2007; Rosenberg et al., 2007; Ransome et al., 2014; van Oppen et al., 2015; Bourne et al., 2016). Monitoring shifts in microbial communities during *ex-situ* experimental work can provide insight into how the coral holobiont responds to changing conditions before they cause irreversible damage on host-, and habitat-level, making microbiome analysis a critical tool for conservation and restoration efforts (van Oppen et al., 2015; Peixoto et al., 2017, 2024; Dittami et al., 2021; Corinaldesi et al., 2023; Li et al., 2023). In particular, identifying beneficial microorganisms for corals (BMCs) is a necessary first step for targeted modification of the microbiome, which could ultimately be leveraged to improve coral health and resilience to environmental stress *in-situ* (Peixoto et al., 2017; Peixoto et al., 2019; Voolstra et al., 2024). Among coral conservation and restoration methods, coral transplantation is a successful tool to restore reef health and increase its resilience (Chamberland et al., 2017; Boström-Einarsson et al., 2020; McLeod et al., 2022). In recent years, manipulation of microbiomes associated with marine macroorganisms has been increasingly discussed and initial successes have already been reported (Blackall et al., 2020; Thatcher et al., 2022; Delgadillo-Ordoñez et al., 2024).

A better understanding of microbial communities and their shifts in captivity can provide valuable insights for the application of inoculation techniques, eventually contributing to optimize their efficiency by helping to identify potential key microorganisms or stable microbial consortia and to assess their ecological viability. While studies have provided important baseline data on the microbiomes of some temperate octocorals including host genera like *Eunicella* (Hall-Spencer et al., 2007; La Rivière et al., 2013; Glasl et al., 2017; Keller-Costa et al., 2021), *Paramuricea* (Bayer et al., 2013; La Rivière et al., 2015; Kellogg, 2019), and *Leptogorgia* (Keller-Costa et al., 2021), many coral garden species in the NE Atlantic remain unstudied, leaving gaps in our understanding of their microbial diversity, potential insights into disturbances, and their responsiveness to *ex-situ* maintenance. This study aims to contribute to these efforts by establishing reference microbiomes for coral garden species occurring in the NE Atlantic and assessing their stability close to natural conditions and under aquarium conditions, to enable effective implementation in conservation and restoration efforts. Specifically, we have two main objectives: (1) to characterize baseline microbiomes of NE Atlantic coral garden species and investigate interspecific microbial variability and host specificity, and (2) to assess how *ex-situ* maintenance—(a) short-term (45 days) and (b) long-term (up to 1 year)—affects microbial communities of *Eunicella verrucosa* (Pallas, 1766) and *Paramuricea* cf. *grayi* (Johnson, 1861) and to explore implications for coral conservation and restoration. By integrating microbiome data from both natural and controlled environments, our findings provide critical insights into the microbial ecology of coral gardens and inform future conservation and restoration strategies tailored to taxon-specific microbial dynamics. In particular, understanding microbial dynamics under *ex-situ* conditions may help improve aquarium husbandry protocols and support the development of microbiome-informed coral rehabilitation efforts.

## 2 Materials and methods

### 2.1 Study design, sample collection and processing

**Figure 1:**
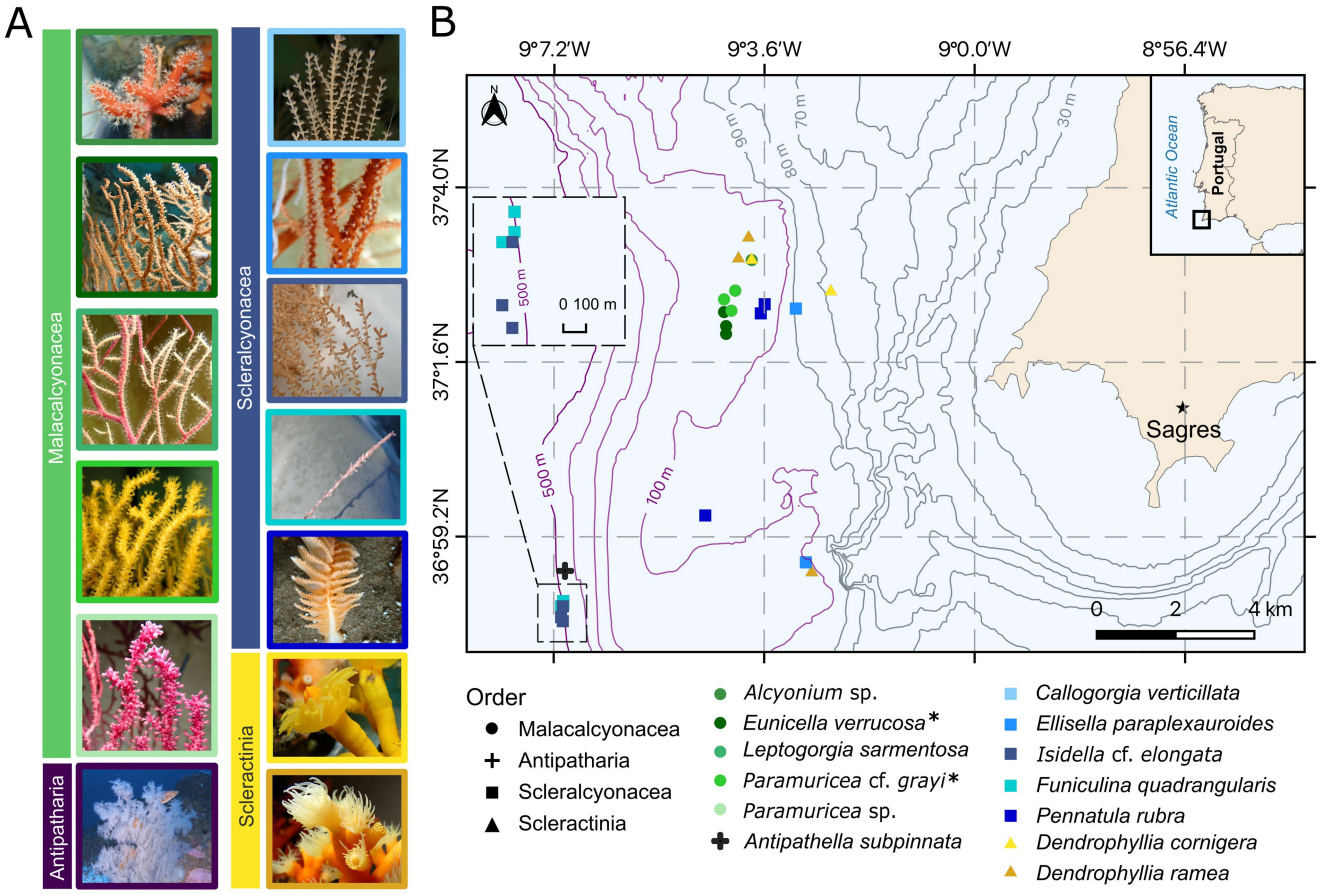
Map of the study area and coral taxa collected (see Table 1 for further details on replicates (*n*), depth (m ± SD), Supplementary Figure S2 for the depth ranges of specimens, and Supplementary Figures S3–S5 on coral groups from different depths). Coral species are indicated by symbol color, and coral orders are distinguished by shape. **(A)** Example images of coral species, color-coded according to species and grouped by coral order. *Callogorgia verticillata* and *Funiculina quadrangularis* images courtesy of Márcio Coelho and *Antipathella subpinnata* image courtesy of CCMAR-FBC. **(B)** Approximate sampling depths of each specimen are shown with bathymetric lines. All species were used for the (1) “*in-situ* microbiome” objective with *n* = 3 replicates per species, except *Alcyonium* sp. (*n* = 1), *C. verticillata* (*n* = 1), and *A. subpinnata* (*n* = 1). Species marked with an asterisk (*) were also used for the (2) “*ex-situ* microbiome” objective [*n* = 3 replicates per species, with *n* = 4 replicates of *Eunicella verrucosa* in the short-term objective (Table 1)]. Notably, species from the Malacalcyonacea order were only collected between 60–120 m, whereas two scleralcyonacean species (*Funiculina quadrangularis* and *I.* cf. *elongata*), along with the black coral *A. subpinnata*, were exclusively collected at 400–500 m depth.

Coral colonies included in this study were obtained through accidental by-catch from a collaborating fisher crew that operates with bottom-set gillnets on a small (<12 m hull length) vessel around Cape St. Vincent (Sagres, SW Portugal) (Dias et al., 2020) (Figure 1). Cape St. Vincent is characterized by complex oceanographic conditions, including strong upwelling events, high nutrient availability, and dynamic temperature gradients (Relvas and Barton, 2002; Haynes and Barton, 1990). The local coral community of SW Portugal is mainly dominated by soft coral taxa, such as the malacalcyonacean species *Eunicella verrucosa*, *Paramuricea* cf. *grayi*, *Leptogorgia sarmentosa* (Esper, 1794), and scleralcyonacean species like *Ellisella paraplexauroides* Stiasny, 1936, the black coral *Antipathella subpinnata* (Ellis and Solander, 1786), and the scleractinian corals *Dendrophyllia cornigera* (Lamarck, 1816) and *D. ramea* (Linneaus, 1758) (Dias et al., 2020; Nestorowicz et al., 2021).

This study was conducted with two main objectives investigating (1) the “*in-situ* microbiome” variation of coral garden corals and (2) “*ex-situ* microbiome” variation under aquarium conditions. The focus of the microbiome analysis was laid on the bacterial domain. In objective (1) “*in-situ* microbiome,” we focused on investigating the natural bacterial community composition and structure of 13 coral species of two octocoral orders (Malacalcyonacea and Scleralcyonacea), the black coral order (Antipatharia), and the stony coral order (Scleractinia) (Figure 1; Table 1). These corals were directly sampled from the fishing net aboard the fishing vessel (19th and 29th of August 2022) and this objective is defined as “natural microbiome” henceforth. The results of this objective will be presented and referred to as “wild” condition.

**Table 1:** Overview of the samples and objectives of this study.

Objective	Species	Species origin	Sampling location		Sampling time	
Natural microbiome	Coral species and sampling coordinates shown in Figure 1 *Alcyonium* sp. (*n* = 1; 93.269 ± 0 m) *Eunicella verrucosa* (*n* = 3; 85.405 ± 1.782 m) *Leptogorgia sarmentosa* (*n* = 3; 73.152 ± 6.509 m) *Paramuricea* cf. *grayi* (*n* = 3; 85.954 ± 00.862 m) *Paramuricea* sp. (*n* = 3; 65.837 ± 2.586 m) *Antipathella subpinnata* (*n* = 1; 437.083 ± 0 m) *Callogorgia verticillata* (*n* = 1; 464.515 ± 0 m) *Ellisella paraplexauroides* (*n* = 3; 71.323 ± 15.032 m) *Isidella cf. elongata* (*n = 3; 464.515* ± 3.449 m) *Funiculina quadrangularis* (*n* = 3; 464.515 ± 0 m) *Pennatula rubra* (*n* = 3; 92.720 ± 10.091 m) *Dendrophyllia cornigera* (*n = 3; 69.494* ± 9.6 m) *Dendrophyllia ramea* (*n* = 3; 89.611 ± 8.224 m)	Cape St. Vincent, Sagres, Portugal	Cape St. Vincent, Sagres, Portugal		19th, 29th of August 2022	
2. Captivity Effect 2.1. Over—Time	*Eunicella verrucosa* (*n* = 4) *Paramuricea* cf. *grayi* (*n* = 3)	Cape St. Vincent, Sagres, Portugal	**Ramalhete** Marine Station, CCMAR, Faro, Portugal			**Day 0**
						**Day 4**
						**Day 45**
						(data from 1. Natural Microbiome used as reference data) **wild**
2.2. Long—Term (Location)	*Eunicella verrucosa* (*n* = 3) *Paramuricea* cf. *grayi* (*n* = 3)	Cape St. Vincent, Sagres, Portugal		**Zoomarine,** Albufeira, Portugal	≈ 1 month	
				**Oceanário** de Lisboa, Lisbon, Portugal	≈ 1 year	
				(data from 2.1. Over—Time used as reference data) **Ramalhete**	Day 45	
				(data from 1. Natural Microbiome used as reference data) **wild**	19th, 29th of August 2022	

Sample size (*n*) refers to the number of sequenced specimens per species. The variable names used when the results are reported and are used in the figures throughout this paper are highlighted in bold. Sampling depth [mean depth (m) ± SD] is shown for samples of objective 1 (Natural Microbiome) and for objective 2 (Captivity Effect), no sampling depth is provided, as these were collected from aquarium systems. Further details for the (1) Natural microbiome objective, on respective order and sampling location (coordinates) are visualized in Figure 1.

In (2) “*ex-situ* microbiome” we assessed how bacterial communities of two locally dominant gorgonians, *E. verrucosa* and *P.* cf. *grayi* [yellow lineage *sensu* (Coelho et al., 2023)] of Order Malacalcyonacea, responded to (2.1.) *ex-situ* conditions within the first 45 days of captivity in the tank system of Ramalhete Marine Station (University of Algarve—CCMAR, Faro, Portugal). Here, the natural microbiome samples of *E. verrucosa* and *P.* cf. *grayi* were included as a reference. This objective is referred to as “short-term captivity.” We also assessed microbial changes in *E. verrucosa* and *P.* cf. *grayi* to (2.2.) *ex-situ* conditions in two public aquaria for ~3 months (Zoomarine, Albufeira, Portugal) and ~1 year (Oceanário de Lisboa, Lisbon, Portugal). Here, we also included the samples of day 45 from the over-time, as well as the samples from the natural microbiome objective, and refer to this objective as “long-term captivity.” The sampling locations are hereafter referred to as Oceanário, Ramalhete, Zoomarine, and wild.

Coral samples were sprayed with filtered [0.2 μm Millipore Sterivex filters (Merck KGaA, Darmstadt, Germany)] and autoclaved seawater prior to sampling for genetic analysis in order to remove loosely associated microbes. Only specimens considered healthy based on visual inspection (absence of tissue necrosis, or weak coloration) were sampled. About 1.5 cm fragments of soft coral specimen branch tips were collected and 5 cm pieces of scleractinian corals (Supplementary Figure S1). From branching octocoral species, and the black coral, a small, apical branchlet fragment was taken. From the scleractinian corals, a fragment with one to three polyps was taken and from the sea pen, a few polyp leaves were sampled. The samples were preserved with aqueous DESS solution (20% salt-saturated DMSO, 0.25 M EDTA and saturated NaCl, adjusted to pH 8.0) and kept at −20°C until processing. In addition to coral samples, we also took water samples from the tank systems where corals were kept in captivity (Oceanário, Ramalhete, Zoomarine). Water samples were taken by filtering 1 L aquarium water through 0.2 μm Millipore Sterivex filters (Merck KGaA, Darmstadt, Germany) and filters were subsequently flash frozen in liquid nitrogen and stored at −80°C.

### 2.2 Objective (1) *in-situ* microbiome of Atlantic coral garden species

A total of 13 coral species were sampled. This included six biological replicates of the selection of 13 representative coral garden taxa following the regional species composition outlined by Dias et al. (2020), of which only one specimen each of *Alcyonium* sp. Linneaus, 1758 (Order Malacalcyonacea), *Antipathella subpinnata* (Order Antipatharia), and *Callogorgia verticillata* [(Pallas, 1766), Order Scleralcyonacea] was included in the natural microbiome objective, due to limited availability. Three of the six biological replicates per coral garden species and the three species with one biological replicate were selected for sequencing (Table 1). Tissue of each coral specimen was sampled aboard the vessel, immediately after reaching the surface and was subsequently preserved in DESS for genetic analysis (see above). The remaining colony or fragment of the colony was tagged and kept as taxonomic vouchers [except the sea pen *Pennatula rubra* Ellis, 1764 and soft coral *Alcyonium* sp.]. In total, 33 coral samples from 13 coral garden species were selected for microbiome analysis and sequenced.

### 2.3 Objective (2) effect of *ex-situ* maintenance on the microbiome of coral garden corals

#### 2.3.1 Assessment of microbiome changes over short-term captivity (objective 2.1.)

To assess how the microbiome changed over short-term captivity, *E. verrucosa* and *P.* cf. *grayi* colonies collected on the fishing vessel were maintained in a holding tank onboard with continuous water recycling until reaching the harbor. Once on land, the corals were transferred into buckets filled with seawater from the Sagres harbor and transported to Ramalhete where they were maintained for 45 days in a semi-closed system of multiple aquaria connected to a SUMP filter with water recirculation through a chiller to control water temperature (~average of 16°C). The room was climate-controlled and was kept at ~16°C. The incoming seawater was pumped from an earthen deposit tank in the Ria Formosa lagoon, filtered through sand filters (calibrated to 0.2 a 0.6 mm diameter and flow speed of 30m^3^/m^2^/h), additional thread filters (125 micra) and passed under UV light (UV Steriliser P1 55W). Salinity was monitored throughout the experiment and the mean salinity was at 36.60 ± 0.64 PSU. Transport seawater was preserved directly upon arrival at the Ramalhete station (day 0) for further analysis (wild sample). Additionally, water of the selected aquarium tank system was sampled before the start of the experiment (day 0), every second day for the first 10 days (days 2, 4, 6, 8, 10), and once a week until day 45 thereafter (days 17, 24, 31, 38, and 45). In addition to the water samples, branchlets of the coral colonies were also sampled throughout the experiment. For this study only samples collected on day 0, 4, and 45 were selected for analysis (Table 1). Octocorals were fed every day on weekdays with live cultures of rotifers produced inhouse and/or with frozen rotifers and copepods [*Calanus* sp., purchased from Tropical Marine Centre (TMC)]. The aquaria were cleaned several times per week.

#### 2.3.2 Estimating the long-term effect of captivity in different aquarium systems (objective 2.2.)

Three separate coral colonies of *E. verrucosa* and *P.* cf. *grayi*, collected as bycatch by the same Sagres fishing vessel and donated to public aquaria, were sampled from the public aquaria: after 3 months in culture in Zoomarine in Albufeira and after 1 year in culture in Oceanário (Table 1). Despite having been collected by the same crew from the same sampling sites in Sagres, the exact location of collection could not be reconstructed. In Zoomarine, both gorgonians were kept in one exhibition tank, together with other common coral species from SW Portugal, such as *Leptogorgia sarmentosa*. In Oceanário, both corals were kept in separate tanks in the quarantine area. Branches of three separate coral colonies per species and one water sample were collected from the respective aquarium tank in Zoomarine, and the two tanks in Oceanário. In total for objective (2), 36 coral samples, and 15 water samples were selected for sequencing and analysis. For (2.1.) 25 coral and 12 water samples and for (2.2.) 12 coral samples and 3 water samples were included in this study.

#### 2.3.3 DNA extraction and 16S ribosomal RNA gene sequencing

Microbial DNA extraction and subsequent 16S ribosomal RNA gene sequencing were conducted on a total of 84 coral and water samples using the ZymoBIOMICS™ DNA Miniprep Kit. To access the water filter paper within the 0.2 μm Millipore Sterivex filters (Merck KGaA, Darmstadt, Germany) under sterile conditions, we followed the procedure to open the filter case and process the filter paper outlined by Cruaud et al. (2017). DNA extraction sessions included negative extraction controls. The tissue lysis step of the ZymoBIOMICS™ DNA Miniprep protocol was modified by adding 20 μL proteinase K (Qiagen GmbH, Hilden, Germany) and 42 μL 20% SDS (Carl Roth GmbH, Karlsruhe, Germany) followed by a 30-min incubation at 60°C. The remainder protocol was conducted according to the manufacturer’s instructions. For DNA extraction, the coral fragment was homogenized to control for microbial differences between coral structures (tissue, mucus, skeleton). Approximately 1 g of coral tissue (following instructions) and ¼ of the water filter was used. Extracted DNA was shipped on dry ice to the Ghent University for 16S rRNA gene sequencing using the Oxford Nanopore MinION technology (Oxford Nanopore Technologies, Oxford, United Kingdom). The sequencing procedure was conducted following an established workflow described in van der Loos et al. (2021). For the amplification of the full-length 16S rRNA gene, the primer pair 27_BCtail-FW and 1492R_BCtail-RV was used [27F_BCtail-FW: (TTTCTGTTGGTGCTGATATTGC_AGAGTTTGATCMTGGCTCAG) and 1492R_BCtail-RV: (ACTTGCCTGTCGCTCTATCTTC_CGGTTACCTTGTTACGACTT)] (van der Loos et al., 2021). This primer pair featured a 5′ extension for the barcoding PCR step. PCR amplification of the 16S rRNA gene was performed in a thermocycler using the Phire Tissue direct PCR Master Mix (Fisher Scientific GmbH, Schwerte, Germany) and the following PCR program was conducted: 3 min at 98°C, followed by 30 cycles of 8 s at 98°C, 8 s at 60°C, and 30 s at 72°C, concluding with a final extension of 3 min at 72°C. Multiplexing to prepare barcoded sequencing libraries was executed using the PCR Barcoding Expansion Pack 1-96 (Oxford Nanopore Technologies, Oxford, United Kingdom). Library preparation was conducted using the sequencing kit SQK-LSK109 following the manufacturer specifications (Oxford Nanopore Technologies, Oxford, United Kingdom). The sequencing run was carried out for 48 h on a MinION device using a R9.4.1 flow cell (Oxford Nanopore Technologies, Oxford, United Kingdom).

#### 2.3.4 Read processing and statistical analyses

Sequencing and basecalling were conducted using the Oxford Nanopore Technologies (ONT) software. MinKNOW (version 22.10.7) was used for data acquisition and run control, alongside Bream (version 7.3.2) for device coordination. The sequencing configuration was set to version 5.3.7. Basecalling was performed with Guppy (version 6.3.8) using the super-accurate mode, ensuring high read accuracy. Additionally, MinKNOW Core (version 5.3.1) facilitated overall system management and data processing (van der Loos et al., 2021). Sequencing reads underwent quality control, including chimeric read removal using minimap (version 2.17) and yacrd (0.3.0) and filtering for length (ranging from 1,200 to 1,700 bp) using NanoFilt (2.8.0). Read length range was set to retain near full-length 16S rRNA sequences while accounting for MinION Nanopore specific variability in read lengths, structure, and quality (Calus et al., 2018). Filtered sequences were subsequently used for taxonomic assignment, using Kraken 2.1.2 with the SILVA reference database (version 138.1). Kraken2 increases assignment reliability due to a low sensitivity to individual base errors, by performing k-mer based classification (Lu and Salzberg, 2020), which is therefore well-suited for the high basecalling error rate of Nanopore reads (Calus et al., 2018). Statistical analysis and data visualization were carried out in R using the following packages: phyloseq (version 1.46.0), vegan (version 2.6–8), ggplot (version 3.5.1), ampvis2 (version 2.8.4), biom (version 0.4.0), tidyverse (version 2.0.0), car (version 3.1-3), dplyr (version 1.1.4), and VennDiagram (version 1.7.3). Three significance levels were applied and reported in the results section: *p* < 0.001 (***), *p* < 0.01 (**), and *p* < 0.05 (*), whereas exact *p*-values are provided in Supplementary material. For further analysis, data was processed by removing chloroplast and mitochondrial reads, singletons, and rare OTUs with relative abundances below 1% of the total sequences.

To compare alpha diversity among samples, the sequence data was rarefied to 25,209 reads per sample. The chosen rarefaction depth corresponds to the sample with the lowest retained sequencing depth after filtering out rare OTUs. Alpha diversity was assessed using the Observed, Chao1, and Shannon-Weaver diversity indices. These indices were visualized using min-max plots (for *n* = 3 replicates per coral species). In the results section, we report alpha diversity while focusing on the Shannon-Weaver index as the representative metric. Additional results for the Observed and Chao1 indices are provided in Supplementary material. Significant differences in alpha diversity were evaluated with parametric analysis of variance (ANOVA), or non-parametric KW in case of heteroscedasticy. When significant ANOVA results were obtained, we conducted *post hoc* comparisons using Tukey’s Honest Significant Difference (HSD) test at 95% confidence level to assess pairwise differences in alpha diversity between groups. Additionally, we applied the emmeans function in R to obtain estimated marginal means and pairwise contrasts of the assessed pairwise differences in Shannon-Weaver index. The emmeans represent model-adjusted means of the Shannon-Weaver index, accounting for variability and potential imbalances in sample sizes. Venn diagrams were used to illustrate the distribution of unique, shared, and ubiquitous OTUs.

The community composition of the microbiome was assessed, focusing on the presence of OTUs and their relative abundances, while for the community structure, we focused on the organization of units (OTUs, species, groups) over space. Beta diversity was computed using Bray-Curtis dissimilarities which were plotted in a principal coordinate analysis (PCoA). This ordination method was used to visualize the structuring of microbial communities according to host species and orders (natural microbiome), as well as according to aquarium keeping over-time (short-term) and between different aquarium locations (long-term). Differences in community structure were statistically tested using permutational analysis of variance (PERMANOVA) with 999 permutations. The rarefied data was transformed into relative abundances prior to analyzing beta-diversity. Homogeneity of group dispersions (variances) was tested using the PERMDISP (Anderson, 2006; Anderson et al., 2006) procedure with the betadisper function.

To investigate, whether changes in the microbial communities of each coral species correlate with microbial changes in the water column, a Mantel’s test was used. Microbial indicators were identified through an indicator value analysis with 1,000 permutations using the R package indicspecies (Cáceres and Legendre, 2009). DESeq2 was used to identify significant changes in bacterial taxa composition between conditions (over-time and locations). The core microbiome across coral garden species and of each gorgonian *E. verrucosa* and *P.* cf. *grayi* (natural microbiome), as well as over-time and over different locations per species, was determined using the microbiome package (version 1.46.0) in R. At varying detection thresholds (0.1–2.5%) the prevalence of each OTU was assessed and visualized in a heatmap using the plot_core function. A taxon was considered part of the core core microbiome when present in at least 50% of the samples (Ainsworth et al., 2015; Kellogg, 2019).

## 3 Results

A total of 17.54 Gb of sequencing data was generated using MinION technology and an average of 56451 ± SE 1418 reads per sample were obtained after quality filtering and data processing.

### 3.1 Coral garden *in-situ* microbiome is host-specific

#### 3.1.1 Alpha- and beta-diversity vary among coral garden species and orders

**Figure 2:**
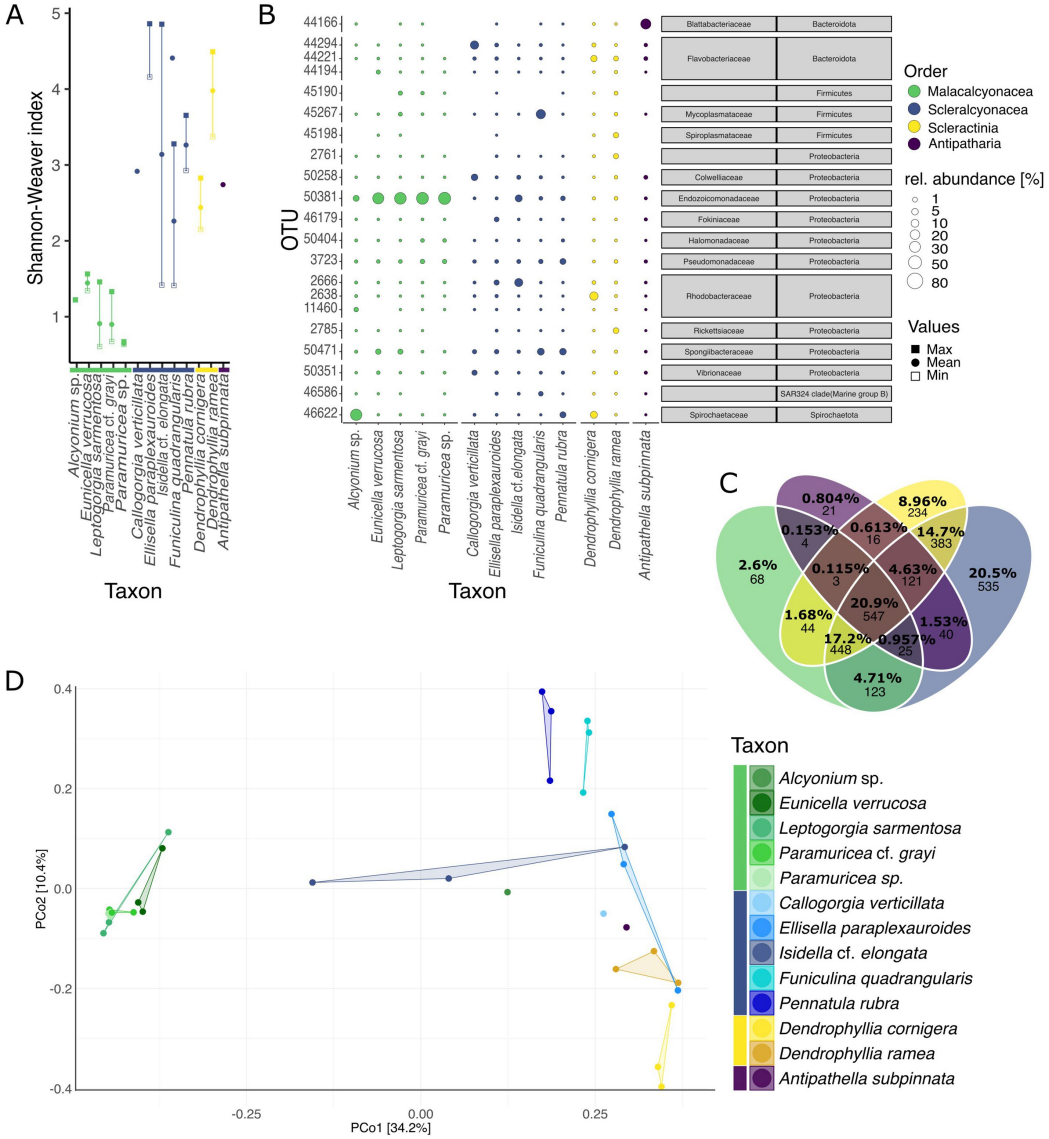
Natural microbiome of coral garden species. **(A)** Minimum (open square), maximum (filled square), and mean (filled circle) alpha diversity (Shannon-Weaver index) for different species (coral orders are indicated by color). **(B)** Relative abundances (in percent) of the 3 most abundant OTUs per coral species (total of 21 OTUs among all coral species). Abundances are indicated by the bubble size and orders are color-coded as in Figure 1 (values in Supplementary Tables S11, S12). Gray boxes give microbial taxonomic affiliation. **(C)** Venn diagram showing the percentages and count of unique, shared, and ubiquitous OTUs between the four orders analyzed, Malacalcyonacea, Scleralcyonacea, Scleractinia, and Antipatharia (indicated by color). Percentages represent the relative contribution of each subset (unique, shared, and ubiquitous between orders) to the total number of OTUs across the four orders. Venn diagrams of unique, shared, and ubiquitous OTUs between species within each order, see Supplementary Figure S3. **(D)** Principal coordinates analysis based on Bray Curtis dissimilarities (999 permutations). Species and orders are displayed by color and each data point in the PCoA represents one sample.

Alpha diversity differed significantly among coral taxa at both the species and order levels (ANOVA, species: Shannon-Weaver index, *F* = 8.481, Df = 12, *p* < 0.001; order: Shannon-Weaver index, *F* = 16.51, Df = 4, *p* < 0.001; for further results see Supplementary Tables S1–S8). The orders Scleractinia (2 species) and Scleralcyonacea (5 species) displayed significant pairwise differences to Malacalcyonacea (5 species), with a significantly higher alpha diversity (TukeyHSD, mean pairwise difference (Shannon-Weaver index) = 2.22 ± 0.04, adjusted *p* < 0.001; Supplementary Table S8). Malacalcyonacea species had the second lowest number of unique OTUs (2.6%, 68 OTUs, Figure 2C; Supplementary Figure S6) and the lowest overall alpha diversity (estimated marginal means = 0.992 ± 0.240, Figure 2C; Supplementary Table S7). The lowest number of unique OTUs was found for the order Antipatharia, which was represented by a single species, *A. subpinnata* and a single sample. Species of the orders Scleractinia and Scleralcyonacea showed the highest interspecific variability in alpha diversity with a high variation between the minimum and maximum alpha diversity (Figure 2A). Scleralcyonacean species also showed the highest number of unique OTUs (20.5%, 535 OTUs, Figure 2C; Supplementary Figure S6) and the highest alpha diversity compared to all other coral orders (estimated marginal means = 3.24 ± 0.24, Figure 2C; Supplementary Table S7). Within that order (Scleralcyonacea), the highest variation between minimum and maximum alpha diversity was found for the biological replicates of the bamboo coral *I.* cf. *elongata* (Esper 1788) (Figure 2A). Among all coral species studied, *E. paraplexauroides* (Scleralcyonacea) was found to have the highest alpha diversity (estimated marginal means = 4.41 ± 0.41, Supplementary Table S5; Figure 2A), with significant group differences further supported in comparison to the malacalcyonacean taxa *Alyonium* sp. (represented by one sample), *E. verrucosa*, *L. sarmentosa*, *P.* cf. *grayi*, and the scleralcyonacean *F. quadrangularis* (Pallas 1766) [mean pairwise difference (Shannon-Weaver index) = 3.1 ± 0.63, adjusted *p* < 0.05, Supplementary Table S6].

Beta diversity also differed significantly among coral species and orders (PERMANOVA, species: *F* = 2.5385, Df = 8, *p* < 0.001; orders: *F* = 8.9875, Df = 4, *p* < 0.001, Supplementary Table S9) leading to a distinct separation between species and orders as seen by ordination (Figure 2D). Differences in community structure may be explained by dispersion differences among groups (PERMDISP, *F* = 8.0678, Df = 4, *p* < 0.05, Supplementary Table S9). Malacalcyonacea species clustered together showing a high level of similarity among samples and a distinct separation from other coral species and orders, except for *Alcyonium* sp. (Figure 2D), which was represented by one single sample. In contrast to the Order Malacalcyonacea, species of the order Scleralcyonacea exhibited greater dissimilarity both among species and across samples within species. The highest intraspecific variation among samples of *I.* cf. *elongata* was also observed in terms of community similarity (Figure 2D).

#### 3.1.2 Community composition, core microbes and indicator microbes of coral garden species

The composition of the most abundant microbes, and their relative abundances differed among coral species and orders (Figure 2B). The phylum Proteobacteria dominated the coral microbiome across all studied species, with a particularly high abundance of the gammaproteobacterial family Endozoicomonadaceae (37.73 ± 38.89%, Figure 2B; Supplementary Table S10). Endozoicomonadaceae (OTU 50381, *Endozoicomonas*) also showed a high sample prevalence in the coral microbiome (100% prevalence until 0.2% detection threshold, Supplementary Figure S7). The second most prevalent microbe with a generally high abundance across all studied coral species was classified within the family Pseudomonadaceae (OTU 3723, *Pseudomonas*) (90% prevalence at 0.1% detection threshold, decreasing to 30% until 2.5% detection threshold, Supplementary Figure S7, mean relative abundance 2.89 ± 3.46%, Figure 2B; Supplementary Table S10).

Coral orders and species also exhibited differences in the composition and abundance of the most abundant microbes (Figure 2B; Supplementary Tables S11, S12). The Malacalcyonacea group displayed a largely homogeneous composition and abundance of abundant microbes across species of this order, with one single OTU (50381, Endozoicomonadaceae) accounting for 76.45 ± 21.72% mean relative abundance (Figure 2B; Supplementary Table S12). *Alcyonium* sp. was the only species of the Malacalcyonacea order that had a different microbial community composition, with a lower abundance of Endozoicomonadaceae (11.07%, *n* = 1, Supplementary Table S11) and having OTU 46622 from the family Spirochaetaceae (Spirochaetota) as the most abundant microbe (75.08%, *n* = 1, Supplementary Table S11). Since this coral species was only represented by one sample, further research is needed to confirm these observed microbial patterns. In contrast to the Malacalcyonacea order, Scleralcyonacea species exhibited a more heterogeneous composition and abundance of the most abundant microbes (Figure 2B). The most abundant microbe in *E. paraplexauroides* (OTU 2666, Rhodobacteraceae) did not exceed 10% relative abundance (7.17 ± 3.07%, Figure 2B; Supplementary Table S11). Other members of the Scleralcyonacea group had different microbes of particularly high abundance, such as OTUs belonging to the family Flavobacteriaceae in *C. verticillata* (32.63%, *n* = 1, Figure 2B; Supplementary Table S11), Rhodobacteriaceae in *I.* cf. *elongata* (30.92 ± 33.88%), Mycoplasmataceae in *F. quadrangularis* (44.33 ± 38.74%), and Spongiibacteraceae in *P. rubra* (16.77 ± 14.76%). Among the scleractinian genus *Dendrophyllia*, Rhodobacteraceae was most abundant in *D. cornigera* (31.12 ± 24.37%) and Rickettsiaceae in *D. ramea* (9.29 ± 8.39%). The most abundant OTU in the black coral (*A. subpinnata*) was assigned to the microbial family Blattabacteriaceae (57.88%, *n* = 1).

Taxon-specific indicator OTUs were identified for eight out of the 13 coral species studied (IndVal analysis, Supplementary Figure S8). In general, microbial indicators belonged to Gammaproteobacteria (e.g., Saccharospirellaceae, Nitrincolaceae, Heliaceae, Oxalobacteraceae, Xanthomonadaceae) and Alphaproteobacteria (e.g., Rhodobacteraceae, Sphingomondaceae, Devosiaceae, Anaplasmataceae) within the phylum Proteobacteria. The highest number of significant indicator OTUs (22 OTUs) were identified for *E. paraplexauroides* (Scleralcyonacea), which included OTUs assigned to the family Micrococcaceae (Actinobacteria), Ruminococcaceae (Firmicutes), and Rhodobacteraceae (Proteobacteria). Within the studied malacalcyonacean species, 17 indicator OTUs were identified for *E. verrucosa*, belonging to the Phylum Proteobacteria, such as Devosiaceae and Xanthobacteraceae. No significant indicator OTUs were identified for *P. cf. grayi*. Within the scleractinian order, *D. cornigera* had seven indicator OTUs, with the top five most indicative OTUs belonging to the family Desulfurivibrionaceae (Desulfobacterota), while no indicator microbes were detected in *D. ramea*. No significant indicator OTUs were found for the black coral *A. subpinnata*, which was only represented by one sample.

### 3.2 Coral garden *ex-situ* microbiome is affected by aquarium maintenance

#### 3.2.1 Variation of microbial alpha and beta diversity in *Eunicella verrucosa* and *Paramuricea* cf. *grayi* under *ex-situ* conditions

##### 3.2.1.1 Effect of short-term captivity on the microbiome of *Eunicella verrucosa* and *Paramuricea* cf. *grayi*

**Figure 3:**
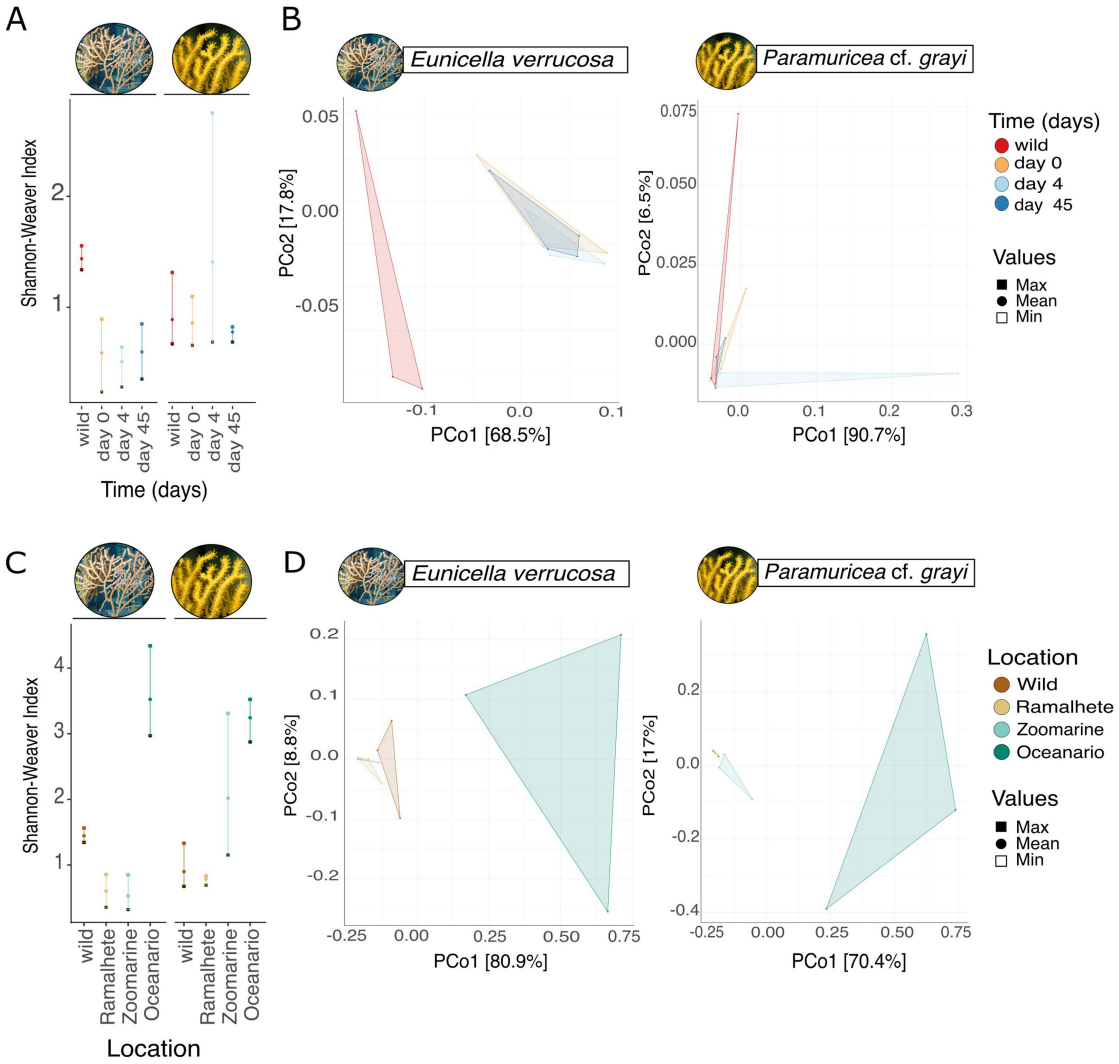
Effect of captivity on the microbiome of *Eunicella verrucosa* and *P.* cf. *grayi*. Effect of short-term captivity over 45 days in Ramalhete on the microbial **(A)** alpha diversity (Shannon-Weaver index) showing the minimum (open square), maximum (filled square) and mean (filled circle) and **(B)** beta diversity. Principal coordinates analysis based on Bray Curtis dissimilarities (999 permutations). The sampling days are displayed by color and each data point in the PCoA represents one sample. Effect of long-term captivity in different locations, the wild (Sagres), Ramalhete on day 45, Zoomarine, and Oceanário on the microbial **(C)** alpha diversity (Shannon-Weaver index) showing the minimum (open square), maximum (filled square) and mean (filled circle) and **(D)** beta diversity. PCoA based on Bray Curtis dissimilarities with 999 permutations shows group separations, with sampling locations being displayed by color and each data point representing one sample.

Within 45 days of captivity in Ramalhete, the microbiome of *E. verrucosa* was affected (ANOVA, Shannon-Weaver index: *F* = 14.11, Df = 3, *p* < 0.001, Figure 3A; Supplementary Tables S13, S14). No significant effect on the microbiome of *P*. cf. *grayi* was found (ANOVA, Shannon-Weaver index: *F* = 0.626, Df = 3, *p* > 0.05, Figure 3A; Supplementary Tables S13, S14). Multiple comparison testing showed, that alpha diversity in the wild *E. verrucosa* was significantly higher than the alpha diversity of the coral kept in the aquarium on day 0, 4, and 45 [TukeyHSD: pairwise difference (Shannon-Weaver index) = 0.880 ± 0.16, *p* = 0.001, Supplementary Tables S15–17]. Wild samples of *E. verrucosa* also had a higher number of unique OTUs, compared to samples kept in captivity and taken over time (Venn diagram, wild: 12.3%, 146 OTUs, day 0: 11.7%, 139 OTUs, day 4: 7.25%, 86 OTUs, and day 45: 7.33%, 87 OTUs, Supplementary Figure S9). The number of unique OTUs was not higher in the wild samples of *P*. cf. *grayi*, compared to samples taken over time. Samples taken on day 4 had the highest number of unique OTUs (wild: 7.31%, 98 OTUs, day 0: 6.34%, 85 OTUs, day 4: 18.6%, 250 OTUs, day 45: 5.59%, 75 OTUs, Supplementary Figure S9).

PERMANOVA revealed a significant shift in the overall microbial community composition of *E. verrucosa* samples kept in captivity over time compared to wild samples (PERMANOVA, *F* = 4.215, Df = 3, *p* < 0.01, Supplementary Table S18). This shift is also illustrated by the clear separation observed in the principal coordinates analysis (PCoA; Figure 3B; Supplementary Table S18). However, no significant dispersion differences were detected among groups (PERMDISP, *F* = 3.639, Df = 3, *p* = 0.054, Figure 3B; Supplementary Table S18). Concurrent with alpha diversity, no clear separation for *P*. cf. *grayi* samples from the wild and over time were found (PERMANOVA, *F* = 0.852, Df = 3, *p* > 0.05; Supplementary Table S18), with groups also not showing significant differences in dispersion (PERMDISP, *F* = 0.647, Df = 3, *p* < 0.05, Supplementary Table S18).

##### 3.2.1.2 Effect of long-term captivity in different aquaria on the microbiome of *Eunicella verrucosa* and *Paramuricea* cf. *grayi*

When comparing samples from the wild, with samples from day 45 of the short-term objective in Ramalhete, the Zoomarine, and Oceanário samples, we observed significant differences in microbial alpha diversity for both *E. verrucosa* and *P*. cf. *grayi* (ANOVA, *E. verrucosa*: Shannon-Weaver index, *F* = 41.38, Df = 3, *p* < 0.001; *P*. cf. *grayi*: Shannon-Weaver index, *F* = 10.29, Df = 3, *p*-value < 0.01, Figure 3C; Supplementary Tables S19, S20). Multiple group comparisons revealed a higher alpha diversity in *E. verrucosa* samples from Oceanário, compared to all other locations (the wild samples, Ramalhete, and Zoomarine [TukeyHSD, mean pairwise difference (Shannon-Weaver index) = 2.66 ± 0.51, *p* < 0.001, Figure 3C; Supplementary Tables S21–23)]. Similarly, *P*. cf. *grayi* samples taken from Oceanário, also showed a higher alpha diversity, compared to the wild samples and samples from Ramalhete [TukeyHSD, mean pairwise difference (Shannon-Weaver index) = 2.40 ± 0.08, *p* < 0.01, Figure 3C; Supplementary Tables S21–23]. However, *P*. cf. *grayi* samples from Zoomarine did not differ significantly in alpha diversity compared to samples from Oceanário, while showing mode variation between replicates. Consistently, the highest number of unique OTUs were identified in specimens kept at Oceanário for both *E. verrucosa* (28.7%, 409 OTUs) and *P. grayi* (15.8%, 245 OTUs) (Supplementary Figure S10).

PERMANOVA revealed significant differences in microbial community composition among sampling locations for both *E. verrucosa* (PERMANOVA, *F* = 7.884, Df = 3, *p* < 0.01) and *P. cf. grayi* (PERMANOVA, *F* = 5.195, Df = 3, *p* < 0.01, Figure 3D; Supplementary Table S24). This compositional variation was visually supported by PCoA, which showed a clear separation of samples by location (Figure 3D). Analysis of homogeneity of group dispersions (PERMDISP) indicated significant differences in dispersion among sampling locations for *E. verrucosa* (PERMDISP, *F* = 15.037, Df = 3, *p* < 0.001) and *P. cf. grayi* (PERMDISP, *F* = 7.881, Df = 3, *p* < 0.01; Figure 3D; Supplementary Table S24).

#### 3.2.2 Captivity affects the microbial community at OTU-and family level

##### 3.2.2.1 Effect of short-term captivity on the microbiome of *Eunicella verrucosa* and *Paramuricea* cf. *grayi*

**Figure 4:**
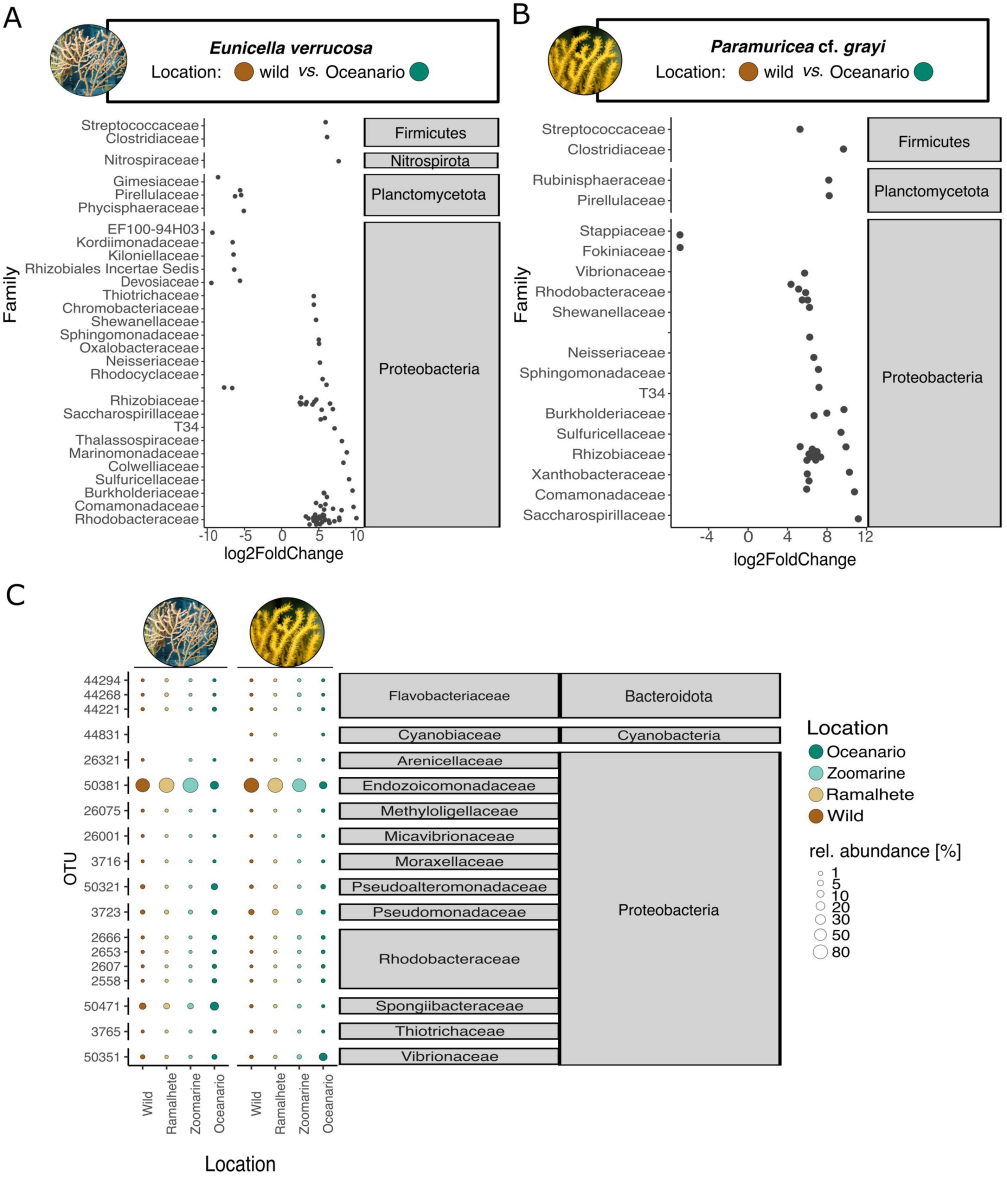
Microbe-level differences within *Eunicella verrucosa* and *P.* cf. *grayi* among locations [wild (Sagres), Ramalhete (day 45), Zoomarine (3 months), Oceanário (1 year)]. DESeq 2 analysis revealed significant changes in microbial dominance between the wild samples (control variable) and samples from Oceanário in **(A)** *E. verrucosa* and **(B)** in *P.* cf. *grayi*. Log2 fold change values are represented by black circles and the analyzed location groups (wild and Ocenário) within the DeSeq2 analysis are presented with their assigned color. Gray boxes give microbial taxonomic affiliation. **(C)** Relative abundances (in percent) of the 6 most abundant OTUs per coral species (total of 18 OTUs among all coral locations). Abundances are indicated by the bubble size and the sampling locations are displayed in color (values in Supplementary Table S18). Gray boxes give microbial taxonomic affiliation.

While there was no observable change in species composition and abundance of the most abundant microbes in *E. verrucosa* and *P.* cf. *grayi* after 45 days of aquarium keeping (Supplementary Figure S11), DESeq2 analysis revealed a significant decrease in the relative abundance of microbes in *E. verrucosa* (Supplementary Figure S12). Compared to the natural samples of *E. verrucosa*, a significantly reduced presence was identified for microbes of the phyla Bacteroidota (Flavobacteriacea, Cyclobacteriaceae), Myxococcota (Nannocystaceae), Planctomycetota (Gimesiaceae, Pirellulaceae), and Proteobacteria (EF100-94H03, Rhizobiales Incertae Sedis) (Supplementary Figure S12). In contrast, *Endozoicomonas* (OTU 50381) showed an increased abundance. No significant changes in the abundance were found between the wild sample of *P. grayi* and aquarium samples after 45 days (DESeq2). Comparing alpha and beta diversity of the aquarium water revealed that its microbiome was not significantly affected over 45 days, and there was also no (OTU-level) correlation with changes in the coral microbiome of *E. verrucosa* (Mantel test, r = 0.1001, *p* > 0.05) or *P.* cf. *grayi* (Mantel test, r = −0.1614, *p* > 0.05). Indicator value analysis identified microbes diagnostic for different sampling days: day 0 of *E. verrucosa* and sampling day 4 and 45 of *P.* cf. *grayi*. Samples of *E. verrucosa* on day 0 were characterized by OTUs affiliated with the families Cryomorphaceae (Bacteroidota), Arenicellaceae, Sphingomonadaceae (Proteobacteria) and one unclassified microbe within the phyla Proteobacteria (Supplementary Figure S13). After 4 days of aquarium keeping, indicator microbes identified in *P.* cf. *grayi* were assigned to the families Amoebophilaceae, 37–13 (Bacteroidota), and Rhodobacteraceae (Proteobactreia) and after 45 days, indicator microbes were classified as Rhizobiales Incertae Sedis and Xanthobacteraceae (Proteobacteria) (Supplementary Figure S13). Compared to the natural core microbiome of *E. verrucosa* (Figure 4A), six core OTUs were lost after 45 days of aquarium keeping [Devosiaceae (OTU 26064), Rhizobiaceae (OTU 26091), Cyclobacteriaceae (OTU 44098), Flavobacteriaceae OTU (OTU 44194) and one unclassified Proteobacteria (OTU 46449)] (Supplementary Figure S14). Despite this, Endozoicomonadaceae remained stable with 100% prevalence across detection thresholds of 0.1 to 2.5%. In contrast, Spongiibacteraceae, initially with 100% prevalence (0.1–2.5% prevalence), decreased to 70% prevalence at higher detection thresholds (<0.5% prevalence) (Supplementary Figure S14). Due to no significant changes in alpha, beta diversity and species composition the results for the core microbiome of *P.* cf. *grayi* after 45 days of aquarium keeping are only provided in Supplementary Figure S15.

##### 3.2.2.2 Effect of long–term captivity in different aquarium systems on the microbiome of *Eunicella verrucosa* and *Paramuricea* cf. *grayi*

**Figure 5:**
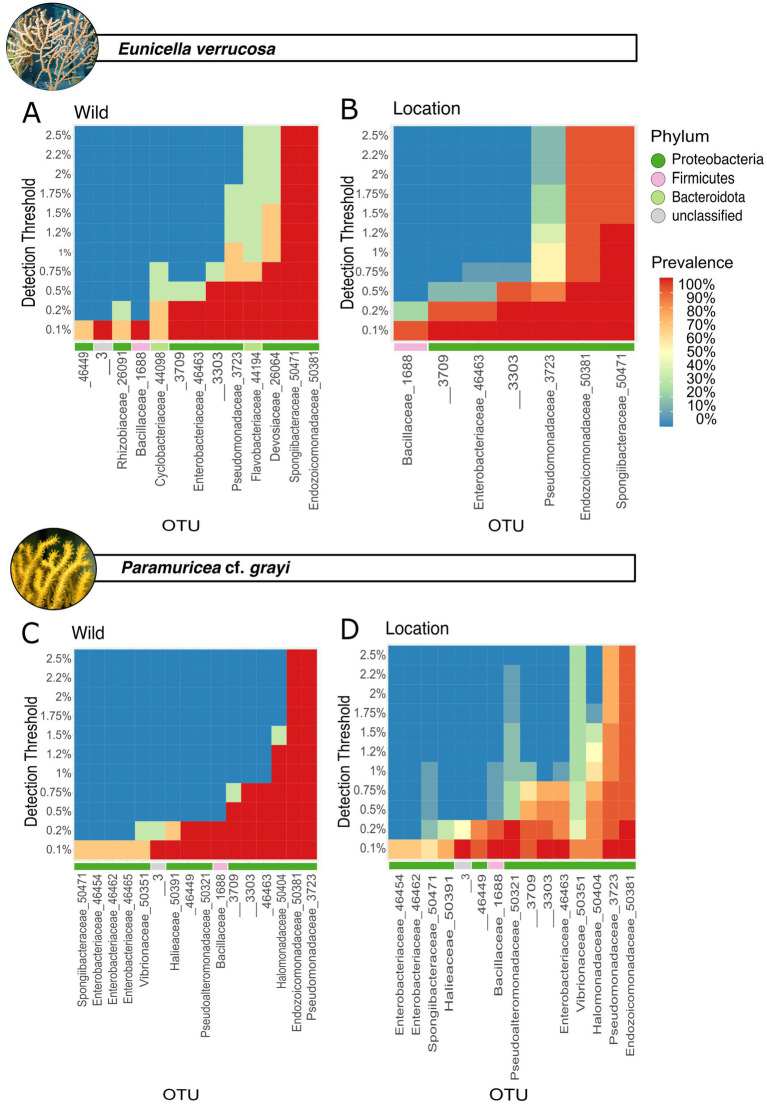
Core microbes differed between species and under aquarium conditions. For *Eunicella verrucosa*, the natural core microbiome is shown in **(A)** and the core microbiome of all *E. verrucosa* samples from different locations is shown in **(B)**. The natural core microbes of *P.* cf. *grayi* is shown in **(C)** and the core microbiome of all samples from different locations is shown in **(D)**. Prevalence (in percent) for the detection threshold (y-axis) is indicated by a color gradient of each. Microbial phyla are shown in color.

While there were no differences in relative abundances and the composition of dominant microbes among the natural samples and the *ex*-*situ* samples after 45 days in Ramalhete and Zoomarine, the samples from Oceanário showed a distinct microbial composition (Figure 5C). The relative abundance of Endozoicomonadaceae (OTU 50381) was much lower in Oceanário samples of *E. verrucosa* (16.44 ± 24.51%, Figure 5C; Supplementary Table S26) and *P.* cf*. grayi* (12.1 ± 6.61%, Figure 5C; Supplementary Table S26), compared to the natural samples, as well as to specimens kept at Ramalhete and Zoomarine (*E. verrucosa:* 83.93 ± 10.76% and *P.* cf. *grayi:* 79.58 ± 11.65%, Figure 5C; Supplementary Table S26). *Eunicella verrucosa* samples from Oceanário showed a ~ three-fold increase of microbes belonging to the family Spongiibacteraceae (17.94 ± 7.37%) compared to all other sampling locations (5.68 ± 1.05%), while *P.* cf. *grayi* from Oceanário showed a strong increase of Vibrionaceae (17.72 ± 17.89%) compared to the other locations (0.52 ± 0.35%, Figure 5C; Supplementary Table S26). In Oceanário samples DeSeq2 analysis further identified a significant increase in abundance covering 20 families in *E. verrucosa* and 17 families in *P.* cf. *grayi*, mostly belonging to the phylum Proteobacteria (Figures 5A,B). However, a decreased abundance in Oceanário samples was detected for 13 OTUs in *E. verrucosa* and two microbes, classified within the families Stappiaceae and Fokiniaceae, in *P.* cf. *grayi* (Figures 5A,B).

Indicator value analysis identified significant indicator microbes for the wild and Oceanário samples of *E. verrucosa* and *P.* cf. *grayi,* as well as for samples of *P.* cf. *grayi* from Zoomarine (Supplementary Figure S16). In total, 183 microbes were identified as diagnostic of *E. verrucosa* held in Oceanário (families Comamonadaceae, Rhodobacteraceae, Shewanellaceae, and T34), whereas 29 microbes belonging to the families Comamonadaceae, Xanthobacteraceae, and Rhizobiaceae (Proteobacteria), as well as Clostridiaceae (Firmicutes) were indicators for *P. grayi* kept in Oceanário (Supplementary Figure S16). In *E. verrucosa*, the core microbe Endozoicomonadaceae showed a reduced prevalence (90%) with a detection threshold < 0.5% under aquarium conditions, compared to the 100% prevalence under a detection threshold from 0.5–2.5% in the natural samples (Figures 4A,C). Under aquarium conditions, it was the Spongiibacteraceae that showed the highest prevalence (100% prevalence, 0.5–1.2% detection threshold, Figure 4C). In *P.* cf. *grayi* kept under aquarium conditions, the dominant core microbes, including Endozoicomonadaceae (OTU 50381), Pseudomonadaceae (OTU 3723), and Halomonadaceae (50404), remained dominant with lower prevalence (>80% prevalence up to 0.5% detection threshold, Figures 4A,C), while Vibrionaceae (OTU 50351) increased in prevalence to 90% at 0.1% detection threshold and 80% at 0.1% in aquarium conditions, compared to the 60% at 0.1% detection threshold in natural samples (Figures 4B,D).

## 4 Discussion

In this study, we unveiled the natural microbiome of 13 coral garden species occurring in SW Portugal (NE Atlantic) that we describe here as reference for future conservation and restoration work. Host species and order were found to significantly shape the coral microbiome diversity and composition. We also demonstrated the short- and long-term species-specific responses of the microbiome under captivity in two locally dominant gorgonian host species (*E. verrucosa* and *P.* cf. *grayi*) that are frequently used for maintenance in aquaria systems and are therefore of exceptional interest for restoration programs involving aquarium maintenance.

### 4.1 Host-specificity of microbiome across different taxonomic levels

Coral microbiomes have gained increasing interest over the last decades, and a high microbial host specificity has been frequently shown for both tropical shallow-water corals (Rosenberg et al., 2007; Ainsworth et al., 2015; Glasl et al., 2017), as well as for scleractinian cold-water corals (Galand et al., 2020; Appah et al., 2021). However, so far only a few representatives of coral garden forming corals were studied in detail and microbial records are available (Ransome et al., 2014; Van De Water et al., 2018; Keller-Costa et al., 2021). Our study aimed to contribute to a baseline for such temperate coral microbiomes.

We investigated *E. verrucosa*, *L. sarmentosa*, *P.* cf. *grayi, I. elongata*, and *A. subpinnata*, whose microbiome has been studied before in the Atlantic and Mediterranean (La Rivière et al., 2013; Ransome et al., 2014; Keller-Costa et al., 2017; Van De Water et al., 2018). The diverse coral community around Cape St. Vincent (Sagres, Portugal) comprises octocoral, scleractinian, and black coral species (Dias et al., 2020). The findings presented here not only expand the current microbiome dataset for coral garden-forming species but also deliver the first microbiome insights for previously undocumented species, shedding new light on the microbial diversity and host specificity in this ecologically important coral community. While this study provides the first broad assessment of the natural microbiome of coral garden species in the NE Atlantic, it is important to acknowledge limitations related to the sampling approach (via fisheries bycatch). While only visually healthy specimens were sampled, poor health conditions prior to collection may have introduced biases that affect the scalability of our conclusions. Nonetheless, host-specificity was observed across multiple coral taxa and consistently among biological replicates. Few examples of host specificity exist at the anthozoan order level, and our results reveal previously unreported marked differences in the microbiomes of different anthozoan orders (Figure 2). In particular, we identified a distinct microbiome associated with the Malacalcyonacea coral species studied (except *Alcyonium* sp. represented by one replicate), which displayed significantly lower alpha diversity and a unique microbial community composition compared to the other orders studied (Figure 2). We hypothesize that this relatively low variation and alpha diversity among Malacalcyonacea microbiomes could be linked to a homogeneous fan-like colony shape of the Malacalcyonacea species, and thus to low variation in the microbial niches made available by the host. Within this order, *Alcyonium* sp., which was only represented by one replicate, possessed a distinct microbiome from the fan-shaped representatives, which could be attributed to its different anatomical structure, and to the partially unresolved place of the genus within the Malacalcyonacea group which requires further revision (McFadden et al., 2022). However, given that this observation is based on a single specimen, it must be interpreted with caution. Limited replication prevents general conclusions, and further sampling will be required to verify whether this microbiome divergence is consistent across *Alcyonium* populations. Several factors, such as morphology and metabolic needs, can influence microbial host species-specificity by selecting for different microbial communities (McCauley et al., 2016; Camp et al., 2020). Further to coral morphology, depth-related environmental conditions (e.g., temperature, light availability, pressure) were not recorded in this study, but likely contributed to the observed species-specific microbiome variability, as microbial communities are known to vary along environmental gradients (Ransome et al., 2014; Keller-Costa et al., 2021; Reigel and Hellberg, 2023). Therefore, it remains unresolved if the observed microbiome specificity at the order level in Malacalcyonacea is driven by the inclusion of morphologically similar fan-shaped species, environmental conditions, or rather a true taxonomic signal, thus highlighting the importance of considering morphology and ecological traits in microbiome studies (McCauley et al., 2016; Camp et al., 2020). Microbial niches are potentially driven by the development of morphological microhabitats through increased body plan complexity [see McCauley et al., 2023 for a review], such as the development of skeletons in groups like Antipatharia, Helioporacea, and Scleractinia, and this process is suggested to result in increased microbial richness and diversity. Scleractinian corals are known to exhibit a strongly partitioned niche-specific microbiome in their mucus, tissue and skeletons (Rohwer et al., 2002; Rosenberg et al., 2007; van Oppen and Blackall, 2019), with higher microbial diversity in their mucus and skeleton, compared to their tissue (Marchioro et al., 2020; McCauley et al., 2023). In scleractinian corals, the skeletal endolithic community contributes to nitrogen and carbon fixation (Sangsawang et al., 2017; van Oppen and Blackall, 2019), whereas the mucus layer provides pathogen defense by providing a boundary niche for microbial commensals (Glasl et al., 2016; Marchioro et al., 2020). The coral tissue hosts stable, species-specific microbial communities (Rohwer et al., 2002; Glasl et al., 2016; Marchioro et al., 2020; Bergman et al., 2022). A similar case of high niche partitioning has not yet been observed in non-scleractinian coral species. However, distinct structural features are also well described within octocorals (McFadden et al., 2022). The potential presence of specific microbial communities within octocoral skeletal elements, such as calcitic sclerites or proteinaceous, and calcium carbonate axes in Malacalcyonacea and Scleralcyonacea (McFadden et al., 2022), akin to the endolithic communities found in scleractinian corals, remains largely unexplored. Similarly, whether the mucus-associated microbial community in octocoral species performs similar functions as those observed in tropical shallow-water scleractinian corals (Rohwer et al., 2002; Rosenberg et al., 2007; van Oppen and Blackall, 2019) remains to be determined. However, we found that coral garden species with high mucus production, specifically the octocorals *I.* cf. *elongata*, *P. rubra*, and the scleractinian *Dendrophyllia* spp., exhibited a high abundance of Rhodobacteraceae, a microbial group commonly associated with mucus production (Hall-Spencer et al., 2007; Frade et al., 2020; Marchioro et al., 2020; Keller-Costa et al., 2021). Since our study was conducted on homogenized coral fragments, the presence of multiple microhabitats with distinct microbial communities (Engelen et al., 2018) should be further addressed for coral garden species.

### 4.2 *In-situ* variation in microbial families and their potential roles

The gammaproteobacterial family Endozoicomonadaceae has been consistently reported as a dominant microbial component in tropical soft and hard corals (Ainsworth et al., 2015; Bourne et al., 2016; Pollock et al., 2018; Frade et al., 2020; Reigel and Hellberg, 2023), as well as in shallow-water populations of several temperate coral species (Bayer et al., 2013; La Rivière et al., 2013; Ransome et al., 2014; Keller-Costa et al., 2022). This study further confirms the consistently high abundance and prevalence of Endozoicomonadaceae across coral garden species (Figure 2; Supplementary Figure S8). The microbial family Endozoicomonadaceae is metabolically versatile, presumably contributing to coral holobiont health through vitamin translocation and chitin degradation, with recent research highlighting their chitinolytic activity as a key mechanism for nutrient acquisition in corals (Da Silva et al., 2023; McCauley et al., 2023).

In tropical photosymbiotic shallow-water corals, Endozoicomonadaceae have been frequently found in close proximity to the coral’s photosynthetic dinoflagellate endosymbionts (family Symbiodiniaceae). While dinoflagellates of corals produce Dimethylsulphoniopropionate (DMSP), and are thereby expected to provide carbon and sulphur to bacterial symbionts like Endozoicomonadaceae (Yost and Mitchelmore, 2009; Bourne et al., 2013), a recent study has shown that aphotic environments also contribute to the global DMSP production (Zheng et al., 2020). Here, we found microbes that metabolize DMSP, such as Endozoicomonadaceae, Alteromonadaceae, and Pseudomonadaceae (Bourne et al., 2013), to be potential core microbes in coral garden forming corals, similar to tropical shallow-water corals (Bourne et al., 2016; Frade et al., 2020; Haydon et al., 2022). Our data strongly supports the dominance of Endozoicomonadaceae within non-photosymbiotic coral garden species, such as *A. subpinnata, C. verticillata, F. quadrangularis,* and *I.* cf*. elongata,* reinforcing their importance beyond shallow-water symbiotic coral systems. In particular, microbiome observations for *Alcyonium* sp., *A. subpinnata*, and *C. verticillata* are based on a single sample (*n* = 1), which limits the generalizability of these findings and only indicate potential microbial patterns, which require further scientific support. However, the high dominance of Endozoicomonadaceae align with previous research outlining their association with anthozoans that lack photosymbionts, including a significant proportion of non-scleractinian corals (McCauley et al., 2023). The high presence of these microbial groups in coral garden species (Figure 2; Supplementary Figure S7) is most likely linked to their metabolic and immune-protective role for the coral host (Neave et al., 2016; Pogoreutz et al., 2022). Yet, future research needs to show if the production of DMSP can also be found in non-photosymbiotic corals and their associated microbes, similar to photosymbiotic corals (Raina et al., 2010; Frade et al., 2016). Juvenile apo-symbiotic corals can indeed produce DMSP, implying that non-photosymbiotic corals, including those in deep-sea environments, may have the capacity to produce DMSP independently of photosynthetic symbionts (Raina et al., 2010), offering therefore a potential explanation for the high abundance of Endozoicomonadaceae in the coral species we studied. This could give insights not only into the role of coral gardens in the context of climate regulation, but also into the role of DMSP under stress conditions for non-photosymbiotic corals.

Besides the association between Endozoicomonadaceae and photosymbiosis, these microbes have been generally linked to corals in shallow-water environments (Morrow et al., 2012; Bayer et al., 2013; Apprill et al., 2016; Neave et al., 2016; Frade et al., 2020) and were reported to be rare or absent in deep-sea corals (Kellogg, 2019; Appah et al., 2021). Here, we demonstrate that Endozoicomonadaceae, particularly OTU 50381 (*Endozoicomonas*), was the most abundant and dominant microbe in specimens of coral species from the order Malacalcyonacea predominantly sampled at depths ranging 60–80 m (e.g., *L. sarmentosa* and *Paramuricea* sp.) and 80–100 m (e.g., *Alcyonium* sp. (*n* = 1), *Eunicella verrucosa*, *L. sarmentosa*, and *P.* cf. *grayi*), but much less abundant in species occurring at greater depths of 100–400 (e.g., *C. verticillata* (*n* = 1), *E. paraplexauroides*, *I.* cf. *elongata*, *F. quadrangularis*, *P. rubra*, and *A. subpinnata* (*n* = 1), Figure 1; Supplementary Figure S4). Lower pH and temperatures found in deep-sea environments may limit the growth of Endozoicomonaceae (Kellogg, 2019). Our results indicate a dominance of Endozoicomonadaceae in corals sampled down to ~100 m depth and suggest that the prevalence of Endozoicomonadaceae may be linked to their ecological role in shallower coral habitats, underscoring their potential importance in supporting coral holobiont health within these environments.

In the present study, the microbiome of *P. rubra*, *I.* cf*. elongata,* and *F. quadrangularis* (Scleralcyonacea) showed a substantially high presence of Spongiibacteraceae (Figure 2; Supplementary Figure S7). This group is known to degrade hydrocarbons that probably facilitate energy acquisition and thus provides additional energy resources for corals in deep nutrient-poor environments (Quintanilla et al., 2022). Interestingly, Spongiibacteraceae also displayed increased abundance in species occurring in shallower-water within the Malacalcyonacea family, particularly in species such as *Eunicella verrucosa* and *Leptogorgia sarmentosa* (Cúrdia et al., 2013). Rhodobacteraceae and Flavobacteriaceae, which are often observed in response to elevated nutrient levels (Frade et al., 2020; Keller-Costa et al., 2021), were also found to be highly abundant, prevalent, as well as indicative of coral garden species (Figures 2, 5; Supplementary Figure S7). These microbes are also associated with amino acid biosynthesis, nitrogen fixation, and sulfur metabolism in healthy corals (Hall-Spencer et al., 2007; Connelly et al., 2023). Contradicting to the presence of Spongiibacteraceae, which support corals in nutrient poor environments, this high presence of Rhodobacteraceae and Flavobacteriaceae may be linked to elevated nutrient levels associated with the nutrient-rich upwelling region around Cape St. Vincent in SW Portugal, the submarine canyon (Haynes and Barton, 1990; Relvas and Barton, 2002; Nestorowicz et al., 2021), and the predominant hydrology (Relvas and Barton, 2002; Rautenbach et al., 2024). Altogether, these nutrient-associated microbes could reflect natural variability in nutrient availability, as well as adaptive responses to environmental conditions, with potential links to terrestrial runoff and estuarine input in the region.

Our study has also identified Vibrionaceae as a predominant and persistent member of the core coral microbiome (Figure 2; Supplementary Figure S7). Vibrionaceae are frequently identified as coral pathogens and linked to disease outbreaks in tropical and temperate shallow-water corals and mortality events (Hall-Spencer et al., 2007; Glasl et al., 2017; Frade et al., 2020; Keller-Costa et al., 2021; Connelly et al., 2023), and have been observed to increase in abundance under stressful conditions and during disease onset in temperate corals (Hall-Spencer et al., 2007; Corinaldesi et al., 2022). However, recent research indicates a more complex role. For example, Keller-Costa et al. (2021) found no significant increase in *Vibrio* abundance in the microbiomes of necrotic *Eunicella gazella* tissue compared to healthy tissue. This suggests that the presence of Vibrionaceae in our study, particularly OTU 50351, may simply reflect a natural component of the coral microbiome rather than an indication of pathological conditions. In contrast to other temperate coral species studied to date, in which the family Spirochaetaceae is usually found as a regular member (Van De Water et al., 2016, 2017; Keller-Costa et al., 2022; McCauley et al., 2023), we did not find this group to be part of the core microbiome of the species analyzed, with one exception. One OTU from this family (OTU 46622, Spirochaetaceae) was abundant in *Alcyonium* sp. (*n* = 1) (Malacalyconaceae) and in some members of Scleralcyonacea [*C. verticillata* (*n* = 1), *P. rubra*, *I.* cf. *elongata*]. The identified abundance of Spirochaetaceae in *Alcyonium* sp. (Malacalcyonacea) and *C. verticillata* (Scleralcyonacea) only serves as an indication for a high occurrence in these species, due to the limited replicate number (*n* = 1). Spirochaetaceae are generally associated with healthy octocorals, support nutrient cycling and development, and have been suggested to even influence the color of the precious red coral *Corallium rubrum* (Van De Water et al., 2016; Van De Water et al., 2018; Bonacolta et al., 2021; McCauley et al., 2023). However, despite the high abundance in Scleralcyonacea the precise functions of Spirochaetaceae in the coral microbiome remain unclear and require further investigation. Pseudomonadaceae emerged as the second most prevalent and abundant microbial group in our study (Figure 2; Supplementary Figure S7). Pseudomonadales, including *Pseudoalteromonas*, have frequently been associated with healthy corals and are known for their role in the biosynthesis of amino acids and B vitamins (Keller-Costa et al., 2022; McCauley et al., 2023) and were found to be abundant in the coral tissue, skeleton, and mucus, highlighting their functional importance within and across different coral compartments (Keller-Costa et al., 2022; McCauley et al., 2023). Although we did not analyze specific microhabitats within the corals sampled, our findings provide a basic understanding of the microbial composition in coral garden species and highlight the inclusion of Pseudoalteromonadaceae as an integral component that should be further investigated in future research.

### 4.3 Short-term microbial responses to aquarium conditions: distinguishing microbiome conformers and regulators

A recurring “rule of thumb” is that aquarium conditions induce a rapid decline in alpha diversity within tropical coral holobionts but diversity often recovers upon reintroduction to the natural environments (Vega Thurber et al., 2009; Glasl et al., 2016). Our study revealed that despite the generally homogeneous microbiome of the Malacalcyonacean studied, *E. verrucosa* and *P.* cf. *grayi* exhibit distinct microbial responses under similar *ex-situ* conditions. We observed that *E. verrucosa* exhibited rapid microbial changes, particularly within the first 12 h and continuing over 45 days. This response of *E. verrucosa* aligns with the concept of “microbiome conformers” (Ziegler et al., 2019; Van De Water et al., 2020), which are species with a high degree of microbial flexibility, as opposed to “microbiome regulators” (Ziegler et al., 2019; Van De Water et al., 2020), which maintain a more stable holobiont structure and composition, as was observed for *P.* cf. *grayi* in our study. However, the distinction between microbial conformers and microbial regulators appears to be a continuum, with coral species displaying varying degrees of microbial regulation or conformity (Keller-Costa et al., 2021). Our results align with previous studies showing that *E. verrucosa* exhibits an immediate microbial response to stress, a reaction similarly observed in other temperate coral species such as *L. sarmentosa* (Ransome et al., 2014; Keller-Costa et al., 2022). It is also possible that the specific conditions under which the corals are maintained, including the stressors they encounter, may better suit some species than others, resulting in the different responses shown by *P.* cf. *grayi* and *E. verrucosa*. While *P. cf. grayi* showed greater microbiome stability under short-term captivity, the physiological basis for this remains unclear, as no coral health metrics or stress indicators were collected in this study. Future research combining microbiome data with host physiology could help elucidate mechanisms of resilience.

Closely related species like *Eunicella cavolini* have been identified as microbiome regulators due to their low microbial variation under temporally and spatially changing environmental conditions (Van De Water et al., 2020). Differences in the microbial responsiveness within the genus *Eunicella*, might be attributed to the geographic distribution of *E. cavolini*, extending from the Mediterranean Sea into the Atlantic (Ransome et al., 2014; Aurelle et al., 2017), which may have driven the development of a more flexible microbiome in *E. cavolini*, compared to *E. verrucosa* which shows a geographically wide distribution within the Atlantic. Future studies are needed to conduct a distribution wide comparative analysis on species within the *Eunicella* genus and across other coral garden taxa, specifically to identify microbial response patterns to changing conditions to develop targeted restoration and conservation approaches for coral garden forming species.

The observed significant decrease in alpha diversity of *E. verrucosa* kept over 45 days in the aquarium compared to wild-collected samples could be linked to a lower regulative capacity of the host, resulting in a higher transmissibility of microbes. Yet, microbial changes in *E. verrucosa* occurred independently of the water community, which aligns with earlier studies emphasizing the different microbial composition of corals compared to the surrounding water communities (Ransome et al., 2014; Glasl et al., 2016). While the transient nature of microbiomes is presumably beneficial to enable the host to rapidly adapt to dynamic environments (Frade et al., 2020), severe environmental stress can weaken the host’s immune defense, and pathogens and opportunistic microbes can invade the coral and proliferate, ultimately leading to the formation of a pathobiome (Stévenne et al., 2021; Voolstra et al., 2024). Fast acclimation processes take place in rare prokaryotes, while significant community-level shifts in core microbial communities tend to occur after long-term environmental changes (Ransome et al., 2014; Hernandez-Agreda et al., 2016; Haydon et al., 2022; Reigel and Hellberg, 2023). Here, we also found that core microbes remained stable in *E. verrucosa* and *P.* cf. *grayi* over short-term aquarium maintenance (Figure 5).

As microbial regulators or conformers, corals possess different ways to maintain a beneficial host microbiome. One mechanism is periodic mucus shedding (Glasl et al., 2016). Another way to control and/or prevent a microbial shift can be through biochemical pathways that favor certain groups of microbes (Raina et al., 2013; Jones and King, 2015; Ochsenkühn et al., 2018). For instance, photosymbiotic scleractinian corals can expel Symbiodiniaceae symbionts under stress or adjust their symbiont composition by switching to more temperature- and light-resistant strains (Teplitski et al., 2016; LaJeunesse et al., 2018; Voolstra et al., 2024). The existence of similar mechanisms in non-photosymbiotic corals to “switch” to beneficial microbes and to selectively expel unwanted microbes (Teplitski et al., 2016) remain largely unexplored. However, “microbial switching” with exchanged OTUs within the same bacterial genus has recently been documented in other gorgonian species under stressed or altered conditions, both in symbiotic and non-symbiotic taxa (Van De Water et al., 2018; McCauley et al., 2020; Montaño-Salazar et al., 2023), supporting the observation that *ex-situ* maintenance can lead to significant holobiont restructuring. Drivers for distinct microbial changes in the two corals *E. verrucosa* and *P.* cf. *grayi* (and corals in general), whether mediated molecularly, behaviourally or by immunity, need to be further investigated in order to improve our understanding of the different levels of active and passive microbiome alterations in corals.

### 4.4 Long-term microbial dynamics in corals under aquarium maintenance: microbial shifts in alpha and beta diversity and microbiome composition

In contrast to the typical short-term responses of the microbial conformer *E. verrucosa* and the microbial regulator *P.* cf. *grayi* in the different aquaria, both coral species exhibited significant microbial shifts with increased alpha and beta diversity and notable changes in microbial community composition after 1 year at the Oceanário. Galand et al. (2018) highlighted that coral colonies typically require 1 year to fully acclimate to aquarium conditions. Whether our findings are primarily due to the length of time in captivity, or are influenced by specific aquarium conditions, such as water quality, nutrition, and other factors, remains to be explored. The water quality and nutrient content was not controlled in this study, but by comparing microbial communities in this study we found that changes in the microbiome of *E. verrucosa* or *P.* cf. *grayi* did not correlate with the microbial community in the water samples, which concurs with earlier publications (Ransome et al., 2014; Glasl et al., 2016). Water samples from Oceanário displayed differences in the microbial composition (Supplementary Figure S17) and a distinct separation (PCoA, Supplementary Figure S18) between water samples, but due to limited sample size, the observed difference requires further investigation and statistical support. In particular, higher beta diversity (high dispersion among samples) and a distinct microbial community structure have often been associated with a reduced regulatory capacity of the host and thus often considered to be the result of a dysbiotic state in corals, typically reflecting stress or disease of the host (Thurber et al., 2017; Zaneveld et al., 2017; Keller-Costa et al., 2021; Voolstra et al., 2024). In fact, *E. verrucosa* and *P. grayi* showed a greater interspecific dissimilarity in the Oceanário samples than in the specimens kept at the Zoomarine public aquarium, as well as at Ramalhete and in the natural environment (Figure 3; Supplementary Figure S19). It has been shown that destabilization of the typical microbial community under stress is strongly associated with a decline in essential Gammaproteobacteria, such as Endozoicomonadaceae, which commonly characterize a healthy microbiome in corals (Ransome et al., 2014; Keller-Costa et al., 2021; Voolstra et al., 2024). Consistent with this, we observed a reduction in *Endozoicomonas* (OTU 50381) abundance in *E. verrucosa* and *P.* cf. *grayi* samples from Oceanário, potentially indicating a shift toward a dysbiotic state under these aquarium conditions for 1 year (Figures 4, 5C). Yet, this decline was not significant (Figure 5) and Oceanário has been recognized by its success in gorgonian long-term maintenance, where coral colonies consistently appeared healthy and in good condition. At the same time, we observed a rise in opportunistic microbes such as Rhodobacteraceae, Sphingomonadaceae, and Saccharospirillaceae, along with a significant increase in Vibrionaceae in *P. cf. grayi* samples at the Oceanário (Figures 4, 5C), which could indicate a dysbiotic state of the corals kept for 1 year in the aquarium. Other microbial groups in samples of *E. verrucosa* and *P.* cf. *grayi* at Oceanário, including Saccharospirillaceae, Rhodobacteraceae, and Sphingomonadaceae also showed an increased abundance (Figure 5). Saccharospirillaceae are known for their adaptability to fluctuating environmental conditions through aerobic and anaerobic respiration (Campbell et al., 2022). Rhodobacteraceae are associated with corals (La Rivière et al., 2013; Keller-Costa et al., 2021; Luo et al., 2021) and linked to dysbiosis under changing environmental conditions or coral disease in both temperate (Keller-Costa et al., 2021; Corinaldesi et al., 2022) and tropical regions (Frade et al., 2020). The increased presence of these families, particularly in relation to nutrient cycling (Appah et al., 2021), may reflect an enrichment of specific nutrients in the aquarium environment, potentially in response to feeding processes under human care, promoting the growth of opportunistic species and indicating a shift in the microbial community structure under different trophic conditions (Pogoreutz and Ziegler, 2024). Common aquarium maintenance practices, such as tank cleaning and water changes, as well as environmental parameters (light, temperature, flow) can lead to shifts in microbial assemblages, affecting nutrient cycling in aquarium systems, and thus affecting the reared organisms (Bik et al., 2019). However, not all environmental parameters (e.g., light intensity or spectrum) were controlled or recorded here, potentially impairing direct *ex-situ* comparisons. This especially applies for mesophotic species, as the observed microbiome shifts could reflect both the effects of captivity and mismatches in the naturally prevailing environmental parameters. Overall, while opportunistic microbes increased, the relatively stable presence and prevalence of core microbes with a potentially beneficial role, such as Endozoicomonadaceae and Pseudomonadaceae, may indicate that the pathogen defense role provided by these groups (Ransome et al., 2014; Keller-Costa et al., 2021) may still be maintained under long term aquarium conditions. Therefore, whether this microbial response of the coral colonies after 1 year at Oceanário is indicative of a dysbiotic state or an altered microbiome under human care needs further investigation.

Studies on tropical and cold-water coral species have shown that microbiomes respond rapidly and species-specifically to captivity, underlining the relevance of these responses for conservation efforts. For example, Barreto et al. (2021) emphasized the difficulty of simulating natural environmental conditions in captivity and the resulting implications for microbial community dynamics. Rapid shifts in the coral surface mucus layer microbiome were observed after 1 day in captivity in tropical species such as *Fungia granulosa* (Kooperman et al., 2007), *Porites astreoides* (Glasl et al., 2016), and *Siderastrea siderea* (Pratte et al., 2015). In contrast, microbiome stability has been observed in *Madrepora oculata*, which maintained a stable microbial community over 6 months (Galand et al., 2018), and in *Acropora loripes* over 4 weeks (Hill and Ralph, 2006; Damjanovic et al., 2020). Our findings align with these studies indicating species-specific differences in response to aquarium conditions: *Eunicella verrucosa* showed rapid microbiome shifts under short-term captivity, similar to *Lophelia pertusa* (Galand et al., 2018), while *Paramuricea cf. grayi* maintained greater microbiome stability. Our study supports recent findings on microbial taxa of potential interest for microbiome stewardship approaches (Peixoto et al., 2022), such as Endozoicomonadaceae, Pseudomonadaceae, and Spongiibacteraceae, yet their functional roles in coral garden species resilience remain to be further experimentally validated. Ongoing work is investigating whether coral garden species show post-reintroduction variation in survival after microbiome shifts under captivity, as demonstrated for tropical corals (Glasl et al., 2016), and will assess how microbial consortia aid restoration outcomes for coral garden species.

## 5 Conclusions and implications for restoration

Monitoring the natural microbiome is essential for understanding coral health and resilience, and our study provides a critical reference for the microbiomes of temperate coral garden species. By documenting microbial diversity, particularly for species not previously studied, this work contributes valuable baseline data for future research. Identifying responses to short- and long-term captivity, our study is crucial for guiding coral restoration and understanding microbial changes upon reintroduction to natural environments. Recent advances in tropical coral transplantation (Pandolfi et al., 2003; Boström-Einarsson et al., 2020; Corinaldesi et al., 2023) and the potential of microbiome-assisted restoration (Peixoto et al., 2019; Corinaldesi et al., 2023) underscore the importance of these approaches, which could contribute to the effective conservation and restoration success of both shallow-water tropical coral reefs and deep habitat-forming species in mesophotic to aphotic zones.

## Data Availability

Raw sequencing reads associated with this project are archived in the NCBI Sequence Read Archive (SRA) database (Project number PRJNA1284359, https://www.ncbi.nlm.nih.gov/bioproject/PRJNA1284359).
