# Supplementary material for: Coral garden conservation and restoration: how host taxon and ex-situ maintenance affect the microbiome of soft and hard corals
Source: Front Microbiol. 2025 Aug 6;16:1605105. doi: 10.3389/fmicb.2025.1605105 (PMC12364619; doi:10.3389/fmicb.2025.1605105)
Supplement: Supplementary file 1 [file Data_Sheet_1.pdf]

Supplementary Material

1 Supplementary Data

1.1 Supplementary Material and Methods

Sample Collection and Processing

Coloration of coral colonies. Many coral species display color variations, and *Eunicella verrucosa* exhibits both white-rose and salmon color variants. Due to limitations in availability, the white-rose color morph was sampled for approach 1) and in the two public aquaria, Zoomarine and Oceanario, for approach 2). However, both colormorphs were sampled over 45 days in the research station Ramalhete. The gorgonian *Paramuricea cf. grayi* (yellow lineage, sensu Coelho et al. 2023) and *Paramuricea sp.* (purple lineage, sensu Coelho et al. 2023) have been recently described as genetically distinct species with high genetic distance (Coelho et al. 2023). Therefore, both taxa were included in approach 1) to assess the natural microbiome, but only the yellow lineage (*P. cf. grayi*) was sampled for approach 2) under captivity.

1.2 Supplementary Results

Microbiome results on the water samples. We found that alpha diversity of the microbial community in the water did not significantly change under aquarium conditions within 45 sampling days (KW, Shannon: chi-squared test statistics = 11, Df = 11, $p > 0.05$; Observed: chi-squared test statistics = 11, Df = 11, $p > 0.05$; Chao1: chi-squared test statistics = 11, Df = 11, $p > 0.05$) (supplementary material, Tab. S14). The non-parametric Kruskal-Wallis test also revealed no significant difference in alpha diversity between locations (KW, Shannon: chi-squared test statistics = 4, Df = 4, $p > 0.05$; Observed: chi-squared test statistics = 4, Df = 4, $p > 0.05$; Chao1: chi-squared test statistics = 4, Df = 4, $p > 0.05$) (supplementary material, Tab. S14). Assessing significant differences in beta diversity using PERMANOVA was not applicable due to the small sample size of the water samples. Nevertheless, a separation of water samples taken from different locations was visualized using the ordination method PCoA, based on Bray-Curtis dissimilarity (supplementary material, Fig. S18). All four locations exhibited dissimilarities, while the two samples obtained from the two different coral aquarium (Oceanario_EV, Oceanario_PG) tanks in Oceanario displayed a low dissimilarity between samples. The water samples taken over 45 days displayed a transition from wider dissimilarity towards less dissimilarity between the samples (supplementary material, Fig. S18).

Relative abundances of the most abundant micorbes transitioned towards a more homogeneous amount of relative abundances among the most abundnat micorbes, as shown in Fig. S13 (supplementary material).The wild sample had higher abundances of certain microbes compared to aquarium samples. For example, one OTU from the family Cyanobiaceae (Cyanobacteria) was abundant in the wild sample, reappeared on day 8, and persisted after day 24. Most OTUs from the family Rhodobacteriaceae (Proteobacteria) were consistently abundant across all water samples, with some fluctuations over the 45-day period. Conducting a Mantel test using Bray-Curtis dissimilarities and Spearman's correlation (1000 permutations) we found, that changes in the microbial aquarium water communities did not correlate with changes in the corals. Results showed no significant correlation between microbial diversity changes in *E. verrucosa* ($r = 0.1001$, $p > 0.05$) or *P. grayi* ($r = -0.1614$, $p > 0.05$) and the water samples.

Comparing the relative abundance of the most abundant microbes computed for the gorgonians and the water from different aquaria, displayed different dominances of micorbes across the sampling locations. *E. verrucosa* and *P. grayi* were kept in separate aquaria at Oceanario, where water samples from both tanks showed similar microbial abundances with a homogeneous pattern of the relative abundances. In contrast, Zoomarine tank water had a distinctly higher abundance of one OTUs from the family Flavobacteriaceae. The wild water sampe was obtained from the harbor in Sagres and as listed above, showed a higher abundance of an OUT belonging to the family Cyanobiaceae and Moraxellaceae (supplementary material, Fig. S17).

1.3 Supplementary Discussion

Differences between Scleralcyonacea and Malacalcyonacea microbiomes. The microbial composition of the polyp tissue is profoundly influenced by feeding behaviors and the functional community present within the endoderm and gastrovascular cavity (Sweet, Croquer, and Bythell 2011). A high variation in colony structure and polyp morphology among Scleracyonacea species (McFadden, Van Ofwegen, and Quattrini 2022) are likely to drive significant differences in their microbiome composition compared to the more uniform Malacalcyonacea group. However, we observed that *Alcyonium*, a genus within the Malacalcyonacea group, exhibits a notably distinct microbiome, characterized by unique microbial community composition and beta diversity. This divergence in microbiome structure may be attributed to the complex and their lower understood taxonomy within the octocoral group (McFadden, Van Ofwegen, and Quattrini 2022). Specifically, *Alcyonium*'s colony morphology deviates from the fan-like shapes typical of other Malacalcyonacea members. The influence of morphological features on microbial community differences requires further investigation, and a comprehensive taxonomic revision of the *Alcyonium* genus is necessary to better understand the observed microbiome disparities.

Statistical Power of Natural vs. Aquarium Samples. Although the three replicates sampled from the natural colony in the field were not the same as those sampled over time in the aquarium, the inclusion of these replicates however provided statistical power to detect a trend towards a rapid effect on the microbiome of *E. verrucosa* to transport and aquarium conditions.

Impact of food sources on the microbial community. Specifically, the differences of the provided food sources, might be an important additional factor explaining differences between natural microbiome at the aquarium keeping, and also explaining the different responses in different aquarium systems, as increased nutrient levels have been shown to correlate with changes in the prokaryotic community (Garren and Azam 2012; Moura et al. 2016; Stévenne et al. 2021; Oduor, Cristina, and Costa 2023).

2 Supplementary Figures and Tables

2.1 Supplementary Figures

2.1.1 Figures Material and Methods

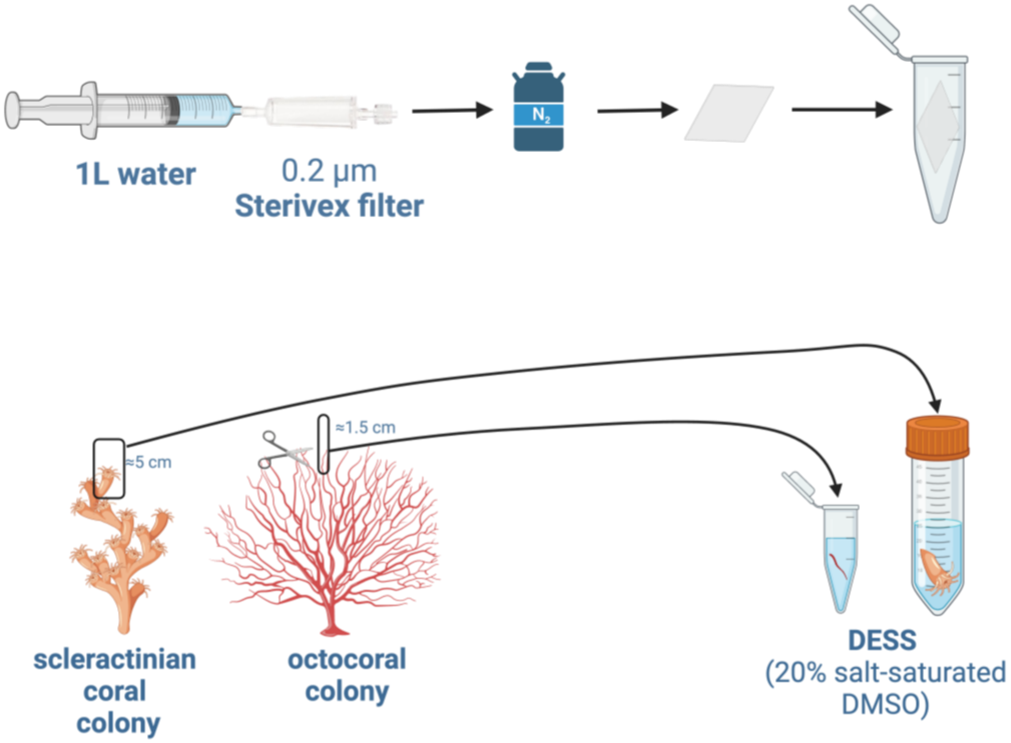

Fig. 1. Sampling procedure of the coral samples, collecting ≈1.5 cm of octocoral fragments and ≈ 5 cm of scleractinian corals, with subsequently fixating the samples in DESS (20% salt-saturated DMSO) together with water samples, which were obtained by filtering 1L of aquarium water through 0.2 µm Sterivexfilters (Merck Millipore) filters and flash-freezing of the samples using liquid nitrogen. Figure created in BioRender. Rola, M. (2025), https://BioRender.com/undefined.

2.1.2 Figures Results

2.1.2.1 *In-situ* microbiome

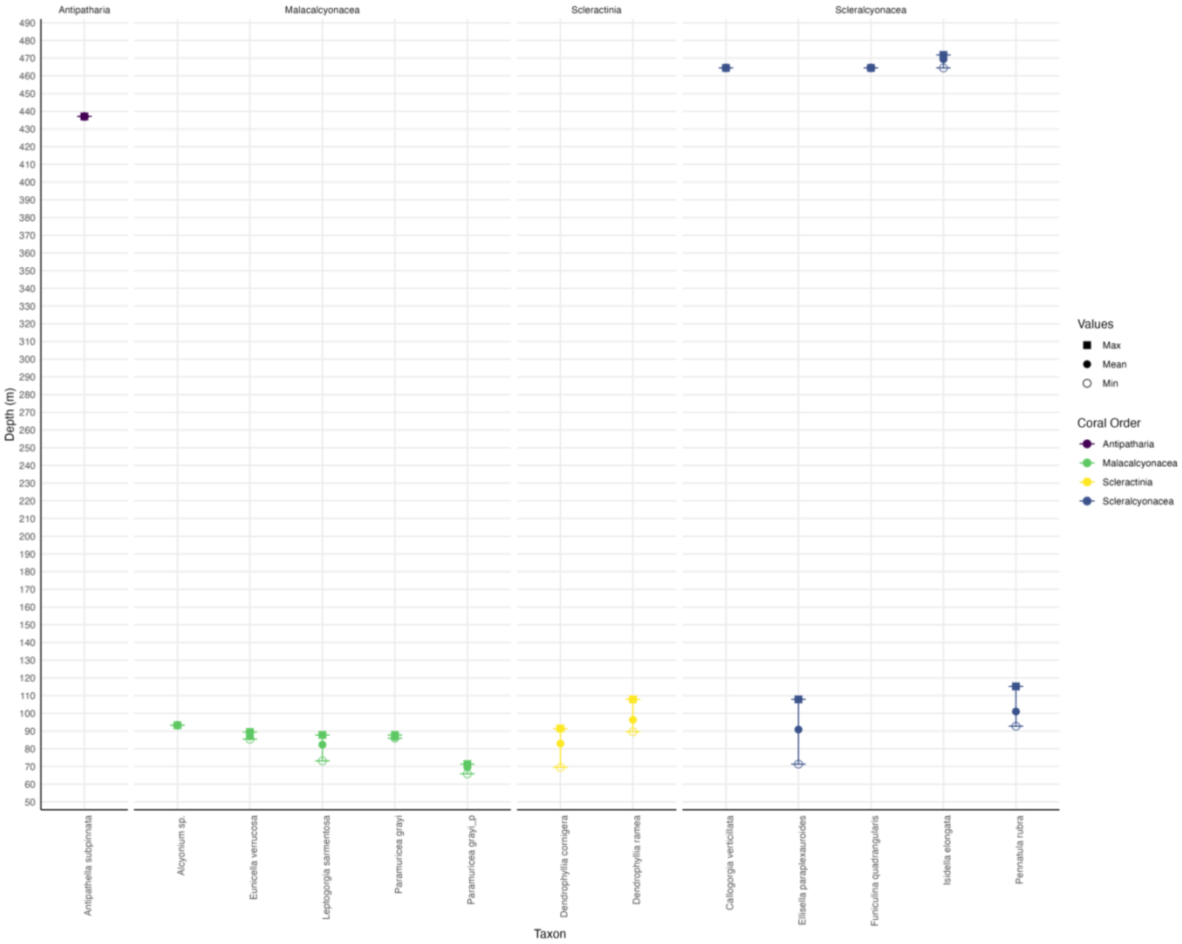

Fig. 2. Overview of the depth (in meters) recorded for the coral species collected in Sagres. Displayed are the minimum, maximum, and mean depth and the coral orders are indicated by color. Four depth groups, 60-80m, 80-100m, 100-120m, and 400-500m were defined for analysis based on the clustering of samples resulting from the recorded coordinates and their depth (in meters), respectively (shown in Fig.1).

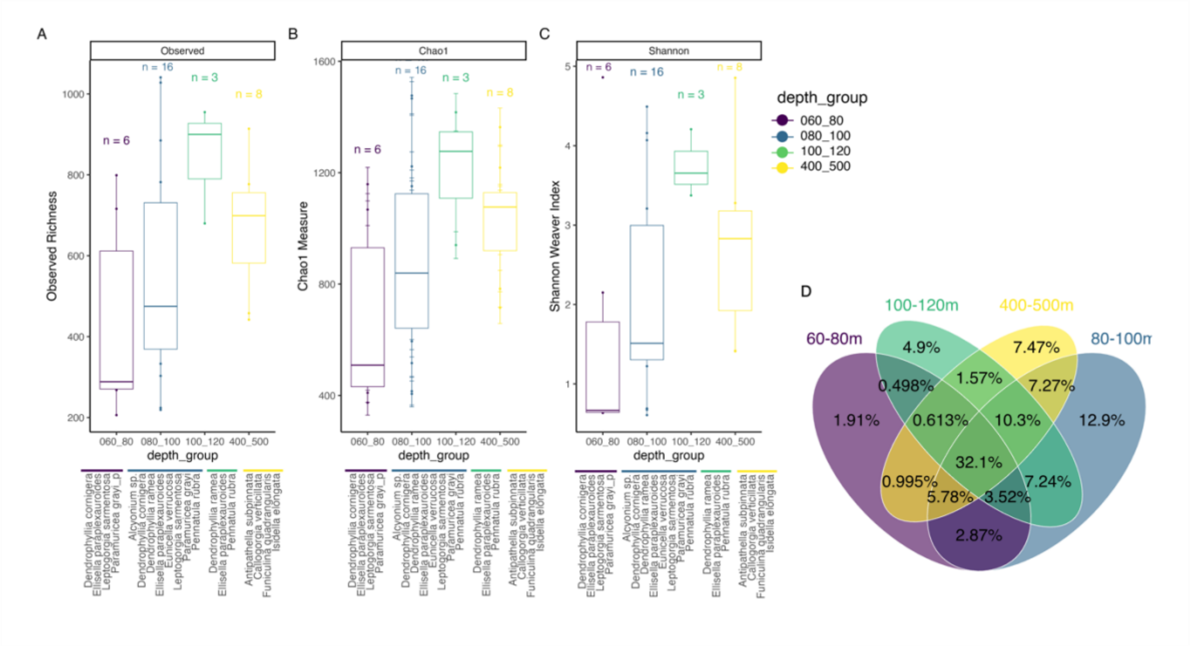

Fig. 3. Visualized are the four geographic depth sites 60-80m, 80-100m, 100-120m, and 400-500m, their alpha diversity, and number of shared, unique, and ubiquitous OTUs. In **A-C)** microbial alpha diversity Observed richness, estimated richness (Chao1), and Shannon Index of coral taxa samples from the four geographic depth sites are displayed. The computed significance of alpha diversity using ANOVA is indicated with an asterisk. *Paramuricea grayi*_p refers to *Paramuricea* sp. (the purple color morph) and *Paramuricea grayi* refers to *Paramuricea* cf. *grayi* (yellow color morph) **D)** Percentages of shared, unique, and ubiquitous OTUs between geographic depth groups are visualized in the Venn diagram. Alpha diversity results for the different location-depth sites, and in D) the venn diagram showing shared, ubiquitous and unique OTUs (in %).

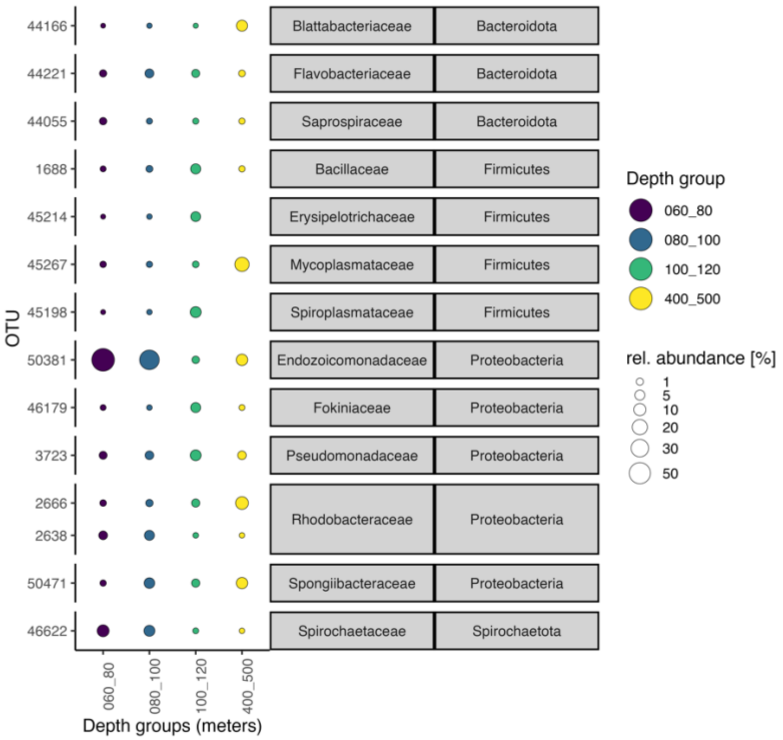

Fig. 4. Relative abundances of the most abundant OTUs for different geographic-depth groups and their comprised coral species. Visualized are the relative abundances (in %) of the 14 most abundant Operational Taxonomic Units (OTUs) across all geographic-depth groups. Included were the five most abundant OTUs per geographic-depth group, resulting in 14 highly abundant OTUs among all coral taxa. Coral taxa are displayed by color. Bubble sizes indicate the relative abundances (in %).

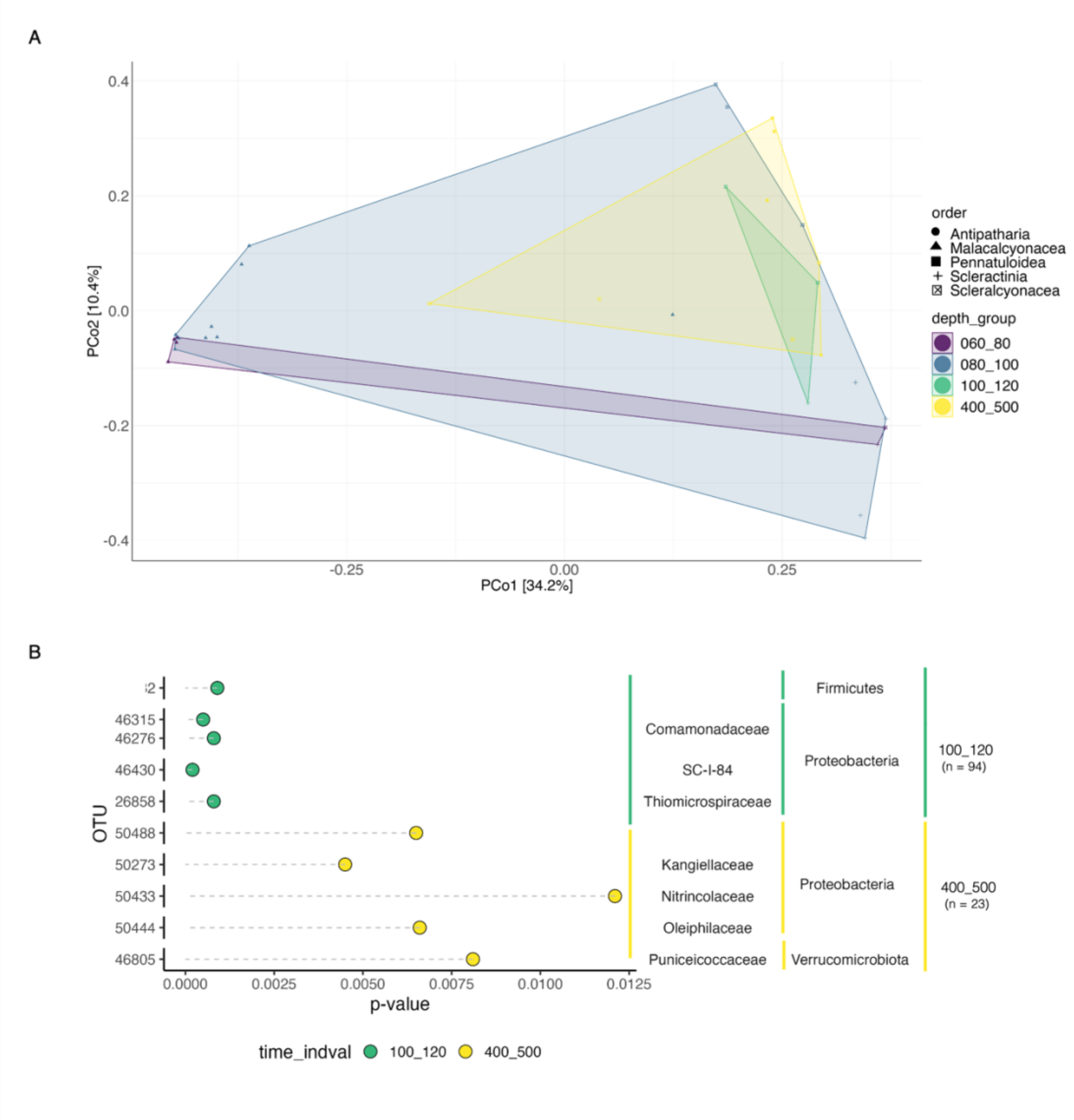

Fig. 5. Microbial community structure and identified indicator microbes for different geographic-depth groups and their comprised coral species. **A)** Principal coordinates analysis based on Bray Curtis dissimilarities (999 permutations). Depth groups are displayed by color and the coral orders are indicated by shapes. **B)** Indicator microbes were identified using the IndVal test. The five indicators with the highest significance values, if present, are presented. Colors show the different location depth-sites and the x-axis shows the p-value.

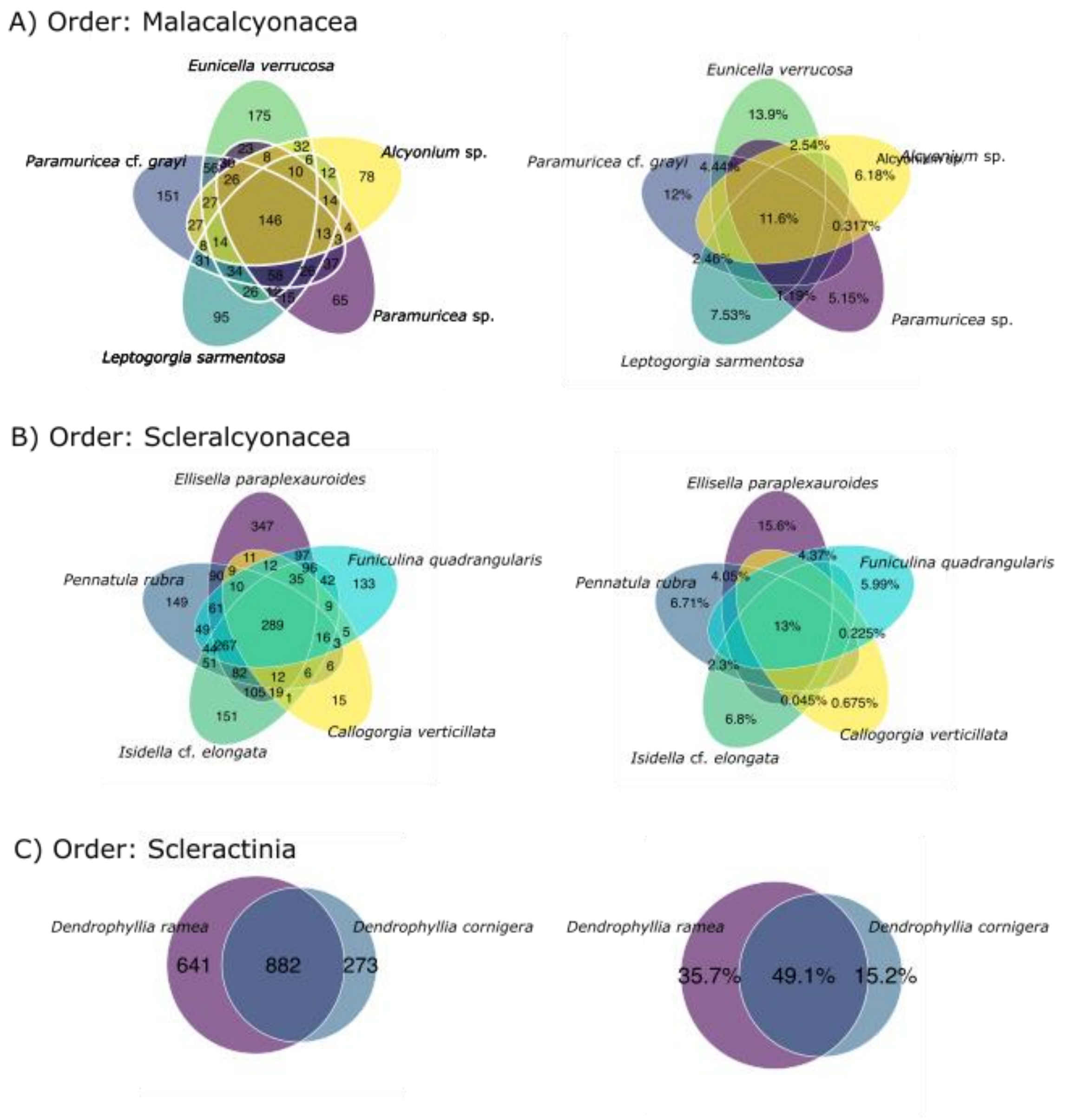

Fig. 6. Number of OTUs and the respective percentages (%) of shared, unique, and ubiquitous OTUs between the different coral order A) within the coral order Malacalcyonaceae, B) coral species within the octocoral order Scleralcyonaceae and C) the hexacoral order Scleractinia.

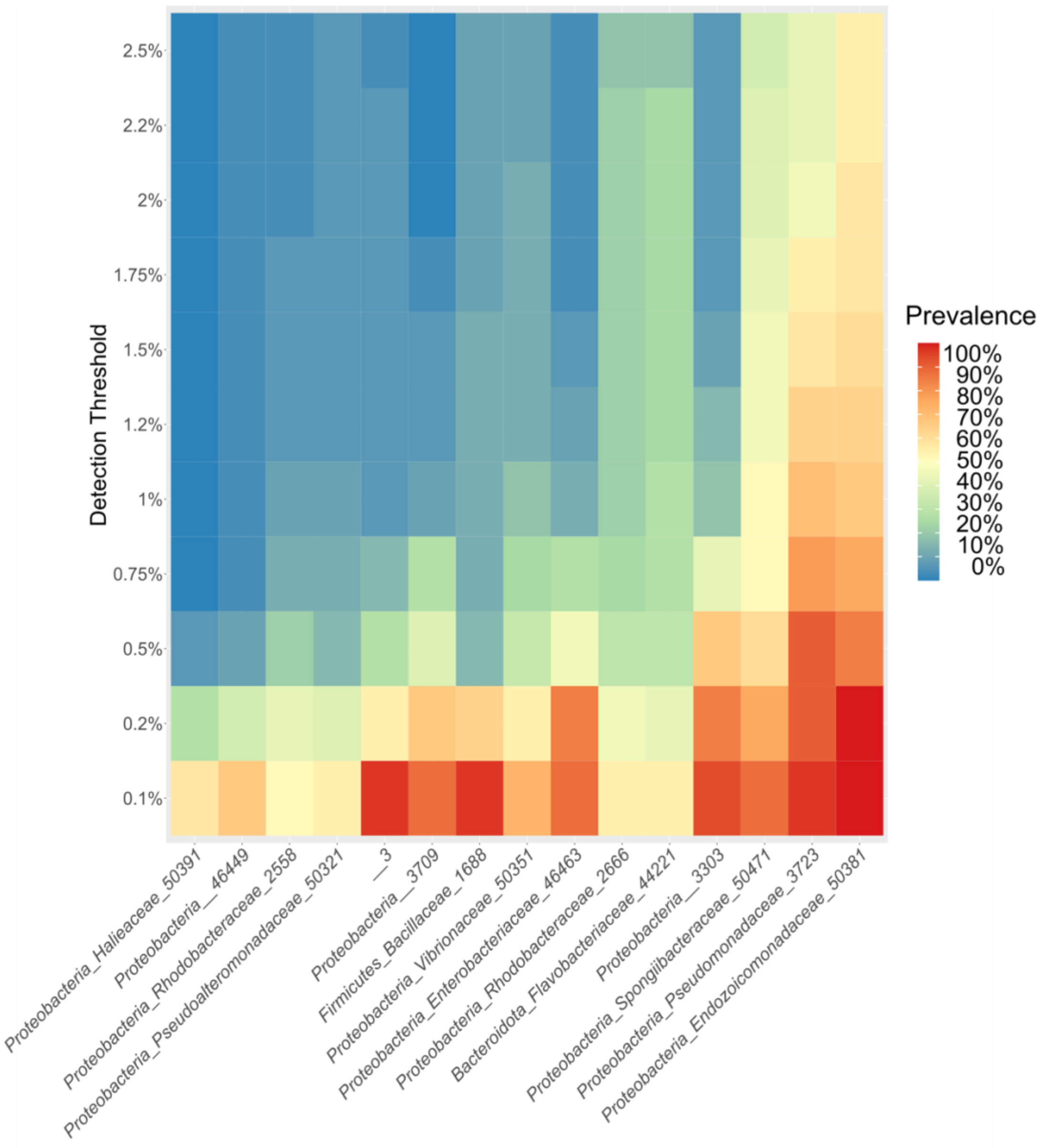

Fig. 7. The natural core microbes are shown for coral garden species (12 species), where all samples are pooled togethser. Prevalence (in percent) for the detection threshold (y-axis) is indicated by a colour gradient of each OTU across the samples.

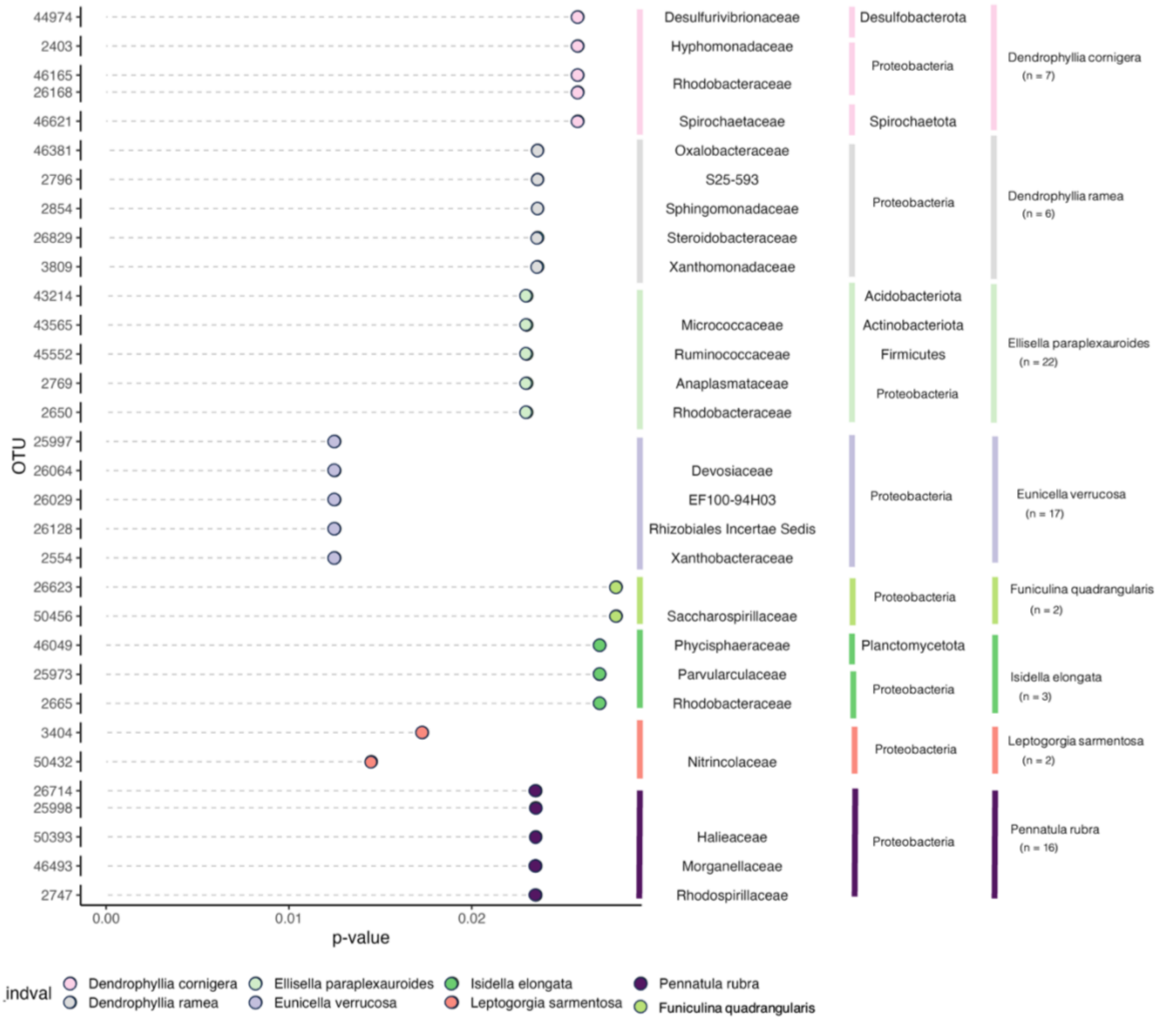

Fig. 8. Indicator microbes were identified using the IndVal test (indicator value analysis). The five indicators with the highest significance values, if present, are presented. Colors display the different coral garden species and the x-axis shows the p-vlaue of the detection of the indicator microbe. (Coral species colors are not displayed in the color-blind friendly virids palette due to the insufficient color depth for 13 coral species)

2.1.2.2 *Ex-situ* microbiome

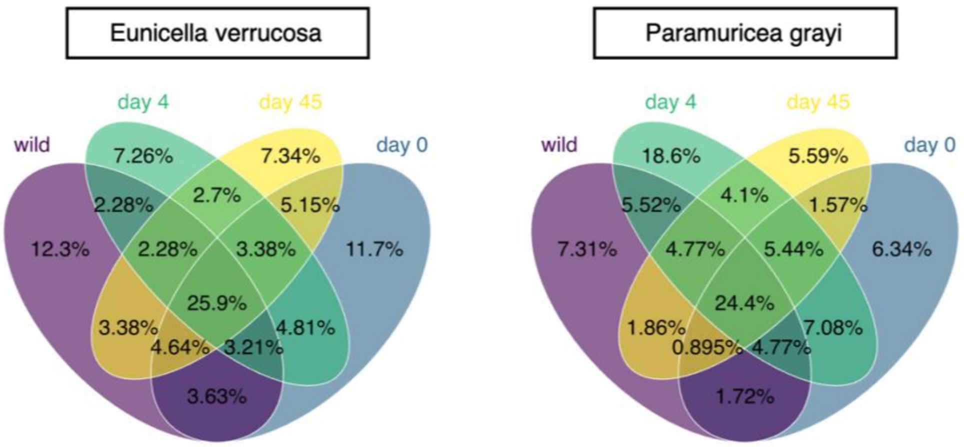

Fig. 9. Percentages and number of OTUs of shared, unique, and ubiquitous OTUs between the different sampling days: days 0, 4, and 45, including the wild sample for *E. verrucosa* and *P.* cf. *grayi* over-time (Ramalhete).

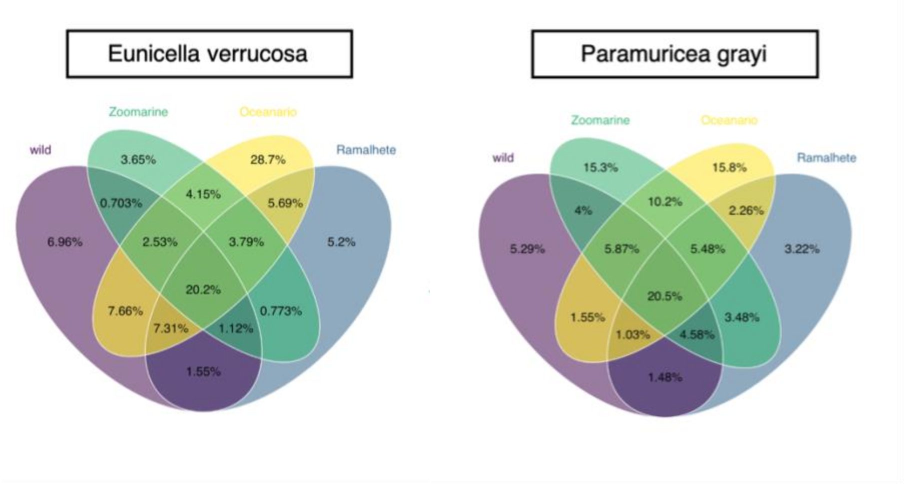

Fig. 10. Percentages and number of OTUs of shared, unique, and ubiquitous OTUs between the different sampling locations: wild (Sagres), Ramalhete (day 45), Zoomarine and Oceanario for *E. verrucosa* and *P.* cf. *grayi* over-time (Ramalhete).

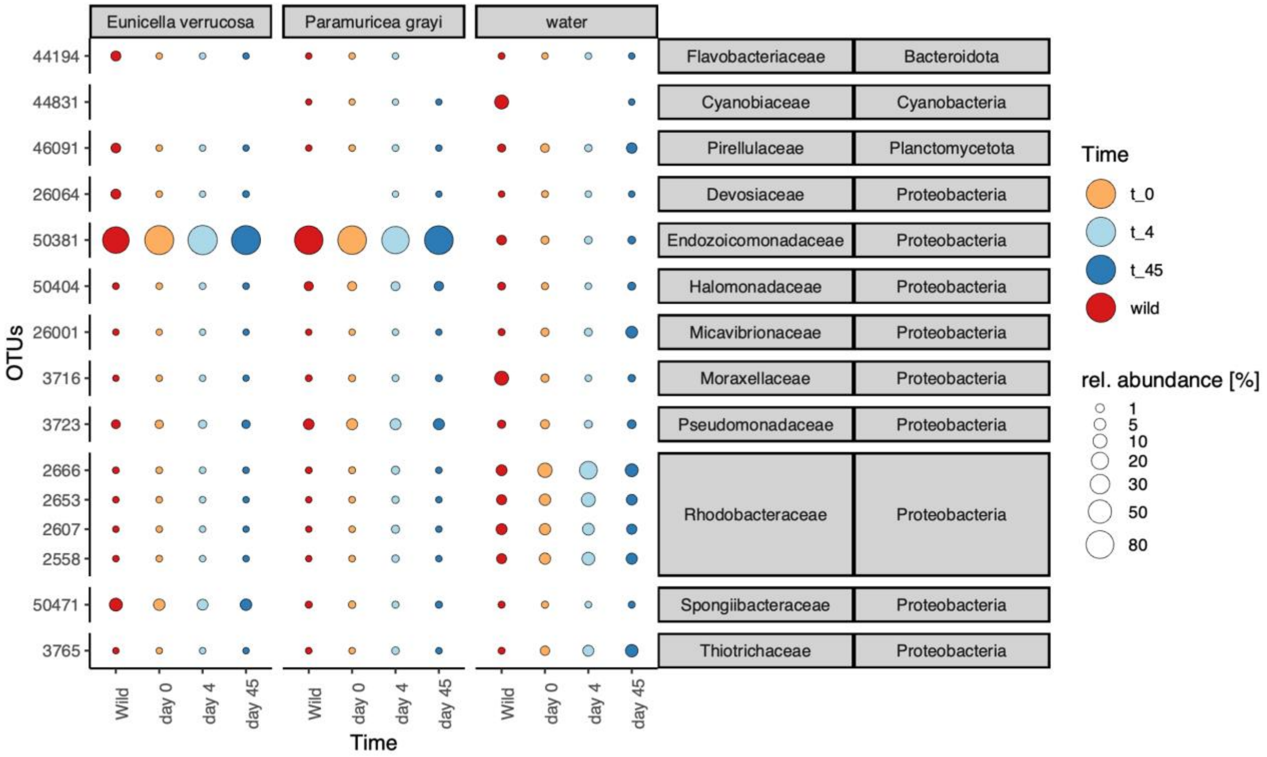

Fig. 11. Microbial community composition of *E. verrucosa* and *P.* cf. *grayi* kept in captivity over short-term (45 days), including aquarium water samples. The bubble plot involved the calculation of the 12 most abundant Operational Taxonomic Units (OTUs) per group (per taxon (coral, and water samples)), resulting in a total of 15 most abundant microbes across all sampling groups. To compare the most abundant microbes of the coral colonies with the water samples, the transport bucket water (wild), days 0, 4, and 45, were included in the bubble plot. Relative abundances of OTUs are shown in percentage, the circle sizes indicate the percentage and colors indicate the sampling times (day 0, 4, 45, and wild).

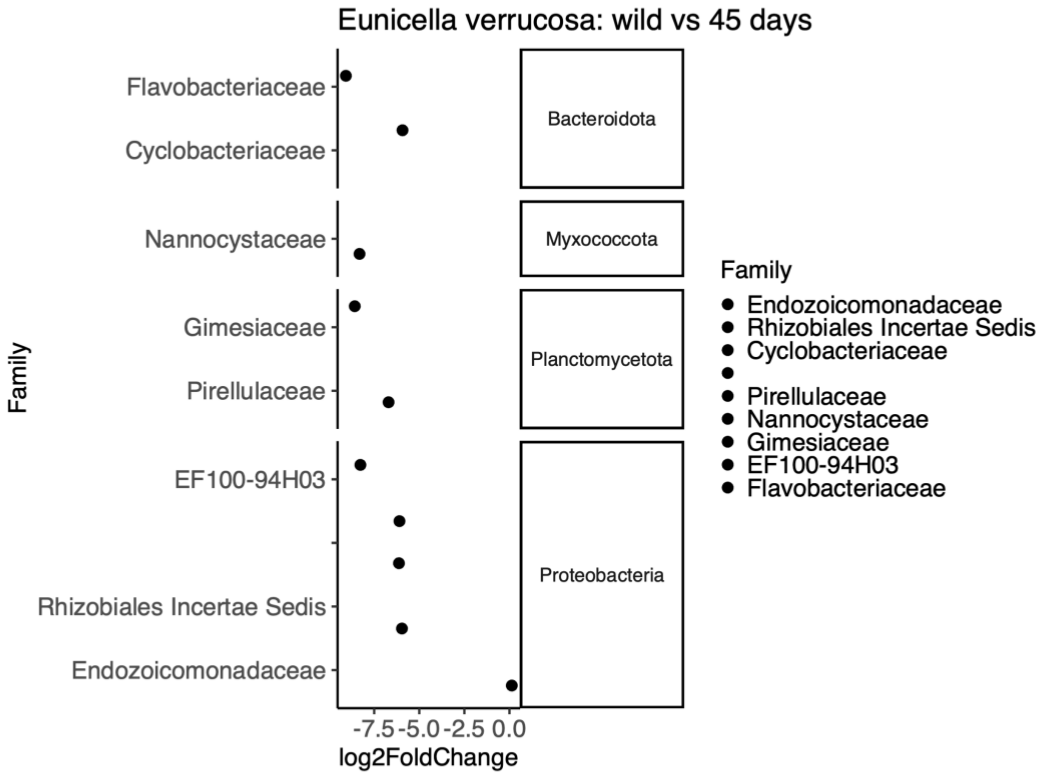

Fig. 12. Microbe-level differences within *E. verrucosa* over short-term (Ramalhete, over 45 days). DESeq 2 analysis revealed significant changes in microbial dominance between the wild samples (control variable) and samples from Oceanário de Lisboa.

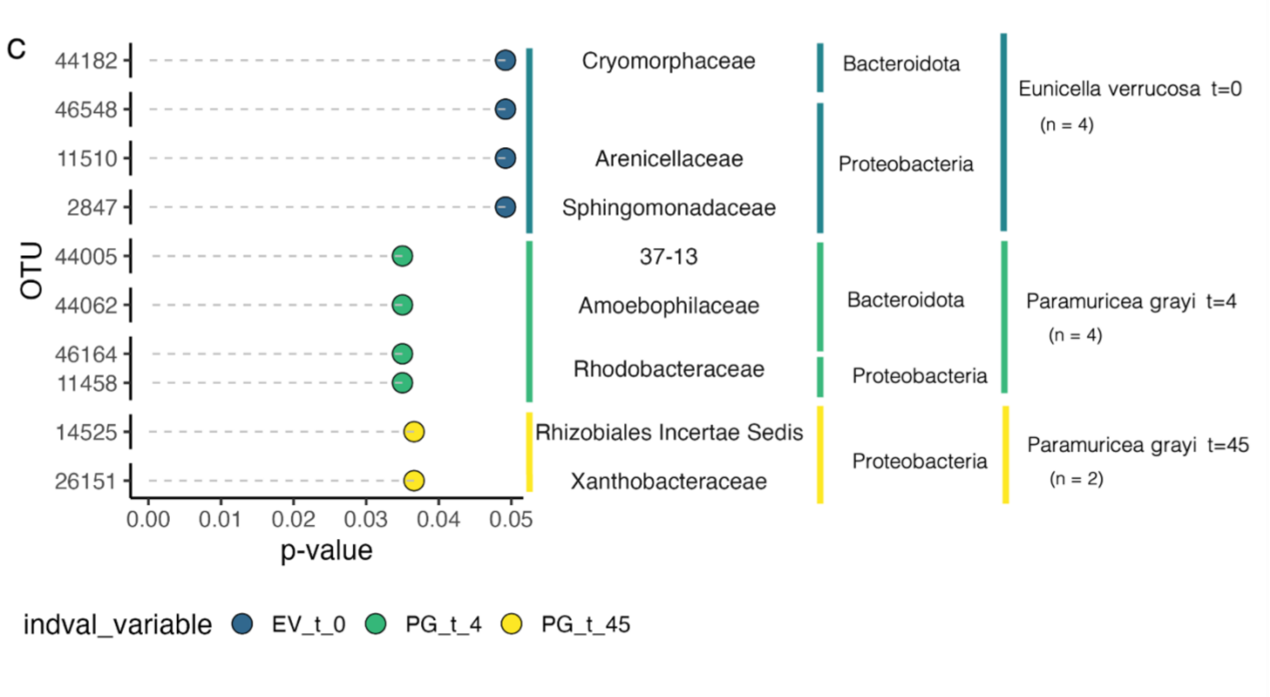

Fig. 13. Indicator microbes for *Eunicella verrucosa* and *Paramuricea* cf. *grayi* and their samples taken over time (wild, t = 0, 4, and 45). Indicator microbes were identified using the IndVal test. Colors indicate the coral species and the sampling day (species_t_day). The five indicators with the highest significance values, if present, are presented.

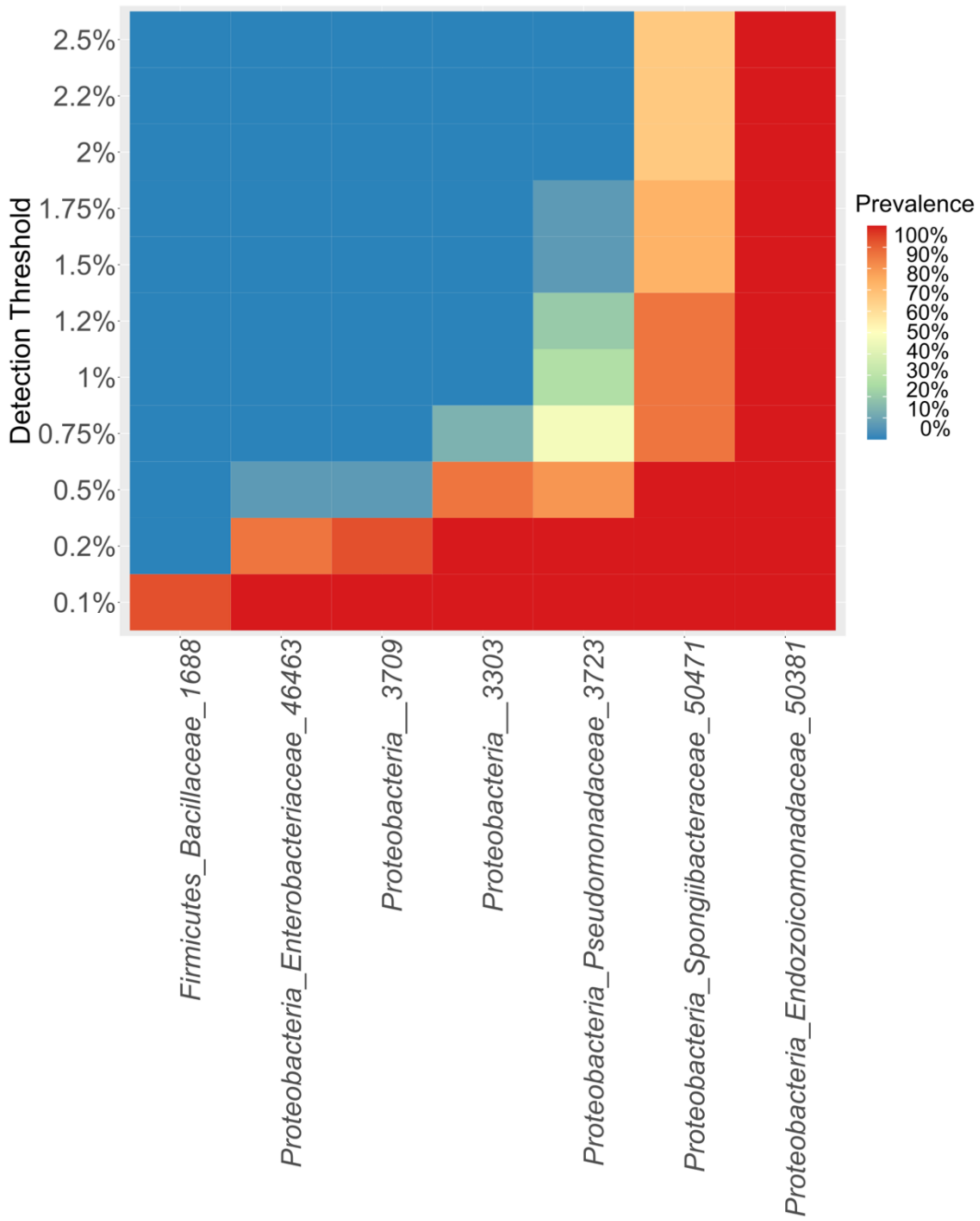

Fig. 14. Core microbiome of *Eunicella verrucosa* over short-term. Prevalence (in percent) for the detection threshold (y-axis) is indicated by a colour gradient of each OTU across the samples.

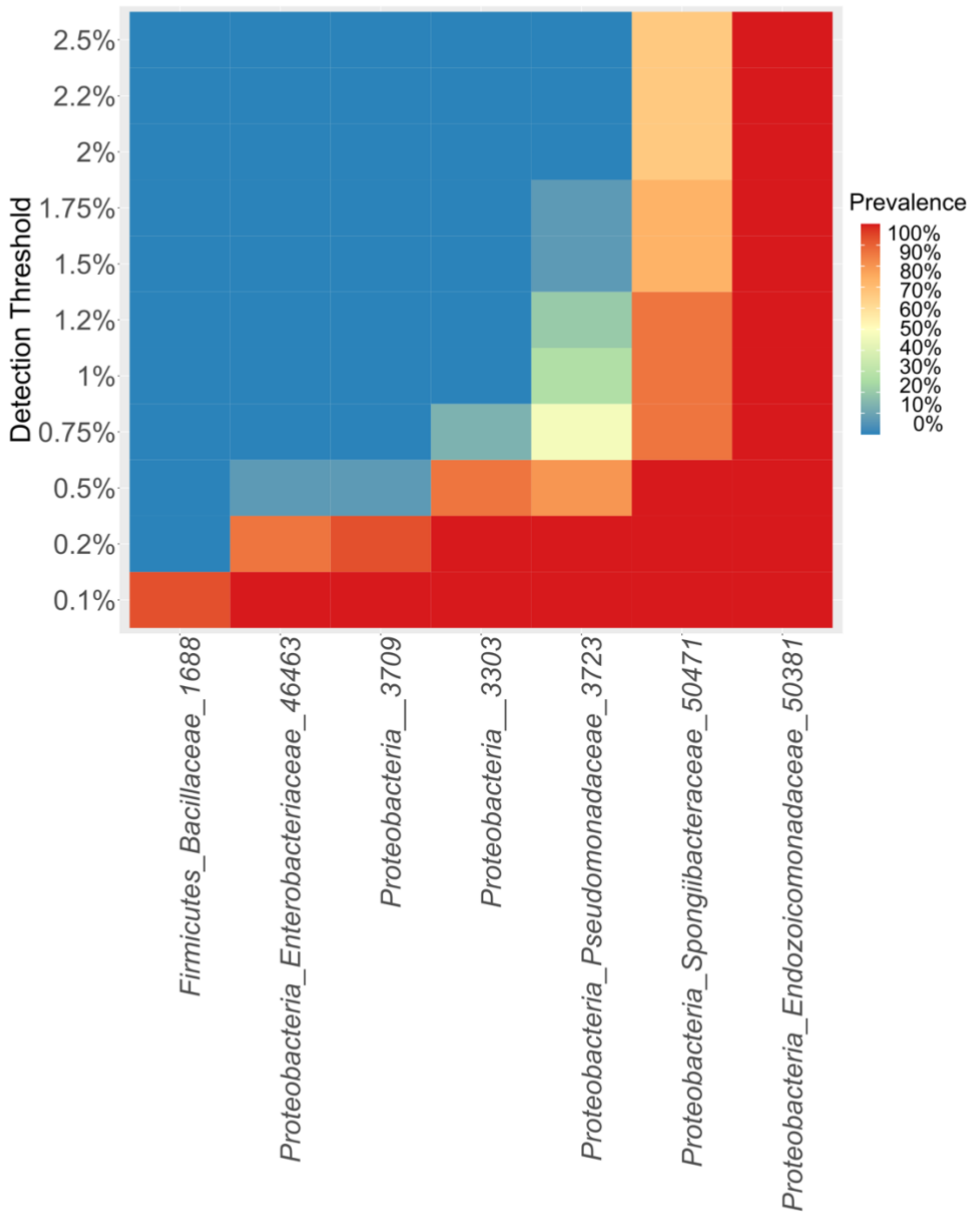

Fig. 15. Core microbiome of *Paramuricea* cf. *grayi* over short-term. Prevalence (in percent) for the detection threshold (y-axis) is indicated by a colour gradient of each OTU across the samples.

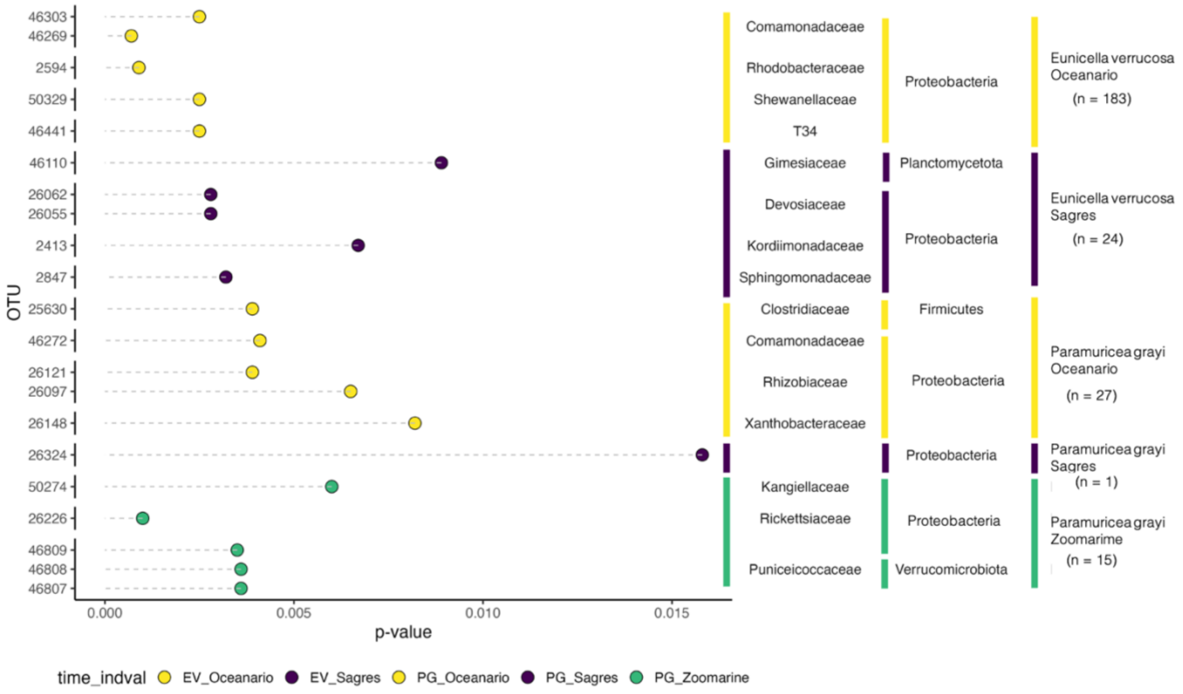

Fig. 16. Indicator value analysis (Indval) identified indicators for the different coral taxa, *Eunicella verrucosa*, and *Paramuricea* cf. *grayi*, sampled in Oceanario, Zoomarine, Sagres (wild), and on day 45 at the research station Ramalhete. Colors indicate the sampling location and Ix-axis shows the p-value.

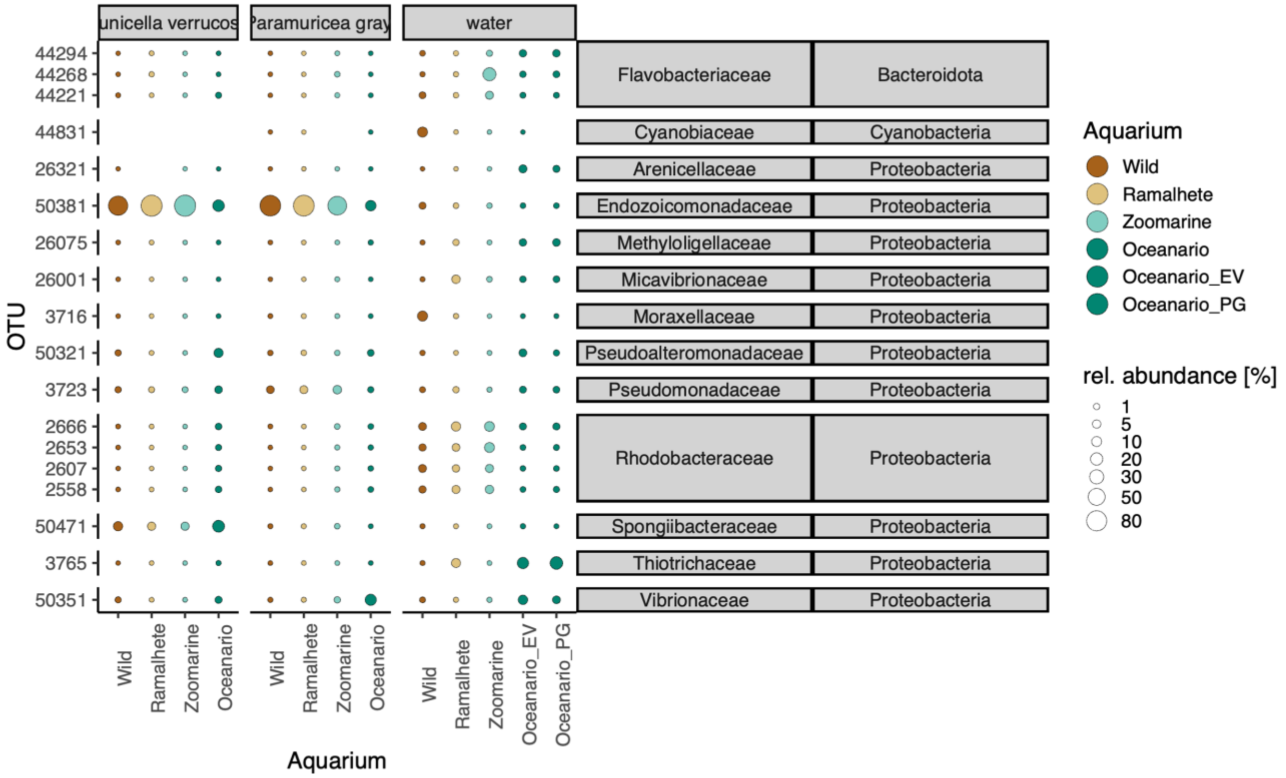

Fig. 17. Microbial community composition of *E. verrucosa*, *P*. cf. *grayi*, and water samples from the different sampling locations, including one wild sample are displayed. The assessment of microbial abundance per location involved the calculation of the 20 most abundant Operational Taxonomic Units (OTUs) per group (per taxon (coral, and water samples)), resulting in a total of 20 most

abundant microbes across all sampling groups per location. Relative abundances of OTUs are shown in percent (%), the circle sizes indicate the percent value and colors indicate the sampling locations.

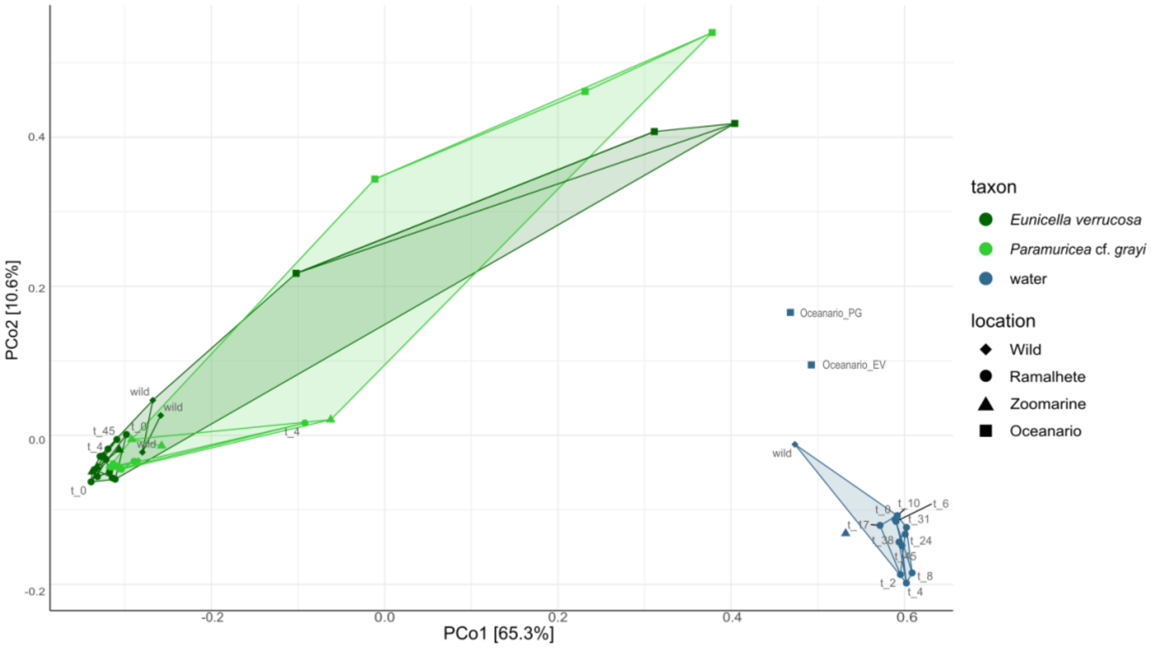

Fig. 18. Principal coordinates analysis based on Bray Curtis dissimilarities (999 permutations). Coral species are displayed by color and the sampling locationss are indicated by shapes. Coral and water samples taken over short-term are further displayed on a track using the sample_trajectory() function from ampvis2.

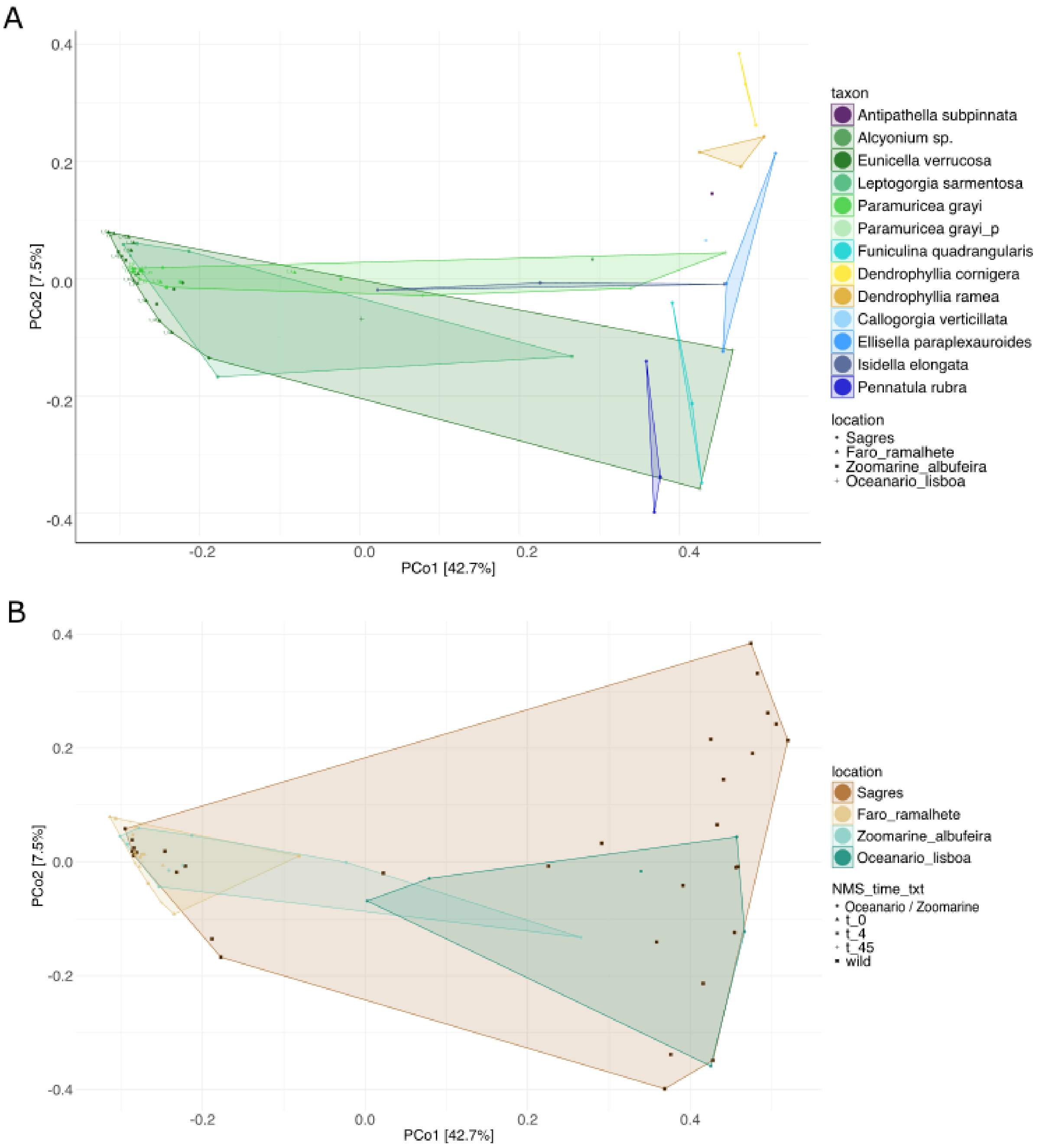

Fig. 19. PCoA based on Bray Curtis dissimilarities with 999 permutations shows group separations, with A) coral garden taxa being displayed by color and B) sampling locations being displayed by color and each data point representing one sample. This NMDS displays all samples of this study including objective 1) Natural Microbiome and 2) *ex-situ* microbiome with short-term and long-term objective.

2.2 Supplementary Tables

2.2.1 Statistics for 1) Natural Microbiome

Tab. 1. Alpha diversity metrices for all coral garden species displaying the minimum, mean, and maximum alpha diversity value for Shannon, Observed, Chao1 and Simpson.

taxon	Shannon			Observed			Chao1			Simpson		
	min.sh	mean.sh	max.sh	min.ob	mean.ob	max.ob	min.ch	mean.ch	max.ch	min.sim	mean.sim	max.sim
***Alcyonium* sp.**	1.222	1.222	1.222	428.000	428.000	428.000	63.330	63.330	63.330	0.422	0.422	0.422
Antipathella subpinnata	2.741	2.741	2.741	777.000	777.000	777.000	53.080	53.080	53.080	0.655	0.655	0.655
Callogorgia verticillata	2.919	2.919	2.919	458.000	458.000	458.000	65.935	65.935	65.935	0.855	0.855	0.855
Dendrophyllia cornigera	2.150	2.439	2.827	620.000	683.333	716.000	53.081	58.721	65.811	0.642	0.708	0.828
Dendrophyllia ramea	3.374	3.978	4.492	885.000	937.667	1028.000	52.182	58.162	65.471	0.908	0.939	0.970
Ellisella paraplexauroides	4.159	4.409	4.861	799.000	931.667	1041.000	60.153	62.659	67.488	0.955	0.965	0.981
Eunicella verrucosa	1.343	1.445	1.563	333.000	374.667	398.000	56.354	69.199	77.637	0.450	0.476	0.508
Funiculina quadrangularis	1.411	2.260	3.278	623.000	673.667	707.000	59.563	66.811	81.085	0.467	0.627	0.804
Isidella* cf. *elongata	1.417	3.139	4.855	442.000	701.667	914.000	57.074	61.559	68.607	0.488	0.758	0.980
Leptogorgia sarmentosa	0.607	0.909	1.459	206.000	268.667	381.000	44.727	50.314	56.297	0.192	0.325	0.556
Paramuricea* cf. *grayi	0.674	0.898	1.331	224.000	349.667	522.000	46.426	56.944	76.180	0.209	0.264	0.368
***Paramuricea* sp.**	0.634	0.647	0.671	268.000	281.667	299.000	34.200	44.777	51.372	0.189	0.196	0.205
Pennatula rubra	2.926	3.263	3.654	680.000	720.667	782.000	48.162	56.029	62.046	0.855	0.895	0.932

Tab. 2. Alpha diversity metrices for all coral garden orders displaying the minimum, mean, and maximum alpha diversity value for Shannon, Observed, Chao1 and Simpson.

order	Shannon			Observed			Chao1			Simpson		
	min.sh	mean.sh	max.sh	min.ob	mean.ob	max.ob	min.ch	mean.ch	max.ch	min.sim	mean.sim	max.sim

**Antipatharia**	2.741	2.741	2.741	777.000	777.000	777.000	53.080	53.080	53.080	0.655	0.655	0.655
**Malacalcyonacea**	0.607	0.994	1.563	206.000	327.077	522.000	34.200	55.926	77.637	0.189	0.323	0.556
**Scleractinia**	2.150	3.209	4.492	620.000	810.500	1028.000	52.182	58.441	65.811	0.642	0.824	0.970
**Scleralcyonacea**	1.411	3.241	4.861	442.000	733.923	1041.000	48.162	62.085	81.085	0.467	0.815	0.981

Tab. 3. Results of one-way ANOVA comparing alpha diversity metrics (Observed richness, Shannon index, and Chao1) across coral garden species and orders within the natural microbiome. Degrees of freedom (Df), sum of squares (Sum_Sq), mean squares (Mean_Sq), F-values (F_value), and p-values (p_value) are reported. Significance levels are indicated as ***p < 0.001.

Approach	Taxa	Test	Metric	Df	Sum_Sq	Mean_Sq	F_value	p_value	significance
**Natural Microbiome**	species	ANOVA	Observed	12	1212709.394	101059.116	9.373	0.000	***
**Natural Microbiome**	species	ANOVA	Shannon	12	50.329	4.194	8.481	0.000	***
**Natural Microbiome**	species	ANOVA	Chao1	12	1950004.995	162500.416	5.667	0.000	***
**Natural Microbiome**	order	ANOVA	Observed	3	912545.497	304181.832	17.102	0.000	***
**Natural Microbiome**	order	ANOVA	Shannon	3	38.510	12.837	17.148	0.000	***
**Natural Microbiome**	order	ANOVA	Chao1	3	1372004.445	457334.815	11.518	0.000	***

Tab. 4. Results of Tukey's Honestly Significant Difference (TukeyHSD) post-hoc test for pairwise comparisons of alpha diversity metrics (Shannon index, Observed richness, and Chao1) across coral garden species and orders within the natural microbiome. The mean difference, lower and upper confidence intervals (Lower CI, Upper CI), p-values, and significance levels are reported. Significance levels are indicated as ***p < 0.001, **p < 0.01, and *p < 0.05.

Approach	Taxon	Metric	Comparison	Difference	Lower CI	Upper CI	P-Value	Significance
**Natural Microbiome**	species	Shannon	Paramuricea grayi_p-Dendrophyllia ramea	-3.317	-5.461	-1.173	6.452E-04	***
**Natural Microbiome**	species	Shannon	Leptogorgia sarmentosa-Ellisella paraplexauroides	-3.498	-5.643	-1.354	3.297E-04	***
**Natural Microbiome**	species	Shannon	Paramuricea grayi-Ellisella paraplexauroides	-3.513	-5.657	-1.369	3.125E-04	***
**Natural Microbiome**	species	Shannon	Paramuricea grayi_p-Ellisella paraplexauroides	-3.762	-5.907	-1.618	1.262E-04	***
**Natural Microbiome**	species	Observed	Leptogorgia sarmentosa-Dendrophyllia ramea	-477.333	-793.962	-160.70	8.832E-04	***

**Natural Microbiome**	species	Observed	Paramuricea grayi_p-Dendrophyllia ramea	-492.333	-808.962	-175.70	6.050E-04	***
**Natural Microbiome**	species	Observed	Eunicella verrucosa-Ellisella paraplexauroides	-475.333	-791.962	-158.70	9.291E-04	***
**Natural Microbiome**	species	Observed	Leptogorgia sarmentosa-Ellisella paraplexauroides	-591.000	-907.629	-274.37	5.370E-05	***
**Natural Microbiome**	species	Observed	Paramuricea grayi-Ellisella paraplexauroides	-541.333	-857.962	-224.70	1.788E-04	***
**Natural Microbiome**	species	Observed	Paramuricea grayi_p-Ellisella paraplexauroides	-606.000	-922.629	-289.37	3.761E-05	***
**Natural Microbiome**	species	Chao1	Leptogorgia sarmentosa-Ellisella paraplexauroides	-793.508	-1309.86	-277.15	6.994E-04	***
**Natural Microbiome**	species	Chao1	Paramuricea grayi_p-Ellisella paraplexauroides	-776.432	-1292.79	-260.08	9.111E-04	***
**Natural Microbiome**	species	Shannon	Leptogorgia sarmentosa-Dendrophyllia ramea	-3.053	-5.197	-0.909	1.732E-03	**
**Natural Microbiome**	species	Shannon	Paramuricea grayi-Dendrophyllia ramea	-3.067	-5.212	-0.923	1.639E-03	**
**Natural Microbiome**	species	Shannon	Eunicella verrucosa-Ellisella paraplexauroides	-2.957	-5.102	-0.813	2.481E-03	**
**Natural Microbiome**	species	Shannon	Pennatula rubra-Paramuricea grayi_p	2.613	0.468	4.757	9.067E-03	**
**Natural Microbiome**	species	Observed	Paramuricea grayi-Dendrophyllia ramea	-427.667	-744.295	-111.04	3.123E-03	**
**Natural Microbiome**	species	Chao1	Paramuricea grayi-Ellisella paraplexauroides	-735.486	-1251.84	-219.13	1.723E-03	**
**Natural Microbiome**	species	Shannon	Ellisella paraplexauroides-Alcyonium sp.	3.189	0.156	6.221	3.378E-02	*
**Natural Microbiome**	species	Shannon	Eunicella verrucosa-Dendrophyllia ramea	-2.512	-4.656	-0.367	1.320E-02	*
**Natural Microbiome**	species	Shannon	Funiculina quadrangularis-Ellisella paraplexauroides	-2.156	-4.300	-0.012	4.797E-02	*
**Natural Microbiome**	species	Shannon	Leptogorgia sarmentosa-Isidella elongata	-2.223	-4.367	-0.078	3.791E-02	*
**Natural Microbiome**	species	Shannon	Paramuricea grayi-Isidella elongata	-2.237	-4.381	-0.093	3.599E-02	*
**Natural Microbiome**	species	Shannon	Paramuricea grayi_p-Isidella elongata	-2.487	-4.631	-0.342	1.450E-02	*

**Natural Microbiome**	species	Shannon	Pennatula rubra-Leptogorgia sarmentosa	2.349	0.204	4.493	2.406E-02	*
**Natural Microbiome**	species	Shannon	Pennatula rubra-Paramuricea grayi	2.363	0.219	4.507	2.282E-02	*
**Natural Microbiome**	species	Observed	Ellisella paraplexauroides-Alcyonium sp.	524.667	76.886	972.447	1.317E-02	*
**Natural Microbiome**	species	Observed	Ellisella paraplexauroides-Dendrophyllia cornigera	330.333	13.705	646.962	3.600E-02	*
**Natural Microbiome**	species	Observed	Eunicella verrucosa-Dendrophyllia ramea	-361.667	-678.295	-45.038	1.664E-02	*
**Natural Microbiome**	species	Observed	Leptogorgia sarmentosa-Isidella elongata	-346.000	-662.629	-29.371	2.454E-02	*
**Natural Microbiome**	species	Observed	Paramuricea grayi_p-Isidella elongata	-361.000	-677.629	-44.371	1.691E-02	*
**Natural Microbiome**	species	Observed	Pennatula rubra-Leptogorgia sarmentosa	365.333	48.705	681.962	1.518E-02	*
**Natural Microbiome**	species	Observed	Pennatula rubra-Paramuricea grayi_p	380.333	63.705	696.962	1.041E-02	*
**Natural Microbiome**	species	Chao1	Leptogorgia sarmentosa-Dendrophyllia ramea	-622.370	-1138.73	-106.01	1.007E-02	*
**Natural Microbiome**	species	Chao1	Paramuricea grayi-Dendrophyllia ramea	-564.348	-1080.70	-47.991	2.451E-02	*
**Natural Microbiome**	species	Chao1	Paramuricea grayi_p-Dendrophyllia ramea	-605.294	-1121.65	-88.938	1.311E-02	*
**Natural Microbiome**	species	Chao1	Eunicella verrucosa-Ellisella paraplexauroides	-596.932	-1113.29	-80.575	1.491E-02	*
**Natural Microbiome**	order	Shannon	Scleractinia-Malacalcyonacea	2.197	1.034	3.361	9.551E-05	***
**Natural Microbiome**	order	Shannon	Scleralcyonacea-Malacalcyonacea	2.243	1.318	3.167	1.764E-06	***
**Natural Microbiome**	order	Observed	Scleractinia-Malacalcyonacea	329.205	149.872	508.539	1.419E-04	***
**Natural Microbiome**	order	Observed	Scleralcyonacea-Malacalcyonacea	343.538	201.019	486.058	1.970E-06	***
**Natural Microbiome**	order	Chao1	Scleralcyonacea-Malacalcyonacea	423.903	210.960	636.846	4.441E-05	***
**Natural Microbiome**	order	Chao1	Scleractinia-Malacalcyonacea	395.087	127.139	663.034	2.049E-03	**

Tab. 5. Estimated marginal means (emmean) from Tukey's Honestly Significant Difference (TukeyHSD) test, showing overall differences in alpha diversity among species within the natural microbiome. The estimated mean (emmean), standard error (SE), degrees of freedom (df), and lower and upper confidence limits (lower.CL, upper.CL) are reported.

Taxon	emmean	SE	df	lower.CL	upper.CL
Ellisella paraplexauroides	4.406	0.406	20	3.559	5.253
Dendrophyllia ramea	3.960	0.406	20	3.114	4.807
Pennatula rubra	3.256	0.406	20	2.409	4.103
Isidella* cf. *elongata	3.130	0.406	20	2.283	3.977
Callogorgia verticillata	2.923	0.703	20	1.456	4.389
Antipathella subpinnata	2.743	0.703	20	1.276	4.210
Dendrophyllia cornigera	2.418	0.406	20	1.571	3.265
Funiculina quadrangularis	2.250	0.406	20	1.403	3.097
Eunicella verrucosa	1.449	0.406	20	0.602	2.296
***Alcyonium* sp.**	1.217	0.703	20	-0.250	2.684
Leptogorgia sarmentosa	0.907	0.406	20	0.061	1.754
Paramuricea* cf. *grayi	0.893	0.406	20	0.046	1.740
***Paramuricea* sp.**	0.644	0.406	20	-0.203	1.490

Tab. 6. Pairwise species comparisons from Tukey's Honestly Significant Difference (TukeyHSD) test, showing significant differences in alpha diversity between coral garden species. The contrast (species pair), estimated difference (estimate), standard error (SE), degrees of freedom (df), t-ratio (t.ratio), and p-value (p.value) are reported. Significant differences ($p < 0.05$) are highlighted.

contrast	estimate	SE	df	t.ratio	p.value
***Ellisella paraplexauroides* - *Paramuricea* sp.**	3.762	0.574	20	6.553	0.000
Ellisella paraplexauroides* - *Paramuricea* cf. *grayi	3.513	0.574	20	6.119	0.000
Ellisella paraplexauroides* - *Leptogorgia sarmentosa	3.498	0.574	20	6.093	0.000
***Dendrophyllia ramea* - *Paramuricea* sp.**	3.317	0.574	20	5.777	0.001
Dendrophyllia ramea* - *Paramuricea* cf. *grayi	3.067	0.574	20	5.343	0.002
Dendrophyllia ramea* - *Leptogorgia sarmentosa	3.053	0.574	20	5.317	0.002

Ellisella paraplexauroides* - *Eunicella verrucosa	2.957	0.574	20	5.151	0.002
Dendrophyllia ramea* - *Eunicella verrucosa	2.512	0.574	20	4.375	0.013
***Isidella* cf. *elongata* - *Paramuricea* sp.**	2.487	0.574	20	4.331	0.014
Isidella* cf. *elongata* - *Paramuricea* cf. *grayi	2.237	0.574	20	3.896	0.036
Isidella* cf. *elongata* - *Leptogorgia sarmentosa	2.223	0.574	20	3.871	0.038
Ellisella paraplexauroides* - *Funiculina quadrangularis	2.156	0.574	20	3.755	0.048
Leptogorgia sarmentosa* - *Pennatula rubra	-2.349	0.574	20	-4.091	0.024
Paramuricea* cf. *grayi* - *Pennatula rubra	-2.363	0.574	20	-4.116	0.023
Paramuricea* sp.- *Pennatula rubra	-2.613	0.574	20	-4.550	0.009
Alcyonium* sp. - *Ellisella paraplexauroides	-3.189	0.812	20	-3.927	0.034

Tab. 7. Estimated marginal means (emmean) from Tukey's Honestly Significant Difference (TukeyHSD) test, showing overall differences in alpha diversity among orders within the natural microbiome. The estimated mean (emmean), standard error (SE), degrees of freedom (df), and lower and upper confidence limits (lower.CL, upper.CL) are reported.

Order	emmean	SE	df	lower.CL	upper.CL
**Scleralcyonacea**	3.235	0.240	29	2.744	3.725
**Scleractinia**	3.189	0.353	29	2.467	3.911
**Antipatharia**	2.743	0.865	29	0.973	4.512
**Malacalcyonacea**	0.992	0.240	29	0.501	1.483

Tab. 8. Pairwise species comparisons from Tukey's Honestly Significant Difference (TukeyHSD) test, showing significant differences in alpha diversity between coral garden orders. The contrast (coral order pair), estimated difference (estimate), standard error (SE), degrees of freedom (df), t-ratio (t.ratio), and p-value (p.value) are reported. Significant differences ($p < 0.05$) are highlighted.

contrast	estimate	SE	df	t.ratio	p.value
**Malacalcyonacea - Scleractinia**	-2.197	0.427	29	-5.145	0.000
**Malacalcyonacea - Scleralcyonacea**	-2.243	0.339	29	-6.608	0.000
**Malacalcyonacea _ all_other**	2.22	0.035			

Tab. 9. PERMANOVA and PERMDISP results for the natural microbiome, testing the effects of species and taxonomic order on microbial community composition. The degrees of freedom (Df), sum of squares (SumOfSqs), mean square (Mean Sq), coefficient of determination (R^2), F-value (F), and p-value (p-value) are reported. Asterisks indicate significance levels (*** $p < 0.001$).

Approach	Taxa	Test	Df	SumOfSqs	Mean Sq	R2	F	p-value	significance
**Natural Microbiome**	species * order	PERMANOVA	12	7.962	NA	0.737	4.661	0.000	***
**Natural Microbiome**	species * order	PERMDISP	3	1.189	0.396	NA	16.916	0.000	***

Tab. 10. The ten most abundant microbes (OTUs) found across all coral garden species, including their taxonomic classification (OTU, Phylum, Family), as well as summary statistics (mean abundance [MEAN], median [MEDIAN], standard deviation [SD], interquartile range [IQR], and standard error [SE]).

Nr.	OTU	Phylum	Family	MEAN	MEDIAN	SD	IQR	SE
**1**	50381	Proteobacteria	Endozoicomonadaceae	32.725	6.005	38.889	72.884	6.770
**2**	50471	Proteobacteria	Spongiibacteraceae	5.210	1.022	9.584	4.267	1.668
**3**	46622	Spirochaetota	Spirochaetaceae	5.154	0.020	16.430	0.036	2.860
**4**	45267	Firmicutes	Mycoplasmataceae	4.333	0.032	16.104	0.215	2.803
**5**	2666	Proteobacteria	Rhodobacteraceae	3.697	0.115	12.415	0.712	2.161
**6**	2638	Proteobacteria	Rhodobacteraceae	2.952	0.016	10.914	0.064	1.900
**7**	3723	Proteobacteria	Pseudomonadaceae	2.886	1.845	3.460	2.422	0.602
**8**	44221	Bacteroidota	Flavobacteriaceae	2.005	0.119	6.244	1.118	1.087
**9**	44166	Bacteroidota	Blattabacteriaceae	1.771	0.000	10.072	0.008	1.753
**10**	44294	Bacteroidota	Flavobacteriaceae	1.518	0.036	5.814	0.123	1.012

Tab. 11. Relative abundance of the most abundant microbial families per coral garden species. The table includes the taxonomic classification (OTU, taxon, order, phylum, family) as well as summary statistics (mean abundance [MEAN], median [MEDIAN], standard deviation [SD], interquartile range [IQR], and standard error [SE]) for each microbial family across species.

OTU	taxon	order	Phylum	Family	MEAN	MEDIAN	SD	IQR	SE

**46622**	*Alcyonium* sp.	Malacalcyonacea	Spirochaetota	Spirochaetaceae	75.084	75.084	NA	0	NA
**50381**	*Alcyonium* sp.	Malacalcyonacea	Proteobacteria	Endozoicomonadaceae	11.072	11.072	NA	0	NA
**11460**	*Alcyonium* sp.	Malacalcyonacea	Proteobacteria	Rhodobacteraceae	3.894	3.894	NA	0	NA
**44166**	*Antipathella subpinnata*	Antipatharia	Bacteroidota	Blattabacteriaceae	57.875	57.875	NA	0	NA
**50258**	*Antipathella subpinnata*	Antipatharia	Proteobacteria	Colwelliaceae	2.422	2.422	NA	0	NA
**44221**	*Antipathella subpinnata*	Antipatharia	Bacteroidota	Flavobacteriaceae	2.343	2.343	NA	0	NA
**44294**	*Callogorgia verticillata*	Scleralcyonacea	Bacteroidota	Flavobacteriaceae	32.628	32.628	NA	0	NA
**50258**	*Callogorgia verticillata*	Scleralcyonacea	Proteobacteria	Colwelliaceae	16.199	16.199	NA	0	NA
**50351**	*Callogorgia verticillata*	Scleralcyonacea	Proteobacteria	Vibrionaceae	7.425	7.425	NA	0	NA
**2638**	*Dendrophyllia cornigera*	Scleractinia	Proteobacteria	Rhodobacteraceae	31.123	19.154	24.370	22.055	14.070
**46622**	*Dendrophyllia cornigera*	Scleractinia	Spirochaetota	Spirochaetaceae	18.845	0.040	32.575	28.212	18.807
**44221**	*Dendrophyllia cornigera*	Scleractinia	Bacteroidota	Flavobacteriaceae	13.590	2.768	18.978	16.503	10.957
**2785**	*Dendrophyllia ramea*	Scleractinia	Proteobacteria	Rickettsiaceae	9.285	9.808	8.392	8.380	4.845
**45198**	*Dendrophyllia ramea*	Scleractinia	Firmicutes	Spiroplasmataceae	7.389	0.883	12.042	10.643	6.953
**2761**	*Dendrophyllia ramea*	Scleractinia	Proteobacteria		6.452	6.968	5.953	5.936	3.437
**2666**	*Ellisella paraplexauroides*	Scleralcyonacea	Proteobacteria	Rhodobacteraceae	7.171	6.157	5.317	5.244	3.070
**46179**	*Ellisella paraplexauroides*	Scleralcyonacea	Proteobacteria	Fokiniaceae	5.311	1.042	8.305	7.437	4.795
**50471**	*Ellisella paraplexauroides*	Scleralcyonacea	Proteobacteria	Spongiibacteraceae	3.984	3.878	3.414	3.412	1.971
**50381**	*Eunicella verrucosa*	Malacalcyonacea	Proteobacteria	Endozoicomonadaceae	71.543	72.136	2.542	2.490	1.468
**50471**	*Eunicella verrucosa*	Malacalcyonacea	Proteobacteria	Spongiibacteraceae	7.770	5.469	4.818	4.387	2.782
**44194**	*Eunicella verrucosa*	Malacalcyonacea	Bacteroidota	Flavobacteriaceae	2.487	0.955	2.853	2.525	1.647
**45267**	*Funiculina quadrangularis*	Scleralcyonacea	Firmicutes	Mycoplasmataceae	44.334	61.203	38.743	35.883	22.368
**50471**	*Funiculina quadrangularis*	Scleralcyonacea	Proteobacteria	Spongiibacteraceae	18.885	11.884	21.416	20.540	12.364
**46586**	*Funiculina quadrangularis*	Scleralcyonacea	SAR324 clade(Marine group B)		4.790	0.255	8.007	6.978	4.623
**2666**	*Isidella* cf. *elongata*	Scleralcyonacea	Proteobacteria	Rhodobacteraceae	30.919	12.154	33.875	29.723	19.558
**50381**	*Isidella* cf. *elongata*	Scleralcyonacea	Proteobacteria	Endozoicomonadaceae	19.598	16.219	20.503	20.293	11.837
**50258**	*Isidella* cf. *elongata*	Scleralcyonacea	Proteobacteria	Colwelliaceae	2.608	0.434	3.985	3.512	2.301
**50381**	*Leptogorgia sarmentosa*	Malacalcyonacea	Proteobacteria	Endozoicomonadaceae	80.640	87.914	14.245	12.776	8.224
**50471**	*Leptogorgia sarmentosa*	Malacalcyonacea	Proteobacteria	Spongiibacteraceae	6.416	2.494	7.976	7.216	4.605

**45190**	*Leptogorgia sarmentosa*	Malacalcyonacea	Firmicutes		2.730	0.306	4.465	3.941	2.578
**50381**	*Paramuricea* cf. *grayi*	Malacalcyonacea	Proteobacteria	Endozoicomonadaceae	85.730	88.614	5.441	4.834	3.141
**3723**	*Paramuricea* cf. *grayi*	Malacalcyonacea	Proteobacteria	Pseudomonadaceae	3.459	3.444	0.173	0.173	0.100
**50404**	*Paramuricea* cf. *grayi*	Malacalcyonacea	Proteobacteria	Halomonadaceae	1.440	1.416	0.188	0.187	0.109
**50381**	*Paramuricea* sp.	Malacalcyonacea	Proteobacteria	Endozoicomonadaceae	89.682	89.624	0.421	0.418	0.243
**3723**	*Paramuricea* sp.	Malacalcyonacea	Proteobacteria	Pseudomonadaceae	3.141	3.154	0.263	0.262	0.152
**50404**	*Paramuricea* sp.	Malacalcyonacea	Proteobacteria	Halomonadaceae	1.360	1.336	0.160	0.159	0.093
**50471**	*Pennatula rubra*	Scleralcyonacea	Proteobacteria	Spongiibacteraceae	16.771	17.710	14.758	14.735	8.520
**46622**	*Pennatula rubra*	Scleralcyonacea	Spirochaetota	Spirochaetaceae	12.373	15.340	10.736	10.424	6.198
**3723**	*Pennatula rubra*	Scleralcyonacea	Proteobacteria	Pseudomonadaceae	11.603	8.964	5.273	4.753	3.045

Tab. 12. Relative abundance of the most abundant microbial families per coral garden order. The table includes the taxonomic classification (OTU, taxon, order, phylum, family) as well as summary statistics (mean abundance [MEAN], median [MEDIAN], standard deviation [SD], interquartile range [IQR], and standard error [SE]) for each microbial family across coral orders.

OTU	order	Phylum	Family	MEAN	MEDIAN	SD	IQR	SE
**44166**	Antipatharia	Bacteroidota	Blattabacteriaceae	57.875	57.875	NA	0	NA
**50258**	Antipatharia	Proteobacteria	Colwelliaceae	2.422	2.422	NA	0	NA
**44221**	Antipatharia	Bacteroidota	Flavobacteriaceae	2.343	2.343	NA	0	NA
**26226**	Antipatharia	Proteobacteria	Rickettsiaceae	1.571	1.571	NA	0	NA
**50273**	Antipatharia	Proteobacteria	Kangiellaceae	1.336	1.336	NA	0	NA
**50381**	Malacalcyonacea	Proteobacteria	Endozoicomonadaceae	76.450	87.914	21.720	17.157	6.024
**46622**	Malacalcyonacea	Spirochaetota	Spirochaetaceae	5.786	0.008	20.821	0.020	5.775
**50471**	Malacalcyonacea	Proteobacteria	Spongiibacteraceae	3.378	0.469	5.247	4.359	1.455
**3723**	Malacalcyonacea	Proteobacteria	Pseudomonadaceae	2.211	2.871	1.222	2.422	0.339
**45190**	Malacalcyonacea	Firmicutes		0.855	0.000	2.244	0.076	0.622
**2638**	Scleractinia	Proteobacteria	Rhodobacteraceae	15.674	7.823	22.892	17.933	9.345
**46622**	Scleractinia	Spirochaetota	Spirochaetaceae	9.434	0.038	23.037	0.016	9.405
**44221**	Scleractinia	Bacteroidota	Flavobacteriaceae	9.391	4.600	12.880	3.109	5.258
**2785**	Scleractinia	Proteobacteria	Rickettsiaceae	4.645	0.330	7.349	7.513	3.000

**45198**	Scleractinia	Firmicutes	Spiroplasmataceae	3.721	0.076	8.612	0.681	3.516
**45267**	Scleralcyonacea	Firmicutes	Mycoplasmataceae	10.251	0.024	25.054	0.040	6.949
**50471**	Scleralcyonacea	Proteobacteria	Spongiibacteraceae	9.661	3.162	13.358	10.317	3.705
**2666**	Scleralcyonacea	Proteobacteria	Rhodobacteraceae	8.881	0.457	19.037	10.488	5.280
**50381**	Scleralcyonacea	Proteobacteria	Endozoicomonadaceae	6.159	1.400	11.527	5.373	3.197
**3723**	Scleralcyonacea	Proteobacteria	Pseudomonadaceae	4.679	2.525	4.866	6.463	1.350

2.2.2 Statistics for 2) Captivity

Short-Term

Tab. 13. Alpha diversity metrices for all sampling days of the short-term approach for *E. verrucosa* and *P.* cf. *grayi* displaying the minimum, mean, and maximum alpha diversity value for Shannon, Observed, Chao1 and Simpson.

NMS_time_txt	taxon	Shannon			Observed			Chao1			Simpson		
		min.sh	mean.sh	max.sh	min.ob	mean.ob	max.ob	min.ch	mean.ch	max.ch	min.sim	mean.sim	max.sim
**t_0**	*Eunicella verrucosa*	0.238	0.590	0.892	216	332.500	432	391.526	566.329	761.508	0.061	0.185	0.320
**t_0**	*Paramuricea* cf. *grayi*	0.658	0.859	1.098	306	373.667	417	510.064	599.202	700.099	0.200	0.253	0.323
**t_4**	*Eunicella verrucosa*	0.283	0.510	0.641	214	269.250	298	424.886	495.065	565.750	0.076	0.158	0.225
**t_4**	*Paramuricea* cf. *grayi*	0.687	1.406	2.751	358	524.667	767	544.667	757.863	992.780	0.211	0.379	0.698
**t_45**	*Eunicella verrucosa*	0.354	0.598	0.850	224	290.750	363	426.784	516.985	587.845	0.111	0.196	0.303
**t_45**	*Paramuricea* cf. *grayi*	0.687	0.778	0.824	302	344.333	386	457.948	535.862	632.356	0.215	0.240	0.261

Tab. 14. ANOVA and Kruskal-Wallis (KW) test results for short-term temporal changes in microbial diversity across species (*Eunicella verrucosa* and *Paramuricea* cf. *grayi*) and sampling days. The table presents test statistics (F-value, Chi-Square [Chi_Sq]), degrees of freedom (Df), sum of squares (Sum_Sq), mean square (Mean_Sq), and p-values for three diversity metrics (Observed, Shannon, and Chao1) across different species (*Eunicella verrucosa* and *Paramuricea* cf. *grayi*) and environmental conditions (water). Significant results are marked with asterisks (* for $p \leq 0.05$, ** for $p \leq 0.01$, *** for $p \leq 0.001$).

Approach	Taxa	Test	Metric	Df	Sum_Sq	Mean_Sq	Chi_Sq	F_value	p_value	significance
**Short term / Time**	*Eunicella verrucosa*	ANOVA	Observed	3	42463.767	14154.589	NA	4.261	0.032	*
**Short term / Time**	*Eunicella verrucosa*	ANOVA	Shannon	3	1.885	0.628	NA	14.114	0.000	***
**Short term / Time**	*Eunicella verrucosa*	ANOVA	Chao1	3	94740.484	31580.161	NA	3.243	0.064	.
**Short term / Time**	*Paramuricea* cf. *grayi*	ANOVA	Observed	3	34134	11378	NA	0.671	0.593	NONE
**Short term / Time**	*Paramuricea* cf. *grayi*	ANOVA	Shannon	3	0.744	0.248	NA	0.626	0.618	NONE
**Short term / Time**	*Paramuricea* cf. *grayi*	ANOVA	Chao1	3	68906.713	22968.904	NA	0.751	0.552	NONE
**Short term / Time**	*Paramuricea* cf. *grayi*	Kruskal-Wallis	Observed	3	NA	NA	1.051	NA	0.789	NONE

**Short term / Time**	*Paramuricea* cf. *grayi*	Kruskal-Wallis	Shannon	3	NA	NA	0.538	NA	0.910	NONE
**Short term / Time**	*Paramuricea* cf. *grayi*	Kruskal-Wallis	Chao1	3	NA	NA	1.923	NA	0.589	NONE
**Short term / Time**	water	Kruskal-Wallis	Observed	11	NA	NA	11.00	NA	0.443	NONE
**Short term / Time**	water	Kruskal-Wallis	Shannon	11	NA	NA	11.00	NA	0.443	NONE
**Short term / Time**	water	Kruskal-Wallis	Chao1	11	NA	NA	11.00	NA	0.443	NONE

Tab. 15. Results of Tukey's Honestly Significant Difference (TukeyHSD) post-hoc test for pairwise comparisons of alpha diversity metrics (Shannon index, Observed richness, and Chao1) for *Eunicella verrucosa* over short-term (45 days). The mean difference, lower and upper confidence intervals (Lower CI, Upper CI), p-values, and significance levels are reported. Significance levels are indicated as ***p < 0.001, **p < 0.01, and *p < 0.05.

Approach	Taxon	Metric	Comparison	Difference	Lower CI	Upper CI	P-Value	Significance
**Short-term/ Time**	*Eunicella verrucosa*	Shannon	wild-t_4	0.944	0.459	1.429	5.396E-04	***
**Short-term/ Time**	*Eunicella verrucosa*	Shannon	wild-t_0	0.853	0.368	1.338	1.233E-03	**
**Short-term/ Time**	*Eunicella verrucosa*	Shannon	wild-t_45	0.844	0.359	1.329	1.347E-03	**
**Short-term/ Time**	*Eunicella verrucosa*	Observed	wild-t_4	149.583	17.106	282.061	2.612E-02	*

Tab. 16. Estimated marginal means (emmean) from Tukey's Honestly Significant Difference (TukeyHSD) test, showing overall differences in alpha diversity among sampling days from the short-term approach (45 days) for *E. verrucosa*. The estimated mean (emmean), standard error (SE), degrees of freedom (df), and lower and upper confidence limits (lower.CL, upper.CL) are reported.

Time	emmean	SE	df	lower.CL	upper.CL
**wild**	1.449	0.122	11	1.181	1.717
**t_45**	0.605	0.105	11	0.372	0.837
**t_0**	0.595	0.105	11	0.363	0.827
**t_4**	0.505	0.105	11	0.272	0.737

Tab. 17. Pairwise species comparisons from Tukey's Honestly Significant Difference (TukeyHSD) test, showing significant differences in alpha diversity over short-term (45 days) in *E. verrucosa*. The contrast (sampling day pair), estimated difference (estimate), standard error (SE), degrees of freedom (df), t-ratio (t.ratio), and p-value (p.value) are reported. Significant differences (p < 0.05) are highlighted.

contrast	estimate	SE	df	t.ratio	p.value
**t_45 - wild**	-0.844	0.161	11	-5.237	0.001
**t_0 - wild**	-0.853	0.161	11	-5.296	0.001
**t_4 - wild**	-0.944	0.161	11	-5.858	0.001
**wild_all_other**	0.880	0.055			

Tab. 18. PERMANOVA and PERMDISP results, testing the effects of sampling days (short-term) on the microbial community composition in *E. verrucosa* and *P.* cf. *grayi*. The degrees of freedom (Df), sum of squares (SumOfSqs), mean square (Mean Sq), coefficient of determination (R^2), F-value (F), and p-value (p-value) are reported. Asterisks indicate significance levels (*** p < 0.001).

Approach	Taxa	Test	Df	SumOfSqs	Mean Sq	R2	F	p-value	significance
**Short term / Time**	*Eunicella verrucosa*	PERMANOVA	3	0.080	NA	0.535	4.215	0.003	**
**Short term / Time**	*Eunicella verrucosa*	PERMDISP	3	0.010	0.003	NA	3.639	0.066	.
**Short term / Time**	*Paramuricea* cf. *grayi*	PERMANOVA	3	0.029	NA	0.242	0.852	0.779	NONE
**Short term / Time**	*Paramuricea* cf. *grayi*	PERMDISP	3	0.018	0.006	NA	0.647	0.668	NONE
**Short term / Time**	water	PERMANOVA	NA	NA	NA	NA	NA	NA	NA
**Short term / Time**	water	PERMDISP	NA	NA	NA	NA	NA	NA	NA

Long-Term

Tab. 19. Alpha diversity metrices for all sampling locations of the long-term approach for *E. verrucosa* and *P.* cf. *grayi* displaying the minimum, mean, and maximum alpha diversity value for Shannon, Observed, Chao1 and Simpson.

location	taxon	Shannon			Observed			Chao1			Simpson		
		min.sh	mean.sh	max.sh	min.ob	mean.ob	max.ob	min.ch	mean.ch	max.ch	min.sim	mean.sim	max.sim
**Ramalhete**	*Eunicella verrucosa*	0.356	0.602	0.855	238	308.750	389	489.784	578.389	680.610	0.112	0.197	0.304
**Ramalhete**	*Paramuricea* cf. *grayi*	0.691	0.784	0.831	325	379.667	425	526.500	648.259	774.525	0.215	0.241	0.262
**Oceanario de lisboa**	*Eunicella verrucosa*	2.971	3.524	4.340	626	725.667	819	829.733	1031.681	1231.919	0.789	0.860	0.964
**Oceanario de lisboa**	*Paramuricea* cf. *grayi*	2.876	3.244	3.523	480	541.667	659	714.373	829.369	1002.688	0.859	0.904	0.944
**Zoomarine**	*Eunicella verrucosa*	0.320	0.528	0.850	199	273.000	358	425.111	489.821	592.231	0.098	0.170	0.285
**Zoomarine**	*Paramuricea* cf. *grayi*	1.153	2.018	3.310	424	614.000	823	646.075	908.478	1148.684	0.365	0.537	0.785

Tab. 20. ANOVA and Kruskal-Wallis (KW) test results for long-term differences between sampling locations in microbial diversity across *E. verrucosa* and *P.* cf. *grayi* and sampling locations. The table presents test statistics (F-value, Chi-Square [Chi_Sq]), degrees of freedom (Df), sum of squares (Sum_Sq), mean square (Mean_Sq), and p-values for three diversity metrics (Observed, Shannon, and Chao1) across different species (*Eunicella verrucosa* and *Paramuricea* cf. *grayi*) and environmental conditions (water). Significant results are marked with asterisks (* for p ≤ 0.05, ** for p ≤ 0.01, *** for p ≤ 0.001).

Approach	Taxa	Test	Metric	Df	Sum_Sq	Mean_Sq	Chi_Sq	F_value	p_value	significance
**Long-term/Location**	*Eunicella verrucosa*	ANOVA	Observed	3	256109	85370	NA	22.0	0.000	***
**Long-term/Location**	*Eunicella verrucosa*	ANOVA	Shannon	3	18.317	6.106	NA	41.38	0.000	***
**Long-term/Location**	*Eunicella verrucosa*	ANOVA	Chao1	3	437823.727	145941.242	NA	10.13	0.003	**
**Long-term/Location**	*Eunicella verrucosa*	Kruskal-Wallis	Observed	3	NA	NA	10.236	NA	0.017	*
**Long-term/Location**	*Eunicella verrucosa*	Kruskal-Wallis	Shannon	3	NA	NA	10.044	NA	0.018	*

**Long-term/Location**	*Eunicella verrucosa*	Kruskal-Wallis	Chao1	3	NA	NA	7.714	NA	0.052	.
**Long-term/Location**	*Paramuricea cf. grayi*	ANOVA	Observed	3	118314.000	39438.000	NA	2.387	0.145	NONE
**Long-term/Location**	*Paramuricea cf. grayi*	ANOVA	Shannon	3	11.867	3.956	NA	10.293	0.004	**
**Long-term/Location**	*Paramuricea cf. grayi*	ANOVA	Chao1	3	212175.029	70725.010	NA	2.181	0.168	NONE
**Long-term/Location**	water	Kruskal-Wallis	Observed	4	NA	NA	4.000	NA	0.406	NONE
**Long-term/Location**	water	Kruskal-Wallis	Shannon	4	NA	NA	4.000	NA	0.406	NONE
**Long-term/Location**	water	Kruskal-Wallis	Chao1	4	NA	NA	4.000	NA	0.406	NONE

Tab. 21. Results of Tukey's Honestly Significant Difference (TukeyHSD) post-hoc test for pairwise comparisons of alpha diversity metrics (Shannon index, Observed richness, and Chao1) for *E. verrucosa* and *P.* cf. *grayi* over long-term, between different sampling locations. The mean difference, lower and upper confidence intervals (Lower CI, Upper CI), p-values, and significance levels are reported. Significance levels are indicated as ***p < 0.001, **p < 0.01, and *p < 0.05.

Approach	Taxon	Metric	Comparison	Difference	Lower CI	Upper CI	P-Value	Significance
**Long-term/ Location**	*Eunicella verrucosa*	Shannon	Oceanario_lisboa-Faro_ramalhete	2.921	2.005	3.837	1.799E-05	***
**Long-term/ Location**	*Eunicella verrucosa*	Shannon	Sagres-Oceanario_lisboa	-2.077	-3.056	-1.098	4.569E-04	***
**Long-term/ Location**	*Eunicella verrucosa*	Shannon	Zoomarine_albufeira-Oceanario_lisboa	-2.995	-3.974	-2.016	2.535E-05	***
**Long-term/ Location**	*Eunicella verrucosa*	Observed	Oceanario_lisboa-Faro_ramalhete	335.083	186.544	483.623	2.863E-04	***
**Long-term/ Location**	*Eunicella verrucosa*	Observed	Zoomarine_albufeira-Oceanario_lisboa	-362.333	-521.129	-203.54	2.623E-04	***
**Long-term/ Location**	*Eunicella verrucosa*	Chao1	Oceanario_lisboa-Faro_ramalhete	443.824	157.619	730.029	4.167E-03	**
**Long-term/ Location**	*Eunicella verrucosa*	Chao1	Zoomarine_albufeira-Oceanario_lisboa	-459.476	-765.442	-153.51	5.132E-03	**
**Long-term/ Location**	*Eunicella verrucosa*	Observed	Sagres-Oceanario_lisboa	-211.000	-369.795	-52.205	1.096E-02	*
**Long-term/ Location**	*Paramuricea cf. grayi*	Shannon	Oceanario_lisboa-Faro_ramalhete	2.459	0.838	4.080	5.506E-03	**

Long-term/ Location	*Paramuricea cf. grayi*	Shannon	Sagres-Oceanario_lisboa	-2.344	-3.965	-0.723	7.322E-03	**

Tab. 22. Estimated marginal means (emmean) from Tukey's Honestly Significant Difference (TukeyHSD) test, showing overall differences in alpha diversity among sampling locations (long-term) for *E. verrucosa* and *P.* cf. *grayi*. The estimated mean (emmean), standard error (SE), degrees of freedom (df), and lower and upper confidence limits (lower.CL, upper.CL) are reported.

Location	emmean	SE	df	lower.CL	upper.CL
Eunicella verrucosa					
**Oceanario_lisboa**	3.525	0.222	9	3.024	4.027
**Sagres**	1.449	0.222	9	0.947	1.950
**Faro_ramalhete**	0.605	0.192	9	0.170	1.039
**Zoomarine_albufeira**	0.530	0.222	9	0.028	1.032
Paramuricea* cf. *grayi					
**Oceanario_lisboa**	3.237	0.358	8	2.411	4.062
**Zoomarine_albufeira**	2.009	0.358	8	1.184	2.835
**Sagres**	0.893	0.358	8	0.068	1.718
**Faro_ramalhete**	0.778	0.358	8	-0.048	1.603

Tab. 23. Pairwise species comparisons from Tukey's Honestly Significant Difference (TukeyHSD) test, showing significant differences in alpha diversity over long-term, across different sampling locations for *E. verrucosa* and *P.* cf. *grayi*. The contrast (sampling location pair), estimated difference (estimate), standard error (SE), degrees of freedom (df), t-ratio (t.ratio), and p-value (p.value) are reported. Significant differences ($p < 0.05$) are highlighted.

contrast	estimate	SE	df	t.ratio	p.value
Eunicella verrucosa					
**Oceanario_lisboa - Zoomarine_albufeira**	2.995	0.314	9	9.550	0.000
**Oceanario_lisboa - Sagres**	2.077	0.314	9	6.621	0.000
**Faro_ramalhete - Oceanario_lisboa**	-2.921	0.293	9	-9.955	0.000
**Oceanario_lisboa_all_other**	2.664	0.634			
Paramuricea* cf. *grayi					

**Faro_ramalhete - Oceanario_lisboa**	-2.459	0.506	8	-4.858	0.006
**Oceanario_lisboa - Sagres**	2.344	0.506	8	4.630	0.007
**Oceanario_lisboa_all_other**	2.402	0.081			

Tab. 24. PERMANOVA and PERMDISP results, testing the effects of sampling locations (long-term) on the microbial community composition in *E. verrucosa* and *P.* cf. *grayi*. The degrees of freedom (Df), sum of squares (SumOfSqs), mean square (Mean Sq), coefficient of determination (R^2), F-value (F), and p-value (p-value) are reported. Significance levels are indicated as ***$p < 0.001$, **$p < 0.01$, and *$p < 0.05$.

Approach	Taxa	Test	Df	SumOfSqs	Mean Sq	R2	F	p-value	significance
**Long-term/Location**	*Eunicella verrucosa*	PERMANOVA	3	1.117	NA	0.724	7.884	0.001	**
**Long-term/Location**	*Eunicella verrucosa*	PERMDISP	3	0.193	0.064	NA	15.037	0.001	***
**Long-term/Location**	*Paramuricea cf. grayi*	PERMANOVA	3	1.223	NA	0.661	5.195	0.003	**
**Long-term/Location**	*Paramuricea cf. grayi*	PERMDISP	3	0.280	0.093	NA	7.881	0.005	**
**Long-term/Location**	water	PERMANOVA	NA	NA	NA	NA	NA	NA	NA
**Long-term/Location**	water	PERMDISP	NA	NA	NA	NA	NA	NA	NA

Short – term

Tab. 25. Relative abundance of the most abundant microbial families per sampling day (short-term) for *E. verrucosa* and *P.* cf. *grayi*. The table includes the taxonomic classification (OTU, taxon, order, phylum, family) as well as summary statistics (mean abundance [MEAN], median [MEDIAN], standard deviation [SD], interquartile range [IQR], and standard error [SE]) for each microbial family across sampling days for both coral species.

OTU	taxon	NMS_time_txt	Phylum	Family	MEAN	MEDIAN	SD	IQR	SE
**50381**	*Eunicella verrucosa*	t_0	Proteobacteria	Endozoicomonadaceae	89.752	90.330	6.409	3.942	3.205
**50471**	*Eunicella verrucosa*	t_0	Proteobacteria	Spongiibacteraceae	4.637	3.424	4.751	5.548	2.376
**50381**	*Paramuricea* cf. *grayi*	t_0	Proteobacteria	Endozoicomonadaceae	86.283	87.305	3.689	3.581	2.130
**3723**	*Paramuricea* cf. *grayi*	t_0	Proteobacteria	Pseudomonadaceae	3.932	3.428	0.999	0.899	0.577
**50404**	*Paramuricea* cf. *grayi*	t_0	Proteobacteria	Halomonadaceae	1.376	1.277	0.237	0.221	0.137
**2666**	water	t_0	Proteobacteria	Rhodobacteraceae	11.168	11.168	NA	0	NA
**2653**	water	t_0	Proteobacteria	Rhodobacteraceae	4.633	4.633	NA	0	NA
**2558**	water	t_0	Proteobacteria	Rhodobacteraceae	4.371	4.371	NA	0	NA
**50381**	*Eunicella verrucosa*	t_4	Proteobacteria	Endozoicomonadaceae	91.589	91.234	3.434	2.819	1.717
**50471**	*Eunicella verrucosa*	t_4	Proteobacteria	Spongiibacteraceae	3.548	3.263	2.952	3.701	1.476
**50381**	*Paramuricea* cf. *grayi*	t_4	Proteobacteria	Endozoicomonadaceae	76.997	87.635	19.385	17.056	11.192
**3723**	*Paramuricea* cf. *grayi*	t_4	Proteobacteria	Pseudomonadaceae	3.802	3.870	0.128	0.113	0.074
**50404**	*Paramuricea* cf. *grayi*	t_4	Proteobacteria	Halomonadaceae	1.278	1.320	0.171	0.167	0.099
**2666**	water	t_4	Proteobacteria	Rhodobacteraceae	22.948	22.948	NA	0	NA
**2653**	water	t_4	Proteobacteria	Rhodobacteraceae	9.811	9.811	NA	0	NA
**2558**	water	t_4	Proteobacteria	Rhodobacteraceae	7.330	7.330	NA	0	NA
**2607**	water	t_4	Proteobacteria	Rhodobacteraceae	5.437	5.437	NA	0	NA
**50381**	*Eunicella verrucosa*	t_45	Proteobacteria	Endozoicomonadaceae	89.348	90.161	4.856	4.243	2.428
**50471**	*Eunicella verrucosa*	t_45	Proteobacteria	Spongiibacteraceae	4.735	4.075	3.583	3.890	1.792
**50381**	*Paramuricea* cf. *grayi*	t_45	Proteobacteria	Endozoicomonadaceae	86.869	86.561	1.409	1.384	0.814
**3723**	*Paramuricea* cf. *grayi*	t_45	Proteobacteria	Pseudomonadaceae	3.976	3.623	0.663	0.589	0.383

**50404**	*Paramuricea* cf. *grayi*	t_45	Proteobacteria	Halomonadaceae	1.583	1.698	0.393	0.380	0.227
**2666**	water	t_45	Proteobacteria	Rhodobacteraceae	7.612	7.612	NA	0	NA
**3765**	water	t_45	Proteobacteria	Thiotrichaceae	6.753	6.753	NA	0	NA
**26001**	water	t_45	Proteobacteria	Micavibrionaceae	5.258	5.258	NA	0	NA
**50381**	*Eunicella verrucosa*	wild	Proteobacteria	Endozoicomonadaceae	71.543	72.136	2.542	2.49	1.468
**50471**	*Eunicella verrucosa*	wild	Proteobacteria	Spongiibacteraceae	7.77	5.469	4.818	4.387	2.782
**44194**	*Eunicella verrucosa*	wild	Bacteroidota	Flavobacteriaceae	2.487	0.955	2.853	2.525	1.647
**26064**	*Eunicella verrucosa*	wild	Proteobacteria	Devosiaceae	2.299	1.559	1.92	1.81	1.108
**46091**	*Eunicella verrucosa*	wild	Planctomycetota	Pirellulaceae	2.138	0.016	3.687	3.196	2.128
**3723**	*Eunicella verrucosa*	wild	Proteobacteria	Pseudomonadaceae	1.222	1.094	0.601	0.591	0.347
**50381**	*Paramuricea* cf. *grayi*	wild	Proteobacteria	Endozoicomonadaceae	85.73	88.614	5.441	4.834	3.141
**3723**	*Paramuricea* cf. *grayi*	wild	Proteobacteria	Pseudomonadaceae	3.459	3.444	0.173	0.173	0.1
**50404**	*Paramuricea* cf. *grayi*	wild	Proteobacteria	Halomonadaceae	1.44	1.416	0.188	0.187	0.109
**50381**	*Paramuricea* cf. *grayi*	wild	Proteobacteria	Endozoicomonadaceae	71.543	72.136	2.542	2.49	1.468
**3716**	water	wild	Proteobacteria	Moraxellaceae	10.301	10.301	NA	0	NA
**44831**	water	wild	Cyanobacteria	Cyanobiaceae	10.181	10.181	NA	0	NA

Long – term

Tab. 26. Relative abundance of the most abundant microbial families per sampling location (long-term) for *E. verrucosa* and *P.* cf. *grayi*. The table includes the taxonomic classification (OTU, taxon, order, phylum, family) as well as summary statistics (mean abundance [MEAN], median [MEDIAN], standard deviation [SD], interquartile range [IQR], and standard error [SE]) for each microbial family across sampling locations for both coral species.

OTU	taxon	location	Phylum	Family	MEAN	MEDIAN	SD	IQR	SE
**50381**	*Eunicella verrucosa*	Faro_ramalhete	Proteobacteria	Endozoicomonadaceae	89.348	90.161	4.856	4.243	2.428
**50381**	*Paramuricea cf. grayi*	Faro_ramalhete	Proteobacteria	Endozoicomonadaceae	86.869	86.561	1.409	1.384	0.814
**2666**	water	Faro_ramalhete	Proteobacteria	Rhodobacteraceae	7.612	7.612	NA	0	NA
**3765**	water	Faro_ramalhete	Proteobacteria	Thiotrichaceae	6.753	6.753	NA	0	NA
**26001**	water	Faro_ramalhete	Proteobacteria	Micavibrionaceae	5.258	5.258	NA	0	NA
**2558**	water	Faro_ramalhete	Proteobacteria	Rhodobacteraceae	4.224	4.224	NA	0	NA
**3765**	water_O_EV	Oceanario_EV	Proteobacteria	Thiotrichaceae	15.157	15.157	NA	0	NA
**50351**	water_O_EV	Oceanario_EV	Proteobacteria	Vibrionaceae	8.225	8.225	NA	0	NA
**26321**	water_O_EV	Oceanario_EV	Proteobacteria	Arenicellaceae	4.252	4.252	NA	0	NA
**50321**	water_O_EV	Oceanario_EV	Proteobacteria	Pseudoalteromonadaceae	3.651	3.651	NA	0	NA
**26075**	water_O_EV	Oceanario_EV	Proteobacteria	Methyloligellaceae	2.740	2.740	NA	0	NA
**44294**	water_O_EV	Oceanario_EV	Bacteroidota	Flavobacteriaceae	2.685	2.685	NA	0	NA
**50471**	*Eunicella verrucosa*	Oceanario_lisboa	Proteobacteria	Spongiibacteraceae	17.941	7.370	19.059	16.716	11.003
**50381**	*Eunicella verrucosa*	Oceanario_lisboa	Proteobacteria	Endozoicomonadaceae	16.437	4.077	24.510	22.049	14.151
**50351**	*Paramuricea cf. grayi*	Oceanario_lisboa	Proteobacteria	Vibrionaceae	14.715	17.893	13.342	13.055	7.703
**50381**	*Paramuricea cf. grayi*	Oceanario_lisboa	Proteobacteria	Endozoicomonadaceae	12.097	6.614	15.322	14.568	8.846
**3765**	water_O_PG	Oceanario_PG	Proteobacteria	Thiotrichaceae	21.711	21.711	NA	0	NA
**50351**	water_O_PG	Oceanario_PG	Proteobacteria	Vibrionaceae	3.663	3.663	NA	0	NA
**26075**	water_O_PG	Oceanario_PG	Proteobacteria	Methyloligellaceae	3.313	3.313	NA	0	NA
**44294**	water_O_PG	Oceanario_PG	Bacteroidota	Flavobacteriaceae	2.856	2.856	NA	0	NA
**2666**	water_O_PG	Oceanario_PG	Proteobacteria	Rhodobacteraceae	2.227	2.227	NA	0	NA

**26321**	water_O_PG	Oceanario_PG	Proteobacteria	Arenicellaceae	2.183	2.183	NA	0	NA
**50381**	*Eunicella verrucosa*	wild	Proteobacteria	Endozoicomonadaceae	71.543	72.136	2.542	2.490	1.468
**50471**	*Eunicella verrucosa*	wild	Proteobacteria	Spongiibacteraceae	7.770	5.469	4.818	4.387	2.782
**50381**	*Paramuricea cf. grayi*	wild	Proteobacteria	Endozoicomonadaceae	85.730	88.614	5.441	4.834	3.141
**3716**	water	wild	Proteobacteria	Moraxellaceae	10.301	10.301	NA	0	NA
**44831**	water	wild	Cyanobacteria	Cyanobiaceae	10.181	10.181	NA	0	NA
**50381**	*Eunicella verrucosa*	Zoomarine_albufeira	Proteobacteria	Endozoicomonadaceae	90.904	93.458	5.737	5.294	3.312
**50381**	*Paramuricea cf. grayi*	Zoomarine_albufeira	Proteobacteria	Endozoicomonadaceae	66.147	73.167	17.863	16.797	10.313
**3723**	*Paramuricea cf. grayi*	Zoomarine_albufeira	Proteobacteria	Pseudomonadaceae	5.427	5.508	0.274	0.264	0.158
**44268**	water_zoo	Zoomarine_albufeira	Bacteroidota	Flavobacteriaceae	24.073	24.073	NA	0	NA
**2653**	water_zoo	Zoomarine_albufeira	Proteobacteria	Rhodobacteraceae	9.692	9.692	NA	0	NA
**2666**	water_zoo	Zoomarine_albufeira	Proteobacteria	Rhodobacteraceae	9.386	9.386	NA	0	NA
**2558**	water_zoo	Zoomarine_albufeira	Proteobacteria	Rhodobacteraceae	5.051	5.051	NA	0	NA
**2607**	water_zoo	Zoomarine_albufeira	Proteobacteria	Rhodobacteraceae	4.319	4.319	NA	0	NA
**44221**	water_zoo	Zoomarine_albufeira	Bacteroidota	Flavobacteriaceae	3.854	3.854	NA	0	NA
